# Statin treatment and muscle symptoms: series of randomised, placebo controlled n-of-1 trials

**DOI:** 10.1136/bmj.n135

**Published:** 2021-02-24

**Authors:** Emily Herrett, Elizabeth Williamson, Kieran Brack, Danielle Beaumont, Alexander Perkins, Andrew Thayne, Haleema Shakur-Still, Ian Roberts, Danielle Prowse, Ben Goldacre, Tjeerd van Staa, Thomas M MacDonald, Jane Armitage, Jon Wimborne, Paula Melrose, Jayshireen Singh, Lucy Brooks, Michael Moore, Maurice Hoffman, Liam Smeeth, StatinWISE Trial Group, Rebecca Harmston, Brian MacKenna, David Symes, John Norrie, Nicholas Mills, Hannah Castro, Nabila Youssouf, Collette Barrow, Sergey Kostrov, Hakim Miah, Eve Thacker, Melissa Baldey, Eleanor Sowerby, Nicola Harding, Gail Timcke, Alison Macleod, Karen Lomax, Nigel Wells, Emma Pierre, Tom Baker, Carla Bratten, Karen Forshaw, Daniel Clark, Selina Fox, Rachel Hubbard, David Crichton, Nabeel Alsindi, Mandy Hayes, Keith (Peter) Elliot, Robin Fox, Jane Stanford, Emily Ackland, George Strong, Debbie Kelly, Dev Malhotra, Dipti Gandhi, Gillian Foster, Diane Exley, Dawn Brayford, Theresa Nuttall, Clare Corbett, Nicola Anderton, Gwyn Hughes, Sian Turner, Sarah Roberts, David Brown, Susan Fairhead, Karen Sutcliffe, Mark Boon, Paula Dirienzo, Kay Ellor, Hasan Chowhan, Amy Townrow, Tracey Rowles, Debbie Hipps, Geoffrey Perry, Amanda Ayers, Rebecca Cooper, Sara Harley, Lesley Parsons, Ann Selby, Regan Hood, Elizabeth Zoon, Lucy Wraith, Vicky Peterson, Jackie Pretty, Narinder Dhillon, John Wearne, Sandra Moss, Kate Maitland, Catherine Edge, Susan Brown, Stuart Mackay-Thomas, Heather Pearson, Ewan Deas, Lesley Yelland, Helen Jones, Stephen Rogers, Ian Huckle, Carsten Dernedde, Caroline Mansfield, Heather Leishman, Jordan Howard, Chris Wright, Mark Ashworth, Satinder Kumar, Catarina Guerreiro, David Hartley, Sally Gordon, Carolyn Forrest, Andy Gibson, Laura Howe, John Whitwell, Irwin Nazareth, Letitia Coco-Bassey, Kate Walters, Jaqueline Mburu, Crystal Chetwood, Lynne Dowding, Alison Williams, Nikki Richards, Mini Nelson, Emma Chadwick, Helen Mingaye, Mehul Mathukia, Jacqueline Mburu, Anna Swinburn, Janeth Tomakin, Valentina Valasevich, Hywel Jones, Joanne Bannister, Emma Edwards, Morag McDowall, Beverley Hall, Helen Permain, Nigel Peacock, Carol Harrison, Lorraine Parsons, Chuin Kee, Paula McLaren, Sherard Le Maitre, Helen Nash, Stephanie Evans, Rachel Evans, Stephen Miller, Pooja Agarwal, Oliver Booth, Victoria Mayhew, Alison Peat, Maqsood Manzur, Umesh Chauhan, Lesley Miller, Katie O’Connell-Binns, Simon Wetherell, Sam Moon, Sarah Bland, Julia Leach, Mark Sloan, Christine Shepherd, Ross Dyer-Smith, Maarten Derks, Karen Read, Jodie Button, Martin Hadley-Brown, Sandra Smith, Caroline Hutson, Barbara Stewart, Karen Norcott, Andrew Slattery, Davinder Singh, Rose Fells, Susie Foster, Liz Tomlinson, Michaela Coutts, Kathryn Morgan, David Cowling, Joanna Beldon, Caite Guest, Bruce Helme, Daniel Tacagni, Nikki Davies, Angela Sanders, Paul Harris, Angela Juhasz, Anne Jenkins, Kirsteen Rakin, Tracey Hayward-Allingham, Samantha Kirby, Kumani Jeyarajah, Beata Guss, Dorota Daukszewicz, Yesim Ozcan, Jaisun Vivekanandaraja, Preeti Pandya, Stella Oldham, Lindsey Roberts, Julie Fuller, Murtaza Khanbhai, Jonathan Barnett, Veridiana Toledo, David Collier, Anne Zak, Rebecca James, Yasmin Choudhury, Mary Feely, Manish Saxena, Julian Shiel, Julia Colclough, Elizabeth Butterworth, Alison Crumbie, Jill Barlow, Nicola Jayne Pascall

**Affiliations:** 1 Department of Non-communicable Disease Epidemiology, London School of Hygiene and Tropical Medicine, London, UK; 2 Department of Medical Statistics, London School of Hygiene and Tropical Medicine, London, UK; 3 Clinical Trials Unit, Department of Population Health, London School of Hygiene and Tropical Medicine, London, UK; 4 Nuffield Department of Primary Care Health Sciences, University of Oxford, Oxford, UK; 5 Division of Informatics, Imaging and Data Sciences, University of Manchester, Manchester, UK; 6 MEMO Research, University of Dundee, Dundee, UK; 7 Medical Research Council Population Health Research Unit, Nuffield Department of Population Health, University of Oxford, Oxford, UK; 8 Bay Medical Practice, York Bridge Surgery, Morecambe, UK; 9 NIHR Clinical Research Network, Guy’s Hospital, London, UK; 10 Keats and Hampstead Group Practice, London, UK; 11 School of Primary Care and Population Sciences, University of Southampton, Southampton, UK; 12 Patient member of the trial steering committee appointed by NIHR, London, UK

## Abstract

**Objective:**

To establish the effect of statins on muscle symptoms in people who had previously reported muscle symptoms when taking statins.

**Design:**

Series of randomised, placebo controlled n-of-1 trials.

**Setting:**

Primary care across 50 sites in the United Kingdom, December 2016 to April 2018.

**Participants:**

200 participants who had recently stopped or were considering stopping treatment with statins because of muscle symptoms.

**Interventions:**

Participants were randomised to a sequence of six double blinded treatment periods (two months each) of atorvastatin 20 mg daily or placebo.

**Main outcome measures:**

At the end of each treatment period, participants rated their muscle symptoms on a visual analogue scale (0-10). The primary analysis compared symptom scores in the statin and placebo periods.

**Results:**

151 participants provided symptoms scores for at least one statin period and one placebo period and were included in the primary analysis. Overall, no difference in muscle symptom scores was found between the statin and placebo periods (mean difference statin minus placebo −0.11, 95% confidence interval −0.36 to 0.14; P=0.40)). Withdrawals because of intolerable muscle symptoms were 18 participants (9%) during a statin period and 13 (7%) during a placebo period. Two thirds of those completing the trial reported restarting long term treatment with statins.

**Conclusions:**

No overall effect of atorvastatin 20 mg on muscle symptoms compared with placebo was found in participants who had previously reported severe muscle symptoms when taking statins. Most people completing the trial intended to restart treatment with statins. N-of-1 trials can assess drug effects at the group level and guide individual treatment.

**Trial registration:**

ISRCTN30952488, EUDRACT 2016-000141-31, NCT02781064.

## Introduction

Statins reduce cardiovascular disease events in primary and secondary prevention, in men and women, and across all age groups.^1^ ^2^ Systematic reviews and meta-analyses of randomised trials have confirmed the safety of statins.^3^ Although severe adverse effects are rare, statins increase the risk of myopathy (absolute excess risk about 1 in 10 000 people treated annually), which can progress to severe rhabdomyolysis (about 0.2 in 10 000 people treated annually).^3^ Uncertainty persists about less severe muscle symptoms, however. Many people believe that statins frequently cause muscle pain,^4^ ^5^ ^6^ a view that has been reinforced by results from unblinded observational studies^6^ ^7^ and media reports.^8^ ^9^ ^10^ This belief has led to patients discontinuing treatment,^6^ ^11^ ^12^ exposing them to an increased risk of cardiovascular disease.^13^

For a patient in routine clinical care, reliably determining whether muscle symptoms are caused by statins is not easy for the clinician or patient.^14^ ^15^ ^16^ One way to deal with this uncertainty is to conduct blinded n-of-1 trials in individual patients with symptoms during treatment with statins. N-of-1 trials are a randomised trial in individual patients^17^ that can provide information to help determine the best course of action in an individual. When a number of n-of-1 individuals are combined in an analysis, the result can also be used to assess the overall effect of a treatment. 

We describe the results of StatinWISE (Statin Web-based Investigation of Side Effects), a series of n-of-1 trials comparing treatment periods of statins and placebo in people who had previously reported muscle symptoms when taking statins. Our aim was to establish the effect of statins on all muscle symptoms and on muscle symptoms that are perceived to be related to statins.

## Methods

### Trial design

StatinWISE was a series of randomised, double blind, placebo controlled n-of-1 trials. The overall length of the trial was one year for each participant and comprised six two month treatment periods (three of placebo, three of active treatment) in a randomly allocated order. 

### Participants

Participants were recruited from general practices in England and Wales and were considering stopping their statin (recruited opportunistically when they complained of symptoms during a consultation) or had stopped taking a statin in the last three years because of muscle symptoms (eligible patients were identified through a search of the medical records and invited by letter to a screening visit; more details in appendix 3). Participants were taking any type of statin at any dose before they were enrolled in the trial. Informed consent was given by each participant.

We excluded participants with previously raised levels of serum alanine aminotransferase (≥3 times the upper limit of normal), with persistent, generalised, unexplained muscle pain (whether or not associated with the use of statins) and levels of creatine kinase five times or more the upper limit of normal, any contraindications to atorvastatin 20 mg, or who the general practitioner considered unsuitable to participate in the trial.

### Sample size

We planned to recruit 200 participants to provide about 90% power to detect a treatment effect of at least one full unit on the visual analogue scale, assuming a type I error of 5% and allowing for loss to follow-up of 40% of participants (see sample size calculation in appendix 1).

### Randomisation and masking

Randomisation codes were generated and held securely by an information technology team and sponsor representative at the London School of Hygiene and Tropical Medicine Clinical Trials Unit, who were independent of the StatinWISE trial management team. The codes were made available to Sharp Clinical Services (UK), a good manufacturing practice certified clinical trial supply company, for the treatment packs to be manufactured according to the randomisation list.

Participants were allocated with equal probability to one of eight possible sequences (appendix fig 1), which ensured that all participants received one period of statins and one period of placebo in their first two treatment periods (in random order) and no one was allocated to three sequential periods of the same treatment. Randomisation codes were generated and held securely and confidentially by an information technology team and sponsor representative at the London School of Hygiene and Tropical Medicine Clinical Trials Unit who were independent of the StatinWISE trial management team and the general practitioner surgery staff, ensuring that the allocations were concealed. A physical copy of the randomisation codes was stored in a sealed and signed envelope in the locked office of the director of the clinical trials unit. The codes were made available to Sharp Clinical Services (UK) (marketing authorisation No 10284). Placebo was manufactured and packaged by Sharp Clinical Services (UK) Ltd to match atorvastatin. More information on drug manufacture is available in the protocol (appendix 3).

### Interventions and outcomes

Daily atorvastatin (20 mg) was compared with matching placebo over six two month treatment periods. The primary outcome was self-reported muscle symptoms, defined as pain, weakness, tenderness, stiffness, or cramp of any intensity. The primary outcome was measured each day with a validated visual analogue scale (0-10, score 0=no symptoms, 5=moderate symptoms, and 10=worst possible symptoms)^18^ for the last seven days of each treatment period (appendix 1 has more detail on data collection tools). We aimed to collect symptoms with a web based database or mobile app but our patient representatives recommended that participants should also be allowed to submit their scores over the telephone or by paper questionnaire. Participants reporting by telephone were asked to score their symptoms on an analogue severity scale, from 0 to 100 (with scores divided by 10 to match the visual scale), and did not use a visual scale. Measuring symptoms only during the last week of each two month treatment period was designed to avoid any carryover effect.

A secondary outcome was collected three months after the end of the final treatment period: we determined whether the participant had, or intended to, restart treatment with statins, and asked participants whether they had found their own trial result helpful in making the decision about their future use of statins. Other prespecified secondary outcomes (described in the protocol, appendix 3) were collected on the last day of each two month treatment period by questionnaire. These included binary measures for experience of muscle symptoms and if the symptoms were attributed to the study drug treatment, site of muscle symptoms, visual analogue scale scores (0-10) for the effect of their muscle symptoms on general activity, mood, ability to walk, normal work, relationships with other people, sleep, and enjoyment of life, and any other symptoms that the participant attributed to the study drug treatment. The questions related to symptoms experienced during the whole treatment period. Adherence to the study drug treatment was self-reported and verified by a drug accountability count of returned packs of drugs.

## Statistical methods

### Individual n-of-1 trials

At the end of each n-of-1 trial (after period 6, or at withdrawal), participants received numerical and graphical summaries of their individual data, in relation to their statin and placebo periods (appendix 4) and were invited to discuss these with their general practitioner, who also received a copy. The n-of-1 trial methodology allows for the use of the personalised results document. Participants were then asked if the personalised results document was helpful and whether they would restart treatment with statins.

### Combined analysis of n-of-1 trials

To estimate the overall effect of the trial treatment on muscle symptom scores, data from each n-of-1 trial were aggregated. The primary analysis included all participants who entered data on muscle symptoms at least once during a treatment period with statins and at least once during a treatment period with placebo. Statistical information about the treatment effect is limited if participants enter data only under one condition because the mixed models used in our primary analysis rely on within participant information. The primary analysis was a linear mixed model for visual analogue scale muscle symptom scores with random effects for participant and treatment. The analysis accounted for correlation between the seven daily measurements by modelling the residual errors with a first order autoregressive error structure within each treatment period, and non-normality of the symptom scores by robust standard errors. For the primary outcome, 95% confidence intervals are presented with a two sided P value. For secondary outcomes, 99% confidence intervals are presented to account for multiple testing.

Period effects were explored in sensitivity analyses. To assess differences between data collection methods, the primary analysis was repeated adjusting for the data collection method and allowing the treatment effect and the residual variance to vary by the data collection method.

### Secondary analyses

The binary measure of whether the participant reported having or not having muscle symptoms during that treatment period (with participants contributing one response per period until completion or withdrawal) was analysed with a logistic mixed model with random participant and treatment effects. This binary measure was then combined with the follow-up question about attribution, to obtain one binary measure of whether the participant reported having muscle symptoms that could not be attributed to another cause (eg, strenuous exercise). This binary measure was analysed with a similar logistic mixed model.

Secondary outcomes of the effect of the statin on other aspects of life were analysed similarly to the primary outcome, omitting the autoregressive correlation structure. We recorded the number and proportion of participants who decided to continue to use statins three months after their treatment ended (month 15). Symptom scores during treatment with statins and placebo were summarised according to a participant’s decision about whether to continue to use statins at month 15.

We used graphical and descriptive summaries to explore how withdrawals and adherence related to the statin and placebo periods. In patients who had not withdrawn before the start of the trial, a multinomial model was used to compare the probabilities of participants withdrawing during a placebo period, withdrawing during a statin period, or completing the trial. Analyses were repeated restricting to withdrawals because of intolerable symptoms. Risk ratios, P values, and 95% confidence intervals were calculated. All analyses were prespecified. A data monitoring committee oversaw the study. The trial was registered on ISRCTN registry (ISRCTN30952488), the European Union Drug Regulating Authorities Clinical Trials Database (EUDRACT 2016-000141-31), and on Clinicaltrials.gov (NCT02781064).

### Patient and public involvement

A StatinWISE patient involvement group was involved in trial design, specifically the packing and distribution of the drug, design of the data collection tools, and the content and wording of patient documents. The group was involved in trial conduct through membership in the Trial Steering Committee and provided substantial input into the individual participants’ results feedback document. Patient representatives provided active input into the interpretation and presentation of the results.

## Results

### Recruitment, participant flow, and baseline characteristics

We recruited 200 participants between 20 December 2016 and 5 April 2018, and the last participant follow-up was on 5 July 2019. Mean age was 69.1 (standard deviation 9.5), 115/200 (58%) participants were men, and 140/200 (70%) had a history of cardiovascular disease. Median total cholesterol concentration was 5.3 mmol/L (interquartile range 4.4 to 6.2) (table 1).

**Table 1:** Baseline characteristics

Characteristic	Frequency (%)*
Total No of randomised participants	200 (100)
Age (mean, SD)	69.1 (9.5)
Age	
35-49	7 (3.5)
50-64	49 (24.5)
65-79	115 (57.5)
≥80	29 (14.5)
Sex	
Women	85 (42.5)
Men	115 (57.5)
Ethnicity	
Asian	11 (5.5)
Black	8 (4)
Other	2 (1)
White	179 (89.5)
Smoking status	
Current smoker	14 (7)
Ex-smoker	105 (52.5)
Non-smoker	81 (40.5)
Diabetes	
No	167 (83.5)
Yes	33 (16.5)
Cardiovascular disease history	
No	60 (30)
Yes	140 (70)
Cholesterol (mmol/L; median (IQR))†	5.3 (4.4-6.2)
QRISK2 score, for participants with no history of cardiovascular disease (median (IQR))	18.3 (9.6-28.8)
Statin status at recruitment	
Stopped	151 (75.5)
Considering stopping	49 (24.5)

IQR=interquartile range; SD=standard deviation.

* Unless otherwise indicated.

† One value missing; values taken within the three years preceding recruitment, and therefore some patients will have been taking statins and others not.

### Numbers analysed

**Fig 1:**
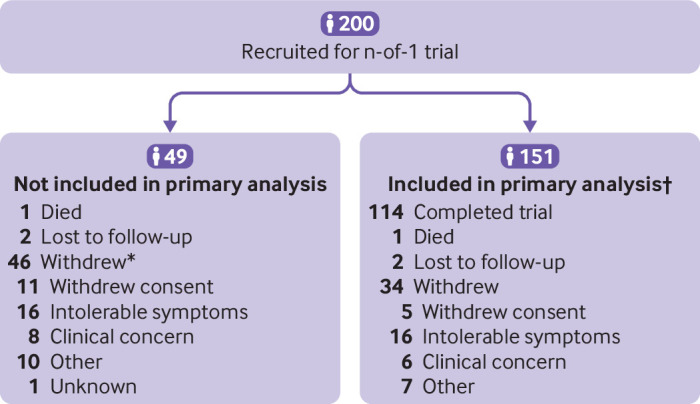
Recruitment and participant flow. *Seven of these 46 participants never received treatment (one withdrew consent, one had intolerable symptoms, one had clinical concerns, and four for other reasons). †152 participants were included in the secondary analysis (one participant completed the patient questionnaire in two periods but the primary outcome in only one)

Of the 200 participants, 151 (76%) provided one or more visual analogue scale measurements in both a statin period and a placebo period and were included in the primary analysis (fig 1); 86/200 (43%) participants did not complete the whole trial (two died, four were lost to follow-up, and 80 withdrew). The 151 participants included in the primary analysis contributed 2638 measurements during 392 statin periods and 2576 symptom score measurements during 383 placebo periods. Each of these measurements contributed to the primary analysis. The mean number of scores per participant was 34.5 (range 8-42). Appendix figure 3 shows the distribution of symptom scores across all periods. In period 1, 164/200 (82%) participants provided at least one daily report of muscle symptoms on the visual analogue scale, decreasing to 75% in period 2 (n=149/200) and to 58% (n=115/200) in period 6. Most (181/200, 91%) participants provided outcome data online or by paper (appendix table 1).

### Primary outcome

The observed mean muscle symptom score on the visual analogue scale was lower during statin treatment periods (mean 1.68, standard deviation 2.57) than during placebo periods (1.85, 2.74). We found no differences in mean muscle symptom scores between the statin and placebo periods (mean difference statin minus placebo −0.11 (95% confidence interval −0.36 to 0.14); P=0.40). Participants contributed different numbers of periods to the analysis and so the estimated treatment effect was not identical to the crude difference in means. We found no evidence that the effect of statins on the primary outcome was modified by the method of data collection (appendix table 2).

### Secondary outcomes

We found no evidence of an effect of statins on the occurrence of muscle symptoms overall (odds ratio 1.11; 99% confidence interval 0.62 to 1.99) or for muscle symptoms that could not be attributed to another cause (1.22; 0.77 to 1.94) among the 152 participants who contributed at least one secondary outcome measurement in a statin period and one in a placebo period (one additional participant provided secondary outcome data through our questionnaire than provided primary outcome data (table 2). For the other secondary outcomes (general activity, mood, ability to walk, normal work, relationships with other people, sleep, and enjoyment of life), we found no differences in symptom scores measured on the visual analogue scale between the statin and placebo periods (table 3). Site of symptoms was reported in 481/493 (97.6%) reports of muscle symptoms; most (312/481, 64.9%) were in the lower limbs (appendix table 3).

**Table 2:** Estimated effects for secondary outcomes comparing statin with placebo periods (from participant questionnaire; n=152)

	No (%) of participants		Odds ratio (99% CI)
	Statin periods	Placebo periods	
Muscle symptoms	248/397 (62.5)	239/388 (61.6)	1.11 (0.62 to 1.99)
Muscle symptoms, not attributed to other causes	216/397 (54.4)	200/388 (51.6)	1.22 (0.77 to 1.94)

Participants contributed multiple periods to these summaries and so the odds ratio cannot be directly calculated from these fractions. Odds ratios above 1 indicate higher odds on statins.

**Table 3:** Estimated effects for secondary outcomes comparing statin and placebo periods (from participant questionnaire) for aspects of daily life (n=152)

	Mean difference, cm (99% CI)
General activity	0.09 (−0.25 to 0.42)
Mood	0.26 (−0.04 to 0.56)
Ability to walk	0.11 (−0.22 to 0.43)
Normal work	0.15 (−0.17 to 0.46)
Relationships with other people	0.15 (−0.09 to 0.39)
Sleep	−0.02 (−0.32 to 0.29)
Enjoyment of life	0.13 (−0.22 to 0.48)

Each analysis includes all participants with at least one measurement for that outcome during a placebo period and at least one measurement during a statin period. One additional participant provided secondary outcome data through our questionnaire than provided primary outcome data. Values greater than zero indicate more symptoms on statins.

### Participant decisions about ongoing use of statins

Of the 114 participants who completed six treatment periods, 113 (57% of 200 randomised participants) received their results during an end of trial discussion. One participant did not attend. Of these 113 participants, 99/113 (88%) said that the trial had been helpful and 74/113 (66%) said that they had already or intended to resume taking statins. Of the 113 participants, 17 (15%) had a mean muscle symptom score at least one unit higher during the statin than the placebo periods and had been informed that statins might be contributing to their muscle symptoms. Among these 17 patients, nine (53%) said they planned to restart treatment with statins. Of the remaining 96 participants who had been informed that statins were unlikely to be contributing to their muscle symptoms, 65 (68%) said they planned to restart treatment with statins.

### Withdrawals

Table 4 shows the reasons for withdrawal. Of the 80 withdrawals, 34/80 (43%) occurred during a statin period, 39/80 (49%) during a placebo period, and 7/80 (9%) after randomisation but before the study drug was taken. Overall, few participants withdrew because of muscle symptoms. During statin periods, 18/200 (9%) participants withdrew because of intolerable symptoms compared with 13/200 (7%) during placebo periods. Appendix figure 4 shows the mean symptom scores for those who withdrew versus those that did not withdraw. Among the 193 participants who had not withdrawn before the start of the trial, our multinomial models showed no evidence of a difference in the probability of withdrawals during a statin period compared with a placebo period, either overall (risk ratio 0.87, 95% confidence interval 0.55 to 1.38; P=0.56) or because of intolerable muscle symptoms (1.38, 0.66 to 2.83; P=0.56).

**Table 4:** Reasons for withdrawals by treatment

	All withdrawals (n=80)	During a placebo period (n=39)	During a statin period (n=34)	Neither (n=7)
Withdrawal of consent	16 (8)	8 (4)	7 (3.5)	1 (0.5)
Muscle symptoms*	1 (0.5)	1 (0.5)	0	0
Non-muscle symptoms*	4 (2)	2 (1)	2 (1)	0
Not related to drug treatment*	3 (1.5)	1 (0.5)	2 (1)	0
Switched to statin*	0	0	0	0
No reason given*	8 (4)	4 (2)	3 (1.5)	1 (0.5)
Intolerable muscle symptoms	32 (16)	13 (6.5)	18 (9)	1 (0.5)
Clinical concern	14 (7)	9 (4.5)	4 (2)	1 (0.5)
Muscle symptoms*	1 (0.5)	1 (0.5)	0	0
Non-muscle symptoms*	4 (2)	2 (1)	2 (1)	0
Not related to drug treatment*	5 (2.5)	3 (1.5)	2 (1)	0
Switched to statin*	4 (2)	3 (1.5)	0 (0)	1 (0.5)
Other	17 (8.5)	8 (4)	5 (2.5)	4 (2)
Non-muscle symptoms*	4 (2)	4 (2)	0	0
Not related to drug treatment*	13 (6.5)	4 (2)	5 (2.5)	4 (2)
Unknown	1 (0.5)	1 (0.5)	0	0

Data are number (%) of participants from 200 participants randomised to a sequence of treatments.

* Post hoc finer categorisation.

### Adherence

Adherence reported by participants was confirmed by verification of the number of pills remaining in the returned drug treatment packs. Adherence to the study drug treatment was high, with at least 80% of participants reporting taking their drug treatment “every day” or “most days” during each period, for participants who had not yet withdrawn (appendix table 4 and appendix fig 2).

### Adverse events

During the trials, 13 serious adverse events were recorded; none was considered attributable to the study drug treatment. Two fatal events (one during statin treatment and one after the end of treatment) and 11 non-fatal events (five during statin treatment and six during placebo) were found.

## Discussion

### Principal findings

This series of n-of-1 trials recruited participants who were considering stopping or had stopped their statin treatment because of muscle symptoms. We found no differences in the frequency or severity of muscle symptoms between the statin and control periods. Also, we found no differences for the effect of muscle symptoms on aspects of daily life (general activity, mood, ability to walk, normal work, relationships with other people, sleep, and enjoyment of life) between the statin and control periods. Missing outcome data were equally distributed between the statin and placebo periods, making it unlikely that muscle symptoms contributed to missed outcome data collection. Of those completing the trial, most (88%) said that their n-of-1 trial had been helpful, with nearly two thirds reporting that they intended to restart treatment with statins.

We found no evidence of a difference in withdrawals between the statin and placebo periods but StatinWISE was not powered to detect a difference in withdrawals between periods, and our estimates did not exclude a difference. This highly selected population of participants had identified themselves at the start of the study as experiencing symptoms when taking statins that were severe enough to stop treatment. Withdrawal because of intolerable symptoms, however, was uncommon, and the excess comparing statins and placebo was only 2%.

### Comparison with other literature

StatinWISE and the concurrent SAMSON trial^19^ are the first large series of n-of-1 trials to investigate the effect of statins on muscle symptoms. Our findings support the limited evidence from one small n-of-1 trial and large systematic reviews and meta-analyses of randomised trials that have not established a clear effect of statins on muscle symptoms in the absence of myopathy.^2^ ^3^ ^20^ ^21^ Our data agree with the findings of the ODYSSEY ALTERNATIVE trial^22^ and the GAUSS-3 trial,^23^ which found that only a small proportion of patients intolerant to statins developed intolerable muscle symptoms when taking statins compared with placebo. Our data also agree with findings from a smaller cohort of patients with idiopathic inflammatory myopathies whose myalgia was not aggravated by statins.^24^ An ongoing meta-analysis^25^ is investigating data on adverse events from blinded, randomised trials of statins. Our findings clearly indicated that most patients taking statins did not experience symptoms causally related to their statin, highlighting the importance of blinding when assessing adverse effects.

Observational studies have reported adverse effects on muscle,^26^ and the experience of muscle symptoms when taking statins in clinical practice causes patients to stop treatment.^6^ Various explanations have been offered: the nocebo effect, in which expectations of adverse effects might lead patients to attribute muscle symptoms during treatment with statins to the statins themselves.^27^ Also, muscle aches and pains are common among the age group taking statins and might occur coincidentally with the use of statins, leading patients and clinicians to erroneously attribute pain to statins.^28^ Lack of randomisation and blinding in observational studies imply that for a subjective symptom, such as muscle pain, an association with the use of statins might not be causal. The large proportion of our participants who intended to restart treatment with statins after their trial is in line with observational data showing that rechallenge with statins can be tolerated by most patients.^29^ ^30^

### Strengths and weaknesses of the study

A common criticism of large placebo controlled trials of statins is that patients most likely to experience side effects are not included. StatinWISE included only patients who had experienced symptoms during treatment with statins. Also, in some larger trials, participants were not asked specifically about muscle symptoms and their intensity; in StatinWISE, patients were asked directly about the intensity of their muscle symptoms. We minimised bias and confounding by collecting data on muscle symptoms in a series of double blind trials, with randomised statin and placebo treatments.

Within subject designs tend to have greater statistical power, which was increased by repeated measurements in each treatment period, allowing us to investigate differences between statins and placebo with greater precision. The design also allowed us to feed back information to participants about whether their muscle symptoms occurred more frequently during the statin or placebo period, so that they could decide whether to continue treatment with statins.

In conducting this series of trials, we allowed participants to determine whether their symptoms were likely to be caused by statins. In this real world, general practice setting, we have shown the potential of these studies to be used in everyday clinical practice. The n-of-1 trial could be adopted by clinicians who are looking to establish the best course of treatment for patients, in general practice or outpatient settings, who present with muscle symptoms associated with statins.

Of the 200 randomised participants, 86 did not complete the whole trial, of whom 49 did not provide sufficient data to contribute to the primary analysis. Adherence was similar for the statin and placebo periods, and the trial was adequately powered to account for this level of attrition. We did not measure levels of creatine kinase in participants who withdrew from the study so we do not know what proportion of participants had biochemical evidence of muscle effects. For simplicity, we assessed the effect of one statin, atorvastatin 20 mg, on muscle symptoms. Our results, therefore, might not apply to higher doses of atorvastatin or to other statins. Although we intended to collect outcomes with web based methodology, over half of the participants preferred to report their symptoms on paper or by telephone. Our two month treatment periods were designed to be long enough to allow the previous treatment to washout, and to allow the current treatment to have an effect. It is possible, however, that this time period was not long enough for some of our patients, and that the scores on the visual analogue scale were affected by treatment from the previous period.

### Interpretation and future research

The analysis of our series of n-of-1 trials found no overall effect of statins on muscle symptoms in participants selected on the basis of having experienced severe muscle symptoms but no important increases in levels of enzymes during previous treatment with statins. The lack of effect in patients completing the trial, combined with the low number of withdrawals owing to muscle symptoms, suggests a nocebo effect among users of statins, or of high tolerance to blinded rechallenge.

Treatment with statins for those at high risk has potential health benefits that are lost by those who stop treatment. The availability of n-of-1 trial packs in clinical care would allow patients and clinician to replicate this study in individuals, for any statin and at any dose to suit clinical needs, in primary care or in lipid clinics. Our results suggest that most patients would restart treatment after such a trial. Future work could focus on conducting n-of-1 trials for other types of statins and higher doses, and for other drugs which are associated with transient adverse effects.

**What is already known on this topic**

A causal link between statins and rare but severe muscle adverse effects is well characterised but the causal effect of statins on less severe muscle symptoms, such as stiffness, pain, and weakness, is uncertain
Widely publicised results of unblinded observational studies has led to many patients stopping treatment, believing their muscle symptoms are caused by statins, thus increasing morbidity and mortality from cardiovascular disease
Blinded, randomised n-of-1 trials can provide evidence of the role of statins in muscle symptoms

**What this study adds**

In a series of randomised, placebo controlled n-of-1 trials, no overall effect of statins on the frequency or severity of muscle symptoms was found in participants who had previously reported severe muscle symptoms when taking statins
Most people completing the trial planned to restart long term treatment with statins
The n-of-1 trial could be a powerful clinical tool for clinicians and patients to determine how best to investigate muscle symptoms associated with statins

## Web extra

material supplied by authors

Web appendix 1: Supplementary material

Web appendix 2: StatinWISE Trial Group members

Web appendix 3: StatinWISE protocol

Web appendix 4: Personalised results document

## Notes

Contributors: EH designed the study, oversaw the study conduct, and wrote the first draft of the manuscript with LS. EW designed the study, analysed the results, and reviewed the manuscript. KB and DB designed and managed the study and reviewed the manuscript. AP managed the study and reviewed the manuscript. AT coordinated the data collection and reviewed the manuscript. HS-S and IR designed the study, oversaw its conduct as co-directors of the Clinical Trials Unit, and reviewed the manuscript. DP designed the study, managed the study data, and reviewed the manuscript. BG, TvS, TMM, and JA designed the study and reviewed the manuscript. JW was PI at the top recruiting site and reviewed the manuscript. PM was lead research nurse at the top recruiting site and reviewed the manuscript. JS was lead research nurse at the top recruiting network of sites and reviewed the manuscript. LB was research co-ordinator at the first site to recruit and reviewed the manuscript. MM provided oversight of the whole study and reviewed the manuscript. MH contributed to study design and conduct as a patient representative and reviewed the manuscript. LS designed and led the study and wrote the first draft of the manuscript with EH. LS is the guarantor for the study. The corresponding author attests that all listed authors meet authorship criteria and that no others meeting the criteria have been omitted.

Ethical approval: The trial was approved by South Central-Hampshire A Research Ethics Committee (16/SC/0324) and the Health Research Authority. Regulatory approval (17072/0009/001-0001) was from the Medicines and Healthcare products Regulatory Authority. The approved protocol is available from https://statinwise.lshtm.ac.uk/live/gp/.

Data sharing: Deidentified, patient level trial data will be shared along with the protocol, statistical analysis plan, and analytic code, from the LSHTM Data Compass Repository (https://datacompass.lshtm.ac.uk/1314/). Data will be available from the time of publication with no set end date.

The corresponding author, LS, affirms that the manuscript is an honest, accurate, and transparent account of the study being report; that no important aspects of the study have been omitted; and that any discrepancies from the study as originally planned and registered have been explained.

Dissemination to participants and related patient and public communities: Each participant’s own trial results were presented to them at the end of their trial. The overall results of the trial will be disseminated to trial collaborators, the media, on the trial website (https://statinwise.lshtm.ac.uk/live), and to relevant patient organisations. A plain English summary is available in the supplementary material (appendix 1).

Provenance and peer review: Not commissioned; externally peer reviewed.
