# Supplementary material for: Statin treatment and muscle symptoms: series of randomised, placebo controlled n-of-1 trials
Source: BMJ. 2021 Feb 24;372:n135. doi: 10.1136/bmj.n135 (PMC7903384; doi:10.1136/bmj.n135)
Supplement: Supplementary file 1 — Web appendix 1: Supplementary material [file here059829.ww1.pdf]

Supplementary material

Table of Contents

Supplementary Information 2

Sample size calculation 2

Supplementary Tables 3

Appendix Table 1. Data collection method chosen 3

Appendix Table 2. Estimated treatment effect from a linear mixed model allowing the statin effect to vary by data collection method chosen 3

Appendix Table 3 Location of muscle symptoms reported 3

Appendix Table 4. Self-reported adherence to study medication by period 4

Supplementary Figures 5

Appendix Figure 1. Treatment sequences in StatinWISE (S=statin, P=placebo) 5

Appendix Figure 2 Adherence to study medication by period and sequence, for all recruited participants (left) and those who had not yet withdrawn (right) 6

Appendix Figure 3 Distribution of visual analogue scale scores (0-10) across all trial periods. 6

Appendix Figure 4 Mean visual analogue scales of withdrawers and non-withdrawers in each period, stratified by treatment with statin or placebo. 7

CONSORT extension for N-of-1 trials 8

Lay summary 12

References 12

Supplementary Information

Sample size calculation

The power calculations are based on being able to detect a 1cm difference in the VAS pain score. This was chosen to represent the smallest VAS change in pain which patients would perceive as being beneficial, and might therefore change a patient's decision regarding subsequent statin use. Two studies have concluded that the smallest change in VAS pain score corresponding to "a little more" or "a little less" pain was 1.3cm, with a lower limit of the confidence interval at 1cm.[1,2] A 1.3cm minimum change value was used to power a pilot series of N-of-1 trials for statin adverse effects,[3] and our study is therefore powered for a 1cm change as a conservative estimate of the smallest beneficial change.

Using simulation, we estimated that a sample size of 64 participants provides approximately 90% power to detect a treatment effect of at least 1cm, assuming a Type I error of 5%. Allowing for loss to follow-up of 40% of participants through the trial inflates the required sample size to 107 participants.

Period effects (changes in underlying VAS pain score due to factors other than randomised treatment, e.g. seasonal, activity-related, etc.), variability in individual statin effects across participants, and imperfect adherence to the assigned treatment were investigated by further detailed simulations. VAS pain scores are not normally distributed, since they are bounded (0-10cm) and can display large fluctuations in response. Therefore, further power calculations were performed drawing the outcome from a Beta distribution, and from a distribution with normal variance components on a logit scale, to assess the robustness of the sample size estimates to the distribution chosen. These factors all have the effect of decreasing power, thus increasing the sample size required. An approximate 80% increase in the sample size required in the absence of these effects provided approximately 90% or more power across a plausible range of these potential effects, thus we determined that a final sample size of 200 was required.

Supplementary Tables

Appendix Table 1. Data collection method chosen

		All
Randomised		
	Total	200 (100)
Collection method		
Mobile app		2 (1.0)
Online		88 (44.0)
Paper		93 (46.5)
Phone		17 (8.5)

Appendix Table 2. Estimated treatment effect from a linear mixed model allowing the statin effect to vary by data collection method chosen

	Estimated mean difference	95% CI	p-value*
**Statin effect among people reporting outcomes online**	0.008	(-0.358, 0.373)	0.968
**Difference in statin effects for people reporting using the following methods:**			0.594
**App**	-0.065	(-0.704, 0.574)	
**Paper**	-0.159	(-0.676, 0.358)	
**Phone**	-0.681	(-1.680, 0.318)	
**Collection method**			
**Online**	Reference		
**App**	0.563	(-0.223, 1.349)	0.161
**Paper**	0.604	(-0.081, 1.289)	0.084
**Phone**	1.949	(0.352, 3.545)	0.017
**Constant**	1.441	(1.053, 1.829)	<0.001

* p-value for interaction: joint test for three interaction parameters (i.e. testing the null hypothesis that statin effect does not vary by data collection method).

Appendix Table 3 Location of muscle symptoms reported

	Frequency (n=481)	% of reports
Head and Neck	18	3.7
Lower limbs	312	64.9
Trunk	73	15.2
Upper limbs	78	16.2

Appendix Table 4. Self-reported adherence to study medication by period

	Period	All	Sequence 1 PSPSPS	Sequence 2 PSPSSP	Sequence 3 PSSPPS	Sequence 4 PSSPSP	Sequence 5 SPPSPS	Sequence 6 SPPSSP	Sequence 7 SPSPPS	Sequence 8 SPSPSP
**Total**		200 (100)	18 (100)	24 (100)	29 (100)	26 (100)	22 (100)	22 (100)	27 (100)	32 (100)
**Reported[a] taking study drug every day or most days, n (%)**	1	157 (78.5)	13 (72.2)	19 (79.2)	21 (72.4)	20 (76.9)	20 (90.9)	16 (72.7)	22 (81.5)	26 (81.3)
	2	141 (70.5)	10 (55.6)	16 (66.7)	22 (75.9)	19 (73.1)	19 (86.4)	15 (68.2)	16 (59.3)	24 (75)
	3	120 (60)	7 (38.9)	15 (62.5)	21 (72.4)	14 (53.8)	14 (63.6)	13 (59.1)	14 (51.9)	22 (68.8)
	4	121 (60.5)	8 (44.4)	15 (62.5)	20 (69)	14 (53.8)	14 (63.6)	15 (68.2)	16 (59.3)	19 (59.4)
	5	110 (55)	7 (38.9)	13 (54.2)	18 (62.1)	12 (46.2)	14 (63.6)	12 (54.5)	15 (55.6)	19 (59.4)
	6	112 (56)	7 (38.9)	13 (54.2)	19 (65.5)	13 (50)	14 (63.6)	13 (59.1)	14 (51.9)	19 (59.4)
**Reported[b] taking study drug 5+ days in last week of period, n (%)**	1	152 (76)	13 (72.2)	18 (75)	21 (72.4)	20 (76.9)	19 (86.4)	14 (63.6)	21 (77.8)	26 (81.3)
	2	137 (68.5)	11 (61.1)	16 (66.7)	21 (72.4)	19 (73.1)	16 (72.7)	15 (68.2)	17 (63)	22 (68.8)
	3	117 (58.5)	7 (38.9)	15 (62.5)	19 (65.5)	14 (53.8)	14 (63.6)	11 (50)	15 (55.6)	22 (68.8)
	4	115 (57.5)	8 (44.4)	14 (58.3)	19 (65.5)	13 (50)	13 (59.1)	14 (63.6)	14 (51.9)	20 (62.5)
	5	107 (53.5)	7 (38.9)	13 (54.2)	16 (55.2)	13 (50)	12 (54.5)	13 (59.1)	14 (51.9)	19 (59.4)
	6	109 (54.5)	7 (38.9)	13 (54.2)	18 (62.1)	12 (46.2)	14 (63.6)	13 (59.1)	13 (48.1)	19 (59.4)
**Median (25th, 75th percentile) number days reported[b] taking study drug in last week**	1	7 (5,7)	7 (0,7)	7 (3.5,7)	7 (2,7)	7 (5,7)	7 (6,7)	6.5 (0,7)	7 (5,7)	7 (6.5,7)
	2	7 (0,7)	6.5 (0,7)	7 (0,7)	7 (4,7)	7 (0,7)	7 (4,7)	7 (0,7)	7 (0,7)	7 (1,7)
	3	7 (0,7)	0 (0,7)	7 (0,7)	7 (0,7)	6.5 (0,7)	7 (0,7)	4.5 (0,7)	5 (0,7)	7 (0,7)
	4	6.5 (0,7)	0 (0,7)	7 (0,7)	7 (0,7)	5 (0,7)	6 (0,7)	7 (0,7)	5 (0,7)	7 (0,7)
	5	6 (0,7)	0 (0,7)	6 (0,7)	7 (0,7)	2.5 (0,7)	7 (0,7)	7 (0,7)	5 (0,7)	6.5 (0,7)
	6	6 (0,7)	0 (0,7)	6 (0,7)	7 (0,7)	1 (0,7)	7 (0,7)	7 (0,7)	3 (0,7)	6.5 (0,7)

[a] in participant questionnaire (once per period); [b] in daily reports in last week of period.

Supplementary Figures

Allocated sequence over the six periods	Frequency (n=200)	%
PSPSPS	18	9.0
PSPSSP	24	12.0
PSSPPS	29	14.5
PSSPSP	26	13.0
SPPSPS	22	11.0
SPPSSP	22	11.0
SPSPPS	27	13.5
SPSPSP	32	16.0

Appendix Figure 1. Treatment sequences in StatinWISE (S=statin, P=placebo)

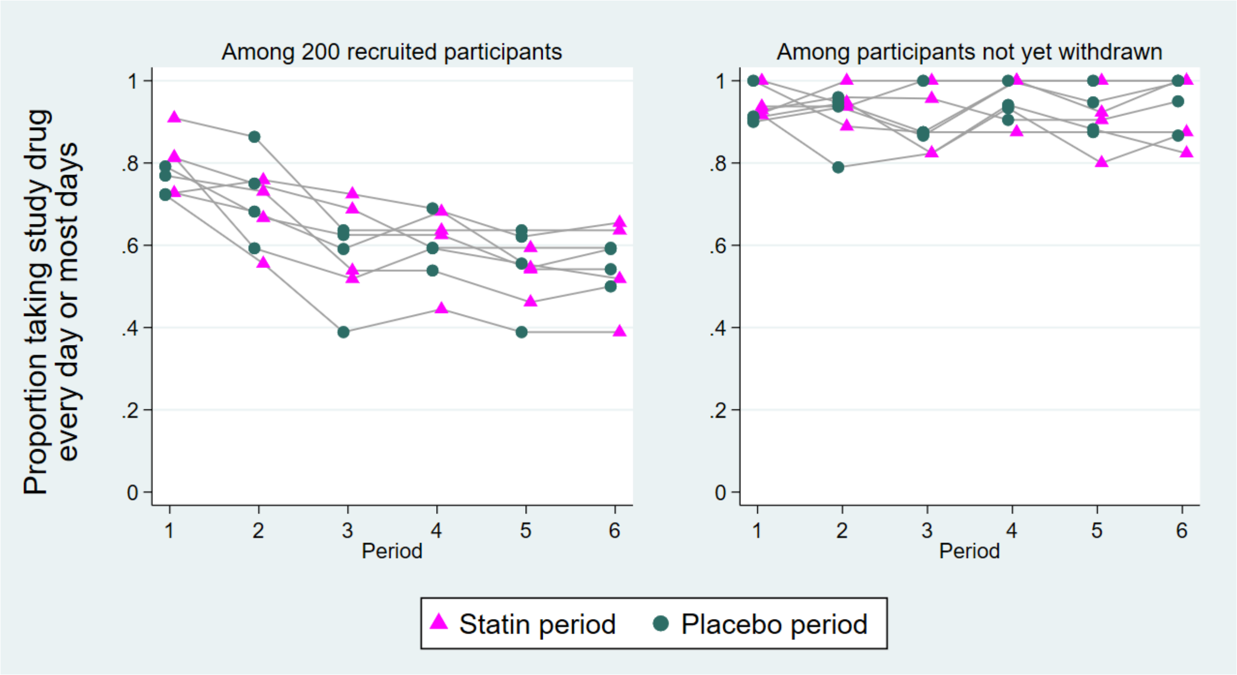

Appendix Figure 2 Adherence to study medication by period and sequence, for all recruited participants (left) and those who had not yet withdrawn (right)

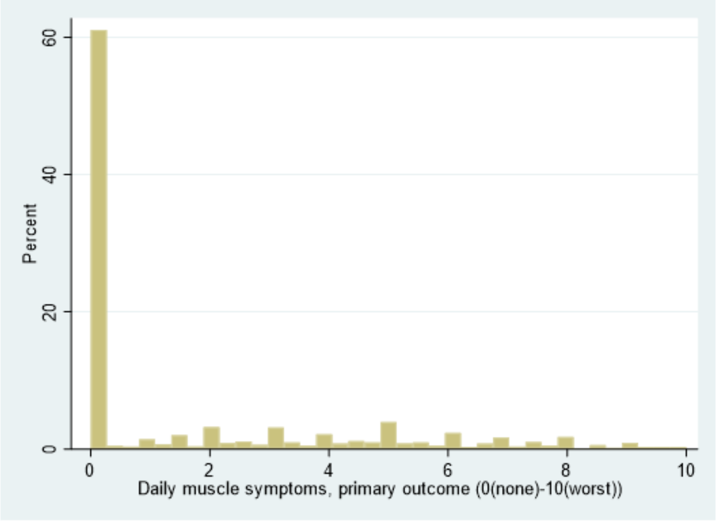

Appendix Figure 3 Distribution of visual analogue scale scores (0-10) across all trial periods.

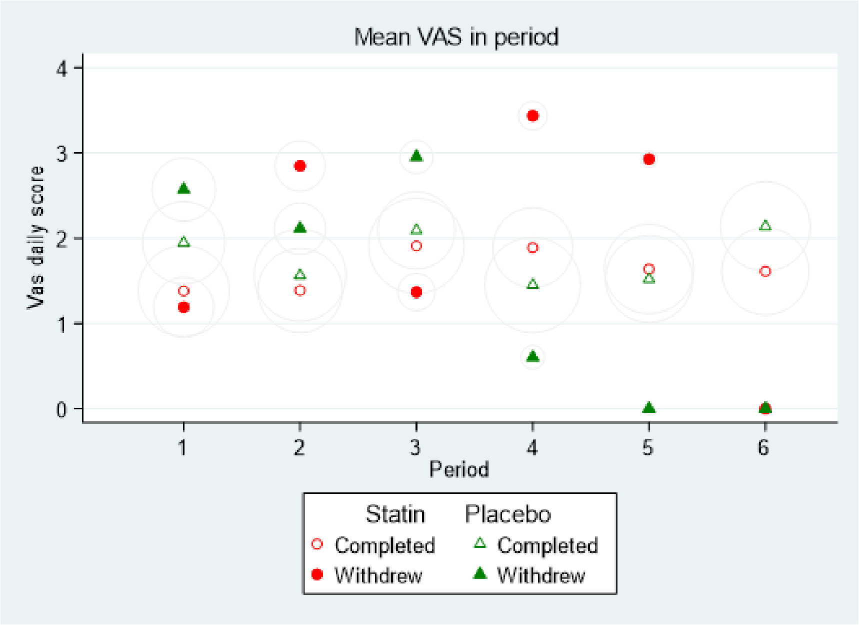

Appendix Figure 4 Mean visual analogue scales of withdrawers and non-withdrawers in each period, stratified by treatment with statin or placebo.

(Note: the size of the circle around each data point is proportional to the sample size used to calculate the mean)

CONSORT extension for N-of-1 trials

CENT 2015 checklist*; CONSORT 2010 checklist items with modifications or additions for individual or series of N-of-1 trials; empty items in the CENT 2015 column indicate no modification from the CONSORT 2010 item

*It is strongly recommended that this checklist be read in conjunction with the CENT 2015 Explanation and Elaboration[24] for important clarification on the items. The copyright for CENT (including checklist) is held by the CENT Group and is distributed under a Creative Commons Attribution (CC-BY 4.0) license.

†Caution should be taken when reporting potentially identifying information pertaining to CENT items 4a, 4b, 14a, and 15.

Section/	CONSORT 2010		Page	CENT 2015		Page
Topic	No	Item	number	No	Item	number
**Title and abstract**						
	1a	Identification as a randomised trial in the title	1	1a	Identify as an “N-of-1 trial” in the title<br>***For series:*** Identify as “a series of N-of-1 trials” in the title	1
	1b	Structured summary of trial design, methods, results, and conclusions (for specific guidance see CONSORT for abstracts)	3-4	1b	*For specific guidance, see CENT guidance for abstracts (table 2)*	*3-4*
**Introduction**			-			
Background and objectives	2a	Scientific background and explanation of rationale	5	2a.1		
				2a.2	Rationale for using N-of-1 approach	5
	2b	Specific objectives or hypotheses	5-6	2b		
**Methods**						
Trial design	3a	Description of trial design (such as parallel, factorial) including allocation ratio	6	3a	Describe trial design, planned number of periods, and duration of each period (including run-in and wash out, if applicable)<br>***In addition for series:*** Whether and how the design was individualized to each participant, and explain the series design	6
	3b	Important changes to methods after trial start (such as eligibility criteria), with reasons	n/a	3b		
Participant(s)	4a	Eligibility criteria for participants	7	4a†	Diagnosis or disorder, diagnostic criteria, comorbid conditions, and concurrent therapies.<br>***For series:*** Same as CONSORT item 4a	7
	4b	Settings and locations where the data were collected	7	4b†		
				4c	Whether the trial(s) represents a research study and if so, whether institutional ethics approval was obtained	6
Interventions	5	The interventions for each group with sufficient details to allow replication, including how and when they were actually administered	9	5	The interventions for each period with sufficient details to allow replication, including how and when they were actually administered	9

Outcomes	6a	Completely defined pre-specified primary and secondary outcome measures, including how and when they were assessed	9	6a.1		
				6a.2	Description and measurement properties (validity and reliability) of outcome assessment tools	9
	6b	Any changes to trial outcomes after the trial commenced, with reasons	n/a	6b		
Sample size	7a	How sample size was determined	9	7a		
	7b	When applicable, explanation of any interim analyses and stopping guidelines	n/a	7b		
Randomisation:						
Sequence generation	8a	Method used to generate the random allocation sequence	8	8a	Whether the order of treatment periods was randomised, with rationale, and method used to generate allocation sequence	8
	8b	Type of randomisation; details of any restriction (such as blocking and block size)	8	8b	When applicable, type of randomisation; details of any restrictions (such as pairs, blocking)	8
				8c	Full, intended sequence of periods	Appendix fig 1
Allocation concealment mechanism	9	Mechanism used to implement the random allocation sequence (such as sequentially numbered containers), describing any steps taken to conceal the sequence until interventions were assigned	8	9		
Implementation	10	Who generated the random allocation sequence, who enrolled participants, and who assigned participants to interventions	8	10		
Blinding	11a	If done, who was blinded after assignment to interventions (for example, participants, care providers, those assessing outcomes) and how	8	11a		
	11b	If relevant, description of the similarity of interventions	8	11b		
Statistical methods	12a	Statistical methods used to compare groups for primary and secondary outcomes	10	12a	Methods used to summarize data and compare interventions for primary and secondary outcomes	10
	12b	Methods for additional analyses, such as subgroup analyses and adjusted analyses	n/a	12b	***For series:*** If done, methods of quantitative synthesis of individual trial data, including subgroup analyses, adjusted analyses, and how heterogeneity between participants was assessed, (for specific guidance on reporting syntheses of multiple trials, please consult the PRISMA Statement)	***10***

				12c	Statistical methods used to account for carryover effect, period effects, and intra-subject correlation	10-11
**Results**						
Participant flow (a diagram is strongly recommended)	13a	For each group, the numbers of participants who were randomly assigned, received intended treatment, and were analysed for the primary outcome	13	13a.1	Number and sequence of periods completed, and any changes from original plan with reasons	13
				13a.2	***For series:*** The number of participants who were enrolled, assigned to interventions, and analysed for the primary outcome	***13***
	13b	For each group, losses and exclusions after randomisation, together with reasons	13 and Figure 1	13c	***For series:*** losses or exclusions of participants after treatment assignment, with reasons, and period in which this occurred, if applicable	***11 and Figure 1***
Recruitment	14a	Dates defining the periods of recruitment and follow-up	13	14a†		
	14b	Why the trial ended or was stopped	n/a	14b	Whether any periods were stopped early and/or whether trial was stopped early, with reason(s).	n/a
Baseline data	15	A table showing baseline demographic and clinical characteristics for each group	Table 1	15†		
Numbers analysed	16	For each group, number of participants (denominator) included in each analysis and whether the analysis was by original assigned groups	13	16	For each intervention, number of periods analysed. ***In addition for series:*** if quantitative synthesis was performed, number of trials for which data were synthesized	13
Outcomes and estimation	17a	For each primary and secondary outcome, results for each group, and the estimated effect size and its precision (such as 95% confidence interval)	14 and table 2	17a.1	For each primary and secondary outcome, results for each period; an accompanying figure displaying the trial data is recommended.	-
				17a.2	For each primary and secondary outcome, the estimated effect size and its precision (such as 95% confidence interval) ***In addition for series:*** if quantitative synthesis was performed, group estimates of effect and precision for each primary and secondary outcome	14 and Table 2
	17b	For binary outcomes, presentation of both absolute and relative effect sizes is recommended	n/a	17b		
Ancillary analyses	18	Results of any other analyses performed, including subgroup analyses and adjusted analyses, distinguishing pre-specified from exploratory	14-15	18	Results of any other analyses performed, including assessment of carryover effects, period effects, intra-subject correlation ***In addition for series:*** If done, results of subgroup or sensitivity analyses	n/a
Harms	19	All important harms or unintended effects in each group (for specific guidance see CONSORT for harms)	16	19	All harms or unintended effects for each intervention. *(for specific guidance see CONSORT for harms)*	16
**Discussion**						

Limitations	20	Trial limitations, addressing sources of potential bias, imprecision, and, if relevant, multiplicity of analyses	19	20
Generalisability	21	Generalisability (external validity, applicability) of the trial findings	20-21	21
Interpretation	22	Interpretation consistent with results, balancing benefits and harms, and considering other relevant evidence	21	22
**Other information**				
Registration	23	Registration number and name of trial registry	12	23
Protocol	24	Where the full trial protocol can be accessed, if available	6	24
Funding	25	Sources of funding and other support (such as supply of drugs), role of funders	33	25

Lay summary

Statins are one of the most commonly prescribed drugs in the United Kingdom. There is strong evidence that they are effective in safely reducing heart disease. However, there is some doubt about whether statins cause muscle pain, stiffness or weakness. This research has been done to understand the effect of statins on muscle symptoms.

To answer our question, we asked 200 volunteers from across England and Wales to participate in the study. Patients who joined the study had either recently stopped taking statins due to muscle symptoms, or were considering stopping due to muscle symptoms. Patients who participated were randomly assigned to a sequence of six, two-month treatment periods during which they received either statins or a placebo. Neither patients, nor their GP knew which tablet they were receiving. This helps to reduce bias in the data. At the end of each treatment period, patients were asked to report any muscle symptoms and any other symptoms that they experienced.

The key result of this work is that patients reported no difference, on average, in their muscle symptoms between periods when they were taking a statin and periods when they were taking a placebo. We also assessed impact on the patient's quality of life by looking at how statins effected the following areas: general activity, mood, walking ability, normal work, relations with other people, sleep, and enjoyment of life. As with muscle symptoms, there was no evidence of a difference between statin and placebo periods. The majority of patients who finished the trial decided to continue using statins after the trial. Future research should be done to assess different statin doses – notably the higher doses often used following a heart attack. In addition, further work is needed to see how the approach we used could be adopted into everyday clinical care.

References

[1] Todd, K.H. and J.P. Funk, The minimum clinically important difference in physician-assigned visual analog pain scores. Acad Emerg Med, 1996. 3(2): p. 142-6.

[2] Gallagher, E.J., M. Liebman, and P.E. Bijur, Prospective validation of clinically important changes in pain severity measured on a visual analog scale. Ann Emerg Med, 2001. 38(6): p. 633-8.

[3] Joy, T.R., et al., N-of-1 (single-patient) trials for statin-related myalgia. Ann Intern Med, 2014. 160(5): p. 301-10.
