# Supplementary material for: Statin treatment and muscle symptoms: series of randomised, placebo controlled n-of-1 trials
Source: BMJ. 2021 Feb 24;372:n135. doi: 10.1136/bmj.n135 (PMC7903384; doi:10.1136/bmj.n135)
Supplement: Supplementary file 2 — Web appendix 2: StatinWISE Trial Group members [file here059829.ww2.pdf]

StatinWISE Trial Group members

Trial Steering Committee

Michael Moore (chair), Maurice Hoffman, Rebecca Harmston, Brian MacKenna, David Symes, Haleema Shakur, Liam Smeeth

Data Monitoring Committee

John Norrie (chair), Nicholas Mills, Hannah Castro

Protocol Committee

Emily Herrett, Elizabeth Williamson, Danielle Beaumont, Danielle Prowse, Nabila Youssouf, Kieran Brack, Jane Armitage, Ben Goldacre, Thomas M MacDonald, Tjeerd van Staa, Ian Roberts, Haleema Shakur-Still, Liam Smeeth.

Trial Coordinating Team

Liam Smeeth (Chief Investigator), Elizabeth Williamson (Statistician), Alexander Perkins (Trial Manager), Andrew Thayne (Data Assistant), Haleema Shakur-Still (Clinical Trials Unit Co-Director), Ian Roberts (Clinical Trials Unit Co-Director), Danielle Prowse (Assistant Data Manager), Danielle Beaumont (Senior Trial Manager), Nabila Youssouf (Senior Trial Manager), Kieran Brack (Senior Trial Manager), Collette Barrow (Trial Administrator), Sergey Kostrov (IT Systems Officer), Hakim Miah (IT Manager)

Trial Sites and Investigators

Albion Street Practice

- Eve Thacker (PI)
- Jayshireen Singh
- Melissa Baldey

Bay Medical Group

- Jonathon Wimborne (PI)
- Paula Melrose

- Eleanor Sowerby
- Nicola Harding
- Gail Timcke
- Alison Macleod (previous PI)
- Karen Lomax

Beechtree Surgery
- Nigel Wells (PI)
- Emma Pierre
- Tom Baker
- Carla Bratten

Bentley Surgery
- Karen Forshaw (PI)
- Daniel Clark
- Selina Fox
- Rachel Hubbard
- DG Crichton
- N Alsindi
- Mandy Hayes
- Keith (Peter) Elliot

Bicester Health Centre
- Robin Fox (PI)
- Jane Stanford
- Emily Ackland
- George Strong
- Debbie Kelly

Brigstock & South Norwood Partnership
- Dev Malhotra (PI)
- Jayshireen Singh
- Dipti Gandhi
- Gillian Foster

Brownlow Health
- Diane Exley (PI)
- Dawn Brayford
- Theresa Nuttall

Clarence Medical Centre
- Clare Corbett (PI)

- Nicola Anderton
- Gwyn Hughes
- Sian Turner
- Sarah Roberts
- David Brown

Cleveleys Group Practice
- Susan Fairhead (PI)
- Karen Sutcliffe

Conisbrough Group Practice
- Mark Boon (PI)
- Paula Dirienzo
- Kay Ellor

Creffield Medical Centre
- Hasan Chowhan (PI)
- Amy Townrow
- Tracey Rowles

Exchange Surgery (The)
- Debbie Hipps (PI)
- Jayshireen Singh

Falkland Surgery
- Geoffrey Perry (PI)
- Amanda Ayers
- Rebecca Cooper
- Sara Harley
- Lesley Parsons
- Ann Selby
- Regan Hood

Freshney (Littlefields) Green Primary Care Centre
- Elizabeth Zoon (PI)
- Lucy Wraith
- Vicky Peterson
- Jackie Pretty
- Narinder Dhillon

Great Sutton Medical Centre
- John Wearne (PI)

- Sandra Moss
- Kate Maitland
- Catherine Edge
- Susan Brown

Hampstead Group Practice
- Stuart Mackay-Thomas (PI)
- Lucy Brooks

Hope Family Medical Centre
- Heather Pearson (PI)
- Ewan Deas (previous PI)
- Lesley Yelland
- Helen Jones

Hornsey Rise
- Stephen Rogers (PI)
- Ian Huckle

Hoveton & Wroxham Medical Centre
- Carsten Dernedde (PI)
- Caroline Mansfield
- Heather Leishman
- Jordan Howard
- Chris Wright

Hurley Clinic
- Mark Ashworth (PI)
- Satinder Kumar
- Catarina Guerreiro
- Jayshireen Singh

Jorvik Gillygate Practice
- David Hartley (PI)
- Sally Gordon
- Carolyn Forrest
- Andy Gibson
- Laura Howe
- John Whitwell

Keats Medical Practice
- Irwin Nazareth (PI)

- Letitia Coco-Bassey
- Lucy Brooks
- Kate Walters (previous PI)
- Jaqueline Mburu
- Crystal Chetwood

Kings Road Surgery
- Lynne Dowding (PI)
- Alison Williams
- Nikki Richards

Long Stratton Medical Partnership
- Mini Nelson (PI)
- Emma Chadwick
- Helen Mingaye

Mathukia's Surgery
- Mehul Mathukia (PI)
- Jacqueline Mburu
- Anna Swinburn
- Janeth Tomakin
- Valentina Valasevich
- Crystal Chetwood

Mattishall & Lenwade Surgeries
- Hywel Jones (PI)
- Joanne Bannister
- Emma Edwards

North House Surgery
- Morag McDowall (PI)
- Beverley Hall
- Helen Permain
- Nigel Peacock
- Carol Harrison
- Lorraine Parsons

Oak Lodge Medical Centre
- Chuin Kee (PI)
- Paula McLaren
- Jacqueline Mburu
- Crystal Chetwood

Oak Tree Surgery

- Sherard Le Maitre (PI)
- Helen Nash
- Stephanie Evans
- Rachel Evans

Paxton Green Group Practice

- Stephen Miller (PI)
- Pooja Agarwal
- Oliver Booth
- Victoria Mayhew
- Jayshireen Singh
- Alison Peat

Pendle View Medical Centre

- Maqsood Manzur (PI)
- Umesh Chauhan (previous PI)
- Lesley Miller
- Katie O’Connell-Binns

Queen Square Medical Practice

- Simon Wetherell (PI)
- Sam Moon
- Nicola Harding
- Eleanor Sowerby
- Sarah Bland
- Julia Leach

Regent House Surgery

- Mark Sloan (PI)
- Christine Shepherd

Riverside Medical Practice

- Mark Ashworth (PI)
- Catarina Guerreiro
- Ross Dyer-Smith
- Jayshireen Singh

Rosedale Surgery

- Maarten Derks (PI)
- Karen Read
- Jodie Button

School Lane Surgery
- Martin Hadley-Brown (PI)
- Sandra Smith
- Caroline Hutson
- Barbara Stewart
- Karen Norcott

Scott Practice
- Andrew Slattery (PI)
- Davinder Singh
- Narinder Dhillon
- Rose Fells

Snaith & Rawcliffe Medical Group
- Susie Foster (PI)
- Liz Tomlinson
- Michaela Coutts

Station House Surgery
- Kathryn Morgan (PI)
- David Cowling
- Joanna Beldon
- Caite Guest
- Bruce Helme
- Paula Melrose

Strawberry Place Surgery
- Daniel Tacagni (PI)
- Nikki Davies
- Angela Sanders
- Paul Harris
- Angela Juhasz
- Anne Jenkins

Streatham Common Practice
- Kirsteen Rakin (PI)
- Tracey Hayward-Allingham
- Samantha Kirby

- Jayshireen Singh

Tottenham Health Centre
- Kumani Jeyarajah (PI)
- Beata Guss
- Dorota Daukszewicz
- Yesim Ozcan
- Jacqueline Mburu
- Anna Swinburn

Vanbrugh Group Practice
- Jaisun Vivekanandaraja (PI)
- Jayshireen Singh

Village Practice (The)
- Preeti Pandya (PI)
- Stella Oldham

Wallington Family Practice
- Lindsey Roberts (PI)
- Julie Fuller
- Jayshireen Singh

Watling Medical Centre
- Murtaza Khanbhai (PI)
- Paula McLaren
- Jacqueline Mburu
- Anna Swinburn

West Hampstead Medical Centre
- Jonathan Barnett (PI)
- Letitia Coco-Bassey
- Jacqueline Mburu
- Veridiana Toledo

William Harvey Heart Centre
- David Collier (PI)
- Anne Zak
- Rebecca James
- Yasmin Choudhury
- Mary Feely
- Manish Saxena

- Julian Shiel

Windermere & Bowness Surgery

- Julia Colclough (PI)
- Elizabeth Butterworth
- Alison Crumbie
- Jill Barlow

Woodlands Practice

- Nicola Jayne Pascall (PI)
- Jayshireen Singh
