# Supplementary material for: Statin treatment and muscle symptoms: series of randomised, placebo controlled n-of-1 trials
Source: BMJ. 2021 Feb 24;372:n135. doi: 10.1136/bmj.n135 (PMC7903384; doi:10.1136/bmj.n135)
Supplement: Supplementary file 3 — Web appendix 3: StatinWISE protocol [file here059829.ww3.pdf]

A series of randomised controlled N-of-1 trials in patients who have discontinued or are considering discontinuing statin use due to muscle-related symptoms to assess if atorvastatin treatment causes more muscle symptoms than placebo

CLINICAL TRIAL PROTOCOL

PROTOCOL NUMBER: ISRCTN30952488

VERSION NUMBER	DATE	REASON FOR CHANGE
1.0	25 May 2016	First version
2.0	08 August 2016	Substation Amendment 1: addition of exclusion criterion and changes to Adverse Event reporting
2.1	28 October 2016	Non-substantial Amendment 1:<br>Correction of typographical errors
3.0	27 June 2017	Substantial Amendment 3.<br>i) modifying recruitment pathway description<br>ii) modifying Appendix 6 (PIS) and Appendix 7 (ICF)<br>iii) including Appendix 6a (PIS, optional genetic study), Appendix 7a (ICF, optional genetic study) and Appendix 9 (patient recruitment pathway)<br>iv) general editing of document
3.1	03 November 2017	Substantial Amendment 5.<br>i) recruitment period extended<br>ii) general editing of document

SUMMARY

**FULL TITLE OF STUDY**	A series of randomised controlled N-of-1 trials in patients who have discontinued or are considering discontinuing statin use due to muscle-related symptoms to assess if atorvastatin treatment causes more muscle symptoms than placebo		
**SHORT TITLE**	STATIN: WEB-BASED INVESTIGATION OF SIDE EFFECTS		
**TRIAL ACRONYM**	STATINWISE		
**PROTOCOL /ISRCTN NUMBER**	ISRCTN30952488		
**EUDRACT NUMBER**	2016-000141-31	**CLINICAL TRIALS.GOV**	NCT02781064

BACKGROUND: Statins are the most commonly prescribed treatment in the UK. Recently updated NICE guidelines have lowered the threshold for statin use to include all patients with a 10% or greater 10-year risk of cardiovascular disease. Previous randomised trials have established the prevalence of serious adverse effects of statins such as rhabdomyolysis, however, many patients discontinue statins due to less severe symptoms, such as muscle pain or fatigue. Randomised trials have shown no differences between those taking statin and placebo in terms of the prevalence of these side effects (approximately 9%), but currently there is no pathway of care for clinicians to empirically and objectively evaluate whether symptoms reported by a statin-user are caused by the statin itself or the so-called 'nocebo' effect (symptoms reflecting patient expectation of side effects). Given the effectiveness of statins in preventing cardiovascular disease, accurate data on the cause of symptoms experienced during statin use are needed to reliably inform patient and clinician about continuation of use. The proposed StatinWISE trial will provide definitive answers to this important uncertainty about statin therapy.

AIM: To determine whether statins cause muscle symptoms.

PRIMARY OUTCOME: Patient reported muscle symptoms (pain, weakness, tenderness, stiffness or cramp).

SECONDARY OUTCOME: Relationship between individual trial result and patient decision whether to continue statins long term.

TRIAL DESIGN: A series of randomised, double-blinded N-of-1 trials.

DIAGNOSIS AND INCLUSION/EXCLUSION CRITERIA:

Inclusion criteria:

- Adults (aged 16 and over)
- Prescribed statin treatment in the last 3 years
- Stopped OR considering stopping statin treatment due to muscle symptoms
- Provided fully informed consent.

Exclusion criteria:

- Any previously documented serum alanine aminotransferase (ALT) levels at or above three times the upper limit of normal;

• Have persistent, generalised, unexplained muscle pain (whether associated or not with statin use) and have creatinine kinase (CK) levels greater than 5 times the upper limit of normal<br>• Any contraindications listed in the Summary of Product Characteristics for Atorvastatin 20 mg (Appendix 8)<br>• Should not be using atorvastatin 20mg daily in the opinion of the general practitioner.	
**TEST PRODUCT, REFERENCE THERAPY, DOSE AND MODE OF ADMINISTRATION**: Once daily oral atorvastatin 20mg or placebo for 12 months.	
**SETTING:** This trial is coordinated from the Clinical Trials Unit at London School of Hygiene & Tropical Medicine and conducted in patients registered in General Practice in England and Wales.	
**DURATION OF TREATMENT AND PARTICIPATION:** Eligible patients should be randomised as soon as possible after the screening. Treatment period is for 12 months with a final follow-up within 3 months of the end of treatment period.	
**CRITERIA FOR EVALUATION:** Patients who enter data on muscle symptoms at least once during a treatment period with the IMP and at least once during a treatment period with placebo.	
**CLINICAL PHASE**	4
**PLANNED TRIAL START**	01/09/2016
**PLANNED DATE OF LAST PATIENT ENROLMENT**	31/07/2018
**PLANNED DATE OF LAST PATIENT FINAL FOLLOW-UP**	31/10/2019

CONTENTS

1. INTRODUCTION....4
2. TRIAL DESIGN....6
2.1 OVERVIEW.... 6
2.2 OPTIONAL GENETIC STUDY.... 7
2.3 NUMBER OF PARTICIPANTS NEEDED.... 7
2.4 SAMPLE SIZE....8
2.5 SETTINGS.... 8
2.6 RECRUITMENT OF PARTICIPANTS.... 9
2.7 ELIGIBILITY.... 9
2.8 RANDOMISATION.... 10
2.9 TREATMENT.... 11
2.9.1 DOSE SELECTION.... 11
2.9.2 DRUG MANUFACTURE, BLINDING AND SUPPLY OF TRIAL MEDICATION.... 11
2.9.3 ADMINISTRATION OF TRIAL MEDICATION.... 12
2.10 DATA COLLECTION.... 12
2.10.1 BASELINE AND RANDOMISATION DATA.... 13
2.10.2 TREATMENT PHASE FOLLOW UP DATA.... 13
2.10.3 END OF TRIAL DATA.... 14
2.10.4 PATIENT REPORTING INTOLERABLE SYMPTOMS....14
2.10.5 EARLY WITHDRAWAL OF PATIENTS FROM THE TRIAL.... 15
2.11 ADVERSE EVENTS.... 16
2.12 UNBLINDING.... 17
2.13 OUTCOME MEASURES.... 17
2.14 MONITORING.... 18
2.15 AUDITS AND INSPECTIONS.... 19
2.16 END OF TRIAL FOR PARTICIPANTS.... 19
2.17 ANALYSIS.... 19
3. TRIAL ORGANISATION AND RESPONSIBILITIES.... 21
3.1 SPONSORSHIP AND TRIAL MANAGEMENT.... 21
3.2 INDEMNITY.... 21
3.3 PROTOCOL DEVELOPMENT.... 21
3.4 INDEPENDENT DATA MONITORING COMMITTEE (DMC).... 22
3.5 TRIAL STEERING COMMITTEE (TSC).... 23
3.6 PRINCIPAL INVESTIGATOR'S' RESPONSIBILITIES.... 24
3.7 TRIAL MANAGEMENT GROUP AND CLINICAL TRIALS UNIT RESPONSIBILITIES.... 24
3.8 CONTACTING THE CTU IN AN EMERGENCY.... 25
3.9 PUBLICATION AND DISSEMINATION OF RESULTS.... 25
3.10 FINANCIAL SUPPORT.... 25
4. ABBREVIATIONS USED.... 26
5. REFERENCES.... 27
6. APPENDICES.... 29
Appendix 1: Trial Assessment Timelines.... 30
Appendix 2: Baseline Data Form.... 32
Appendix 3: End of Trial Form.... 34
Appendix 4: Patient Questionnaire....35
Appendix 5: VAS Pain Scale Data Form.... 38
Appendix 6: Patient Information Sheet.... 40
Appendix 6a: Patient Information Sheet – Optional Genetic Study....44
Appendix 7: Informed Consent Form....46
Appendix 7a: Informed Consent Form – Optional Genetic Study....47
Appendix 8: Summary of Product Characteristics for Atorvastatin 20 mg....48
Appendix 9: Recruiting pathway from advertising....67

1. INTRODUCTION

Statins reduce cardiovascular disease (CVD) risk[1] and are recommended as part of the treatment strategy for primary and secondary prevention of CVD in the NHS.[2] Although statins are the most commonly prescribed treatment in the UK,[3] there is still uncertainty about adverse effects.[4, 5]

Severe statin adverse effects are extremely rare (rhabdomyolysis 0.1 and myopathy 0.5 per 1000 people over 5 years).[6] However, there has been widespread reporting of other less well-defined statin-related symptoms in the media, notably muscle pain and weakness. These reports have largely been prompted by data from non-randomised, non-blinded observational studies,[7, 8] but have not been confirmed in blinded randomised controlled trials (RCTs).[9] A major limitation of observational studies is lack of blinding: patients taking a medication expect to experience adverse effects[10] and therefore reporting of symptoms may be higher than in a comparable statin-free population. This phenomenon, the "nocebo" effect, can lead to bias in unblinded studies.

In RCTs expectations of adverse effects can lead to inflated reporting in both active and placebo groups. In the Odyssey ALTERNATIVE trial, statin 'intolerant' patients initially underwent a double-blind four week phase where they received placebo. Interestingly, during this time, 7% dropped out due to myalgia. In the main phase of this three armed trial (alirocumab vs ezetimibe vs atorvastatin), rates of adverse events were the same across all groups at roughly 80%, but dropped to 55% among alirocumab when unblinded.[11] Therefore in some trials of statins, expectation of adverse effects among both placebo and active treatment arms may have diluted any true effect of statins on muscle symptoms. A systematic review of randomised trials of statins found that the prevalence of myalgia varied from 0% to 30%, but was not different in the active and placebo arms.[9]

There have also been two other important criticisms of the existing RCT evidence. First, not all trials have collected data on subjective symptoms and recording may be inconsistent due to the definitions used. Studies have shown that adverse events are rarely fully presented in journal publications.[12] Second, while there have been trials among specific vulnerable patient groups,[13-15] there is a perception that trial participants do not reflect populations taking statins in routine care.

We are working closely with the Cholesterol Treatment Trialists' Collaboration performing a meta-analysis of statin adverse events reported in RCTs of statins, which will determine using individual participant data whether there is an excess of adverse events among those allocated statin compared with placebo, and whether to date there has been any bias in the reporting of adverse events in statin trials. However, for the reasons outlined above, re-analysis of the existing evidence may not alleviate individual patient concerns.

Despite evidence-based recommendations about the risks and benefits of statin use, many patients believe their symptoms are due to statins, leading to discontinuation, and are therefore potentially missing out needlessly on an effective drug intervention. General Practitioners (GPs) are faced with the challenge of decision making when patients present with symptoms during statin use as there

is currently no diagnostic tool allowing clinicians to empirically evaluate whether symptoms reported by an individual statin user are caused by the statin itself or by the 'nocebo' effect.

N-of-1 trials offer the opportunity for individual patients to discover whether the symptoms that they are experiencing are attributable to statins. Each patient acts as their own control, and therefore their optimal treatment can be established.[16, 17] Importantly, N-of-1 trials offer individual patients the opportunity to observe their own response to active and placebo treatments. We anticipate that this knowledge will be an important determinant of subsequent statin use and therefore we will follow patients for three months after their blinded treatment period has ended. At the end of these three months we will be able to ascertain as to whether they are using statins.

The proposed trial will address some of the criticisms of previous evidence. The trial will be blinded and placebo-controlled to minimise bias and the sequence of statin and placebo treatments will be randomised to avoid confounding. Additionally, the within-patient comparisons of symptoms experienced while on placebo and statins will allow us to determine, definitively: (i) whether individual patient symptoms are caused by statins and (ii) the extent to which symptoms attributed to statins appear to be causally related.

2. Trial design

2.1 OVERVIEW

StatinWISE is a randomised, double-blind, placebo controlled N-of-1 trial taking place in a primary care setting, to quantify the occurrence of self-reported muscle symptoms whilst taking daily atorvastatin. A total of 200 patients who fulfil the eligibility criteria will be recruited.

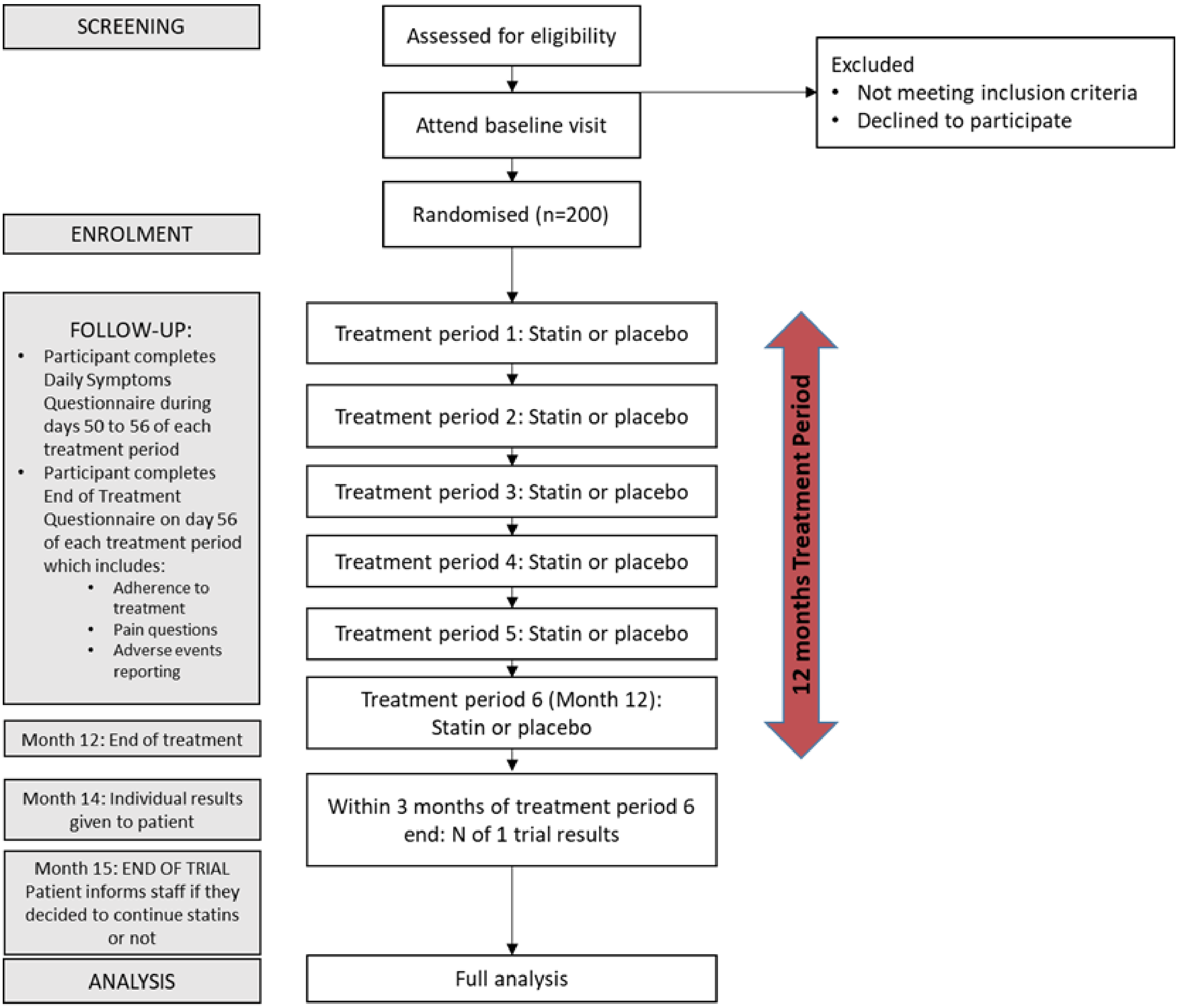

Figure 1: Trial overview

2.2 OPTIONAL GENETIC STUDY

A genetic component to statin myopathy (pain accompanied by rises in CK or ALT) has been suggested (the SLCO1B1 variant).[18] However, it is not clear whether this variant will be found among the patients included in our study (who have experienced muscle symptoms while on statins but no substantial rises in CK or ALT). Therefore, as part of the trial, we will ask patients to provide a blood sample for an optional genetic analysis as part of a larger collaborative effort investigating genetic associations with statins' effects. Specific results will not be fed back to clinicians or to patients.

Where patients have consented for the genetic blood test, one nine millilitres blood sample will be obtained in an EDTA Vacutainer tube, labelled with the genetic study ID number, patient's gender, and the date and time of sample collection. It will then be sent to the genetics laboratory at the University of Liverpool via first class post in pre-paid Royal Mail Safeboxes (each containing a maximum of four tubes) as soon as possible after collection. No patient identifiable data will be included or separately provided to the University of Liverpool.

2.3 NUMBER OF PARTICIPANTS NEEDED

A feasibility study in the Clinical Practice Research Datalink has indicated that on average 35 patients per practice per year will be eligible to take part in the trial. We anticipate an average uptake of 15% among primary care patients invited to participate in our trial. Therefore, we estimate that we will need to invite approximately 1300 patients from over 50 general practices to the trial to achieve our recruitment target of 200.

Power calculations: Our power calculation was performed via simulation.[19] An initial sample size, *n*, was chosen. The treatment allocation sequence for each of the *n* patients was drawn by randomly allocating the participant to either statin or placebo separately for periods 1, 3, and 5, thereby determining the order of the 6 treatment periods. For each participant, a random effect was drawn from a normal distribution, with zero mean and variance equal to 30^2. Residual errors (6 for each participant; one for each period) were drawn from a normal distribution with zero mean and variance equal to 25^2. Then a VAS pain score was simulated for each participant in each period, adding a treatment effect of 10 to each statin period, incorporating the patient random effect and the participant's residual error for that period. This simulated dataset was analysed using the primary analysis model (as if this were the real data) and the two-sided p-value for the treatment effect was stored. After repeating this process a large number of times (e.g. 500), the statistical power was estimated by the proportion of simulated datasets in which the P-value was less than 0.05. If the power was less than 90%, the sample size was increased and the power re-estimated. The rationale for the parameters chosen within this model is detailed below.

Minimum clinically significant difference in VAS pain score (10 mm): This value was chosen to represent the smallest VAS change in pain which patients would perceive as being beneficial, and might therefore change the patient's decision regarding subsequent statin use. Two studies conducted within an Accident and Emergency setting[20-23] both concluded that the smallest change in VAS pain score corresponding to "a little more" or "a little less" pain was 13mm, with confidence intervals (95% CI: 10 to 17mm) and (95% CI: 10 to 16mm). We took the lower limit of the confidence interval to represent the smallest change likely to be perceived as being beneficial.

Within and between-participant variability in VAS pain score: (30^2 and 35^2, respectively). These values were obtained by fitting a mixed model to the data from a pilot series of N-of-1 trials for statin adverse effects[24] (data obtained by approximation from figures presented therein). These

variance components can be poorly estimated, thus we took values from the higher end of the confidence intervals, giving conservative estimates of these components.

2.4 SAMPLE SIZE

A sample size of 64 participants provides approximately 90% power to detect a treatment effect of at least 10mm, assuming a Type I error of 5%. Allowing for loss to follow-up of 40% of participants through the trial inflates the required sample size to 107 participants.

Period effects (changes in underlying VAS pain score due to factors other than randomised treatment, e.g. seasonal, activity-related, etc.), variability in individual statin effects across patients, and imperfect adherence to the assigned treatment were investigated by further detailed simulations. VAS pain scores are not normally distributed, since they are bounded (0-100mm) and can display large fluctuations in response. Therefore, further power calculations were performed drawing the outcome from a Beta distribution, and from a distribution with normal variance components on a logit scale, to assess the robustness of the sample size estimates to the distribution chosen. These factors all have the effect of decreasing power, thus increasing the sample size required. An approximate 80% increase in the sample size required in the absence of these effects provided approximately 90% or more power across a plausible range of these potential effects, thus we determined that a final sample size of 200 was required.

Multiple testing: Rather than making formal adjustments for multiple testing, we follow an approach advocated by Pocock[25] of clearly specifying our primary analysis (which provides a single test for treatment effect), while explicitly presenting and interpreting all other tests as secondary analyses.

Power for individual N-of-1 trials: In order to increase the statistical power for the analysis of individual N-of-1 trials, we will ask participants to report symptoms daily in the last week of each period, rather than once per period. Full adherence through the trial will provide between 55 and 70% power to detect effects of 10mm or greater for individual treatment comparisons.

Estimates of recruitment and retention rates: We anticipate that some patients will not complete data in each treatment period, and that some will not complete their 12-month follow-up. By designing this study as a series of N-of-1 trials, which offer individual participant benefit in the form of an individualised estimate of effect, we hope to minimise this type of dropout. However, we have accounted for this in our sample size calculation by allowing for 40% loss to follow-up.

2.5 SETTINGS

Research sites will be recruited through the Clinical Research Network across England and Wales and we will continue to add practices to ensure the sample size is achieved. Suitable collaborating practices and investigators will be assessed on their ability to conduct a trial. In advance of the trial starting at a practice, the Principal Investigator (PI) must agree to follow Good Clinical Practice

Guidelines and all relevant regulations. All relevant regulatory and ethics approvals must be in place prior to practices recruiting their first patient.

2.6 RECRUITMENT OF PARTICIPANTS

Participants will be recruited directly from GP Practices or by advertising to the public.

Participating practices will recruit eligible patients from two groups as follows:
1) Patients who are considering discontinuation of their statin due to muscle symptoms:
These patients will be invited to take part in the trial when they visit the GP to report muscle symptoms believed to be associated with statins and where the patient/GP is considering stopping statins because of the muscle symptoms. The GP or Research Nurse will approach the patient and give the patient information sheet (Appendix 6 and Appendix 6a). If interested, patients will be able to consent (Appendix 7 and Appendix 7a) and complete the screening visit with the GP or the Research Nurse during this appointment or it can be arranged for another suitable time.

2) *Patients who have stopped taking a statin in the last 3 years due to muscle symptoms:*
A search of the practice electronic records will be performed by the delegated site staff on a two-monthly basis for one year (or until recruitment targets are reached) to identify potentially eligible patients. All screened patients will be documented on a screening log. The list will be reviewed by the GP to confirm clinical eligibility before patients are invited to take part. A letter inviting them to attend a screening visit, accompanied with the patient information sheet (Appendix 6 and Appendix 6a) for the patient to consider, will be sent by the trial team from their GP practice. Contact details of the Research Nurse will be provided should the patient have any questions. A reply slip will be enclosed for the patient to complete if they wish to attend the screening visit, which will be returned to the Clinical Trials Unit (CTU), during which the trial will be explained, and they will have the opportunity to ask questions. Patients will be sent a letter of invitation to consider participation up to a maximum of three times.

3) *Patients who contact the CTU from advertising:*
Patients who contact the CTU in response to advertising material will be sent a letter to request their GP details on a reply slip. Following receipt of these documents the CTU will contact their GP with their consent. The GP will be asked to confirm that the patient is potentially suitable for the trial and to provide brief clinical information to allow eligibility to be assessed. This information will then be provided to the research site responsible for recruiting the patient.

Once informed consent has been obtained, eligibility, demographic and medical history data (Appendix 1) will be collected directly onto an online trial database.

2.7 ELIGIBILITY

Inclusion criteria:

- Adults (aged 16 and over)
- Registered in a participating GP practice

- Previously prescribed statin treatment in the last 3 years
- Stopped OR is considering stopping statin treatment due to muscle symptoms
- Provided fully informed consent.

Exclusion criteria:

- Any previously documented serum alanine aminotransferase (ALT) levels at or above three times the upper limit of normal
- Have persistent, generalised, unexplained muscle pain (whether associated or not with statin use) and have creatinine kinase (CK) levels greater than 5 times the upper limit of normal
- Any contraindications listed in the Summary of Product Characteristics for Atorvastatin 20 mg (Appendix 8)
- Should not participate in the trial in the opinion of the general practitioner.

2.8 RANDOMISATION

Patients eligible for inclusion will be randomised by the Research Nurse/GP practice trial team using the online London School of Hygiene & Tropical Medicine (LSHTM) CTU randomisation system to a sequence of blinded placebo and atorvastatin treatment periods. There are six paired (statin-placebo or placebo-statin) treatment periods each of two months' duration. In practice, this means that each individual is randomised (with equal probability) to one of the following eight sequences (with P=placebo, S=statin):

	**Treatment Period**					
	**1**	**2**	**3**	**4**	**5**	**6**
**Sequence 1**	S	P	S	P	S	P
**Sequence 2**	S	P	S	P	P	S
**Sequence 3**	S	P	P	S	S	P
**Sequence 4**	S	P	P	S	P	S
**Sequence 5**	P	S	S	P	S	P
**Sequence 6**	P	S	S	P	P	S
**Sequence 7**	P	S	P	S	S	P
**Sequence 8**	P	S	P	S	P	S

Randomisation codes will be generated and secured by the Information Technology team at LSHTM CTU, who has procedures to ensure the trial team remains blinded. The codes will be made available to a Good Manufacturing Practice (GMP) certified clinical trial supply company explicitly for the treatment packs to be created in accordance with the randomisation list.

2.9 TREATMENT

The trial treatment consists of once-daily oral administration of Atorvastatin (20mg) capsules, which will be compared with matching placebo (Microcrystalline Cellulose). The treatment phase of the trial will be one year in duration for each patient. A blinded placebo, identical in size, colour, smell and packaging to the active statin, has been chosen to prevent knowledge of treatment from affecting symptom scores.

Two months' supply of allocated treatment will be posted to the patient every 2 months. Treatment should start within 4 weeks of randomisation. Patients who have not started by the 3rd week post randomisation will be contacted by the CTU. Such patients will therefore have a longer study period.

2.9.1 DOSE SELECTION

Atorvastatin is recommended by the current NICE guidelines for lipid modification;[2] 20mg is the recommended dose for primary prevention of cardiovascular disease. Atorvastatin is also recommended by NICE for secondary prevention[2] and for patients with a high risk of adverse events (our patient population), a dose lower than 80mg is recommended. The most commonly used dose is 20mg. A visually matched placebo has been chosen as an appropriate comparator for two reasons. Firstly, patient expectation of symptoms while on statins is likely to affect their experience of symptoms. A placebo control should minimise bias arising from knowledge of allocation. Secondly, withholding statin treatment from patients during placebo treatment periods is justified because the trial will recruit patients who have recently stopped using statins (and therefore are not currently receiving any benefit from statins) and those who wish to discontinue. If patients in the trial tolerate the active treatment periods with few symptoms, then this trial is likely to increase their usage of statins in the long term.

2.9.2 DRUG MANUFACTURE, BLINDING AND SUPPLY OF TRIAL MEDICATION

The active trial drug atorvastatin (as atorvastatin calcium trihydrate) will be purchased on the open market. Atorvastatin is manufactured by Sharp Clinical Services (UK) Ltd under Marketing Authorisation Number 10284. The Marketing Authorisation guarantees that the product has been manufactured and released in accordance with the United Kingdom's Good Manufacturing Regulations.

Placebo will be manufactured specially to match the atorvastatin by a GMP certified manufacturer. Capsules and packaging will be identical in appearance for both active treatment and placebo. DBcaps® capsules which has a unique locking mechanism to help with assuring the integrity of the blind will be used for over encapsulation of both active and placebo treatments. Additionally, patients will be asked to swallow the capsule whole without chewing or breaking it. The blinding process and first stage Qualified Person (QP) release will be done by the designated clinical trial supply company. The blinding process will involve encapsulating the active tablet, complete removal

of the original manufacturer's label and replacement with the clinical trial label bearing the randomisation number which will be used as the pack identification. Other pack label text will be identical for both atorvastatin and placebo treatments and will be in compliance with requirements for investigational medicinal products.

The designated clinical trial supply company will also be responsible for maintaining the Product Specification File (PSF) until final database lock and unblinding of the trial data. The CTU will be responsible for assuring all relevant approvals are available and that the patient is eligible for the trial before release of the trial medication to a patient. A separate Manual of Operating Procedures (MOP) will detail the drug accountability system. The MOP will also detail labelling of the trial treatment and other processes for assuring adherence to Good Manufacturing Practice.

2.9.3 ADMINISTRATION OF TRIAL MEDICATION

Patients will receive their allocated trial medications through the post and they will be asked to take the study medication daily through the six two-month treatment periods. Ideally there should be no break between treatment periods. However, if for any reason there is a break, patients can simply restart taking the trial medication as soon as possible.

Patients will be asked to take one capsule orally once daily at a time of day convenient to them. Capsules should be swallowed whole. Patients will be given written instructions on how to take the study medication. At the start of each two-month treatment period, patients will be asked to inform the trial team about their first date of study medication use using the trial's mobile application (app), email, text message, Freephone telephone service or a pre-paid postage reply slip - whichever is easier to ensure data collection occurs on the correct days. A Freephone telephone number will be provided for patients to call if they have any questions. Adherence to the study medication will be collected on the same days as other data as part of the outcome data collection.

2.10 DATA COLLECTION

This trial will be coordinated from the CTU at LSHTM and conducted at GP practices in England and Wales.

Baseline data will be collected and will be entered online to the trial database provided by the LSHTM CTU. Follow up data will be collected directly from each patient at the end of each two-month period.

Patients will be allowed to choose the method of data collection most suitable for them from the following:

1. Bespoke mobile app which will require patients to use their own smartphone.
2. Online database using a computer, phone or tablet.

3. Paper forms which they will receive by post at the same time with their trial treatment and which they can complete themselves or they can request a trial team member to contact them by phone to help with completing the forms.
4. Trial staff will telephone the patient on each data collection day and complete the questionnaire based on the patient answers.

For patients with a smartphone who choose to submit outcome data using the trial's bespoke mobile app, the GP, PI or Research Nurse will help the patient to install and set-up the app for use. The GP, PI or Research Nurse will also give a demonstration to ensure the patient understands how to use it. Each GP, PI or Research Nurse will have access to the internet so that the patient can download the app without using their own mobile network (ensuring that download of the app is free of charge to the patient).

The GP or Research Nurse will also show the patient how to complete the symptoms data for the Baseline form (Appendix 2) using their preferred method. Any questions can be addressed at this stage. Once baseline procedures have been completed and the patient confirmed as being eligible and consented, the patient can then be randomised.

A screening log will be used to record all patients who were identified as potentially eligible using the research site database, including those who were ineligible and those who declined participation. The Screening Log will remain at the relevant research site and only anonymised information regarding number of screened patients and number and reasons for screen failure will be shared with the CTU.

Only data outlined on the baseline, follow up, end of trial and adverse events data forms will be collected as part of the trial database.

2.10.1 BASELINE AND RANDOMISATION DATA

Baseline data will be collected by the PI, GP or Research Nurse performing the assessment at the Screening Visit (Appendix 2). Baseline data will be collected via a bespoke online trial database, to which each PI, GP and Research Nurse will have a unique log-in ID and password. Eligible patients will then be randomised and data about the randomisation (date, time and randomisation number) is generated.

2.10.2 TREATMENT PHASE FOLLOW UP DATA

In the seventh week of each treatment period, patients will receive reminders (format agreed at baseline) to alert them that follow-up data collection is approaching. During the 8th week of each treatment period, the Patient Questionnaire and VAS Pain Scale forms (Appendices 4 and 5) will be completed by the patient. Patients can choose to receive daily reminders on each day their data is due to be collected. Non-responders will automatically receive a reminder after 24 hours of due date by the trial team.

2.10.3 END OF TRIAL DATA

During the seventh week of treatment period 6, the GP, PI or Research Nurse will contact patients to thank them for their participation so far, inform them that this is the last treatment period, and that they, together with their GP, the PI and Research Nurse, will receive their individual results at the beginning of month 14. The Research Nurse and patient will arrange a telephone or face-to-face appointment to discuss the individual results during month 14. The Research Nurse will also inform patients that if they want to continue taking a statin without a break, to arrange a separate clinical appointment with their GP prior to the end of the treatment period.

At month 15, trial staff will contact the patient to document their decision on future statin use and whether their results helped reach this decision. This will be the last data collection point of the trial.

Throughout the trial, continued patient care will be at the discretion of their GP. In primary care, the patient will be recorded as having an ongoing statin prescription.

- Where treatment with an interacting drug is needed that will be for less than one-month duration, the patient will be asked to stop the trial treatment for that period.
- Where treatment with an interacting drug is needed for longer than one month, the patient will be asked to withdraw from study treatment completely.

When the patient is randomised, a temporary Read code indicating StatinWISE participation will be recorded in their primary care record, the code will be removed at the end of the trial.

Patients will also be given an alert card that identifies them as a StatinWISE patient. Patients will be asked to present this card to anyone providing medical care outside of their usual general practice. This card will have a link to the trial website and a trial contact number.

2.10.4 PATIENTS REPORTING INTOLERABLE SYMPTOMS

The GP will remain as the first point of contact for patients during the trial for their care. Intolerable muscle symptoms should be reported to the GP who will provide clinical care as directed by the appropriate NICE guidelines. Where a patient reports intolerable symptoms, Figure 2 shows the decision making pathway for the GP and the patient.

For patients who experienced intolerable muscle symptoms, and who do not want to be withdrawn early from the trial, the GP will be asked to confirm that the patient still meets the trial eligibility criteria. Patients who continue to meet the eligibility criteria can be offered the following options by the GP, depending on their clinical judgement:

i. continue with their current treatment
ii. reduce the frequency of tablet use to every other day rather than daily
iii. stop for that treatment period and resume at the start of the next period

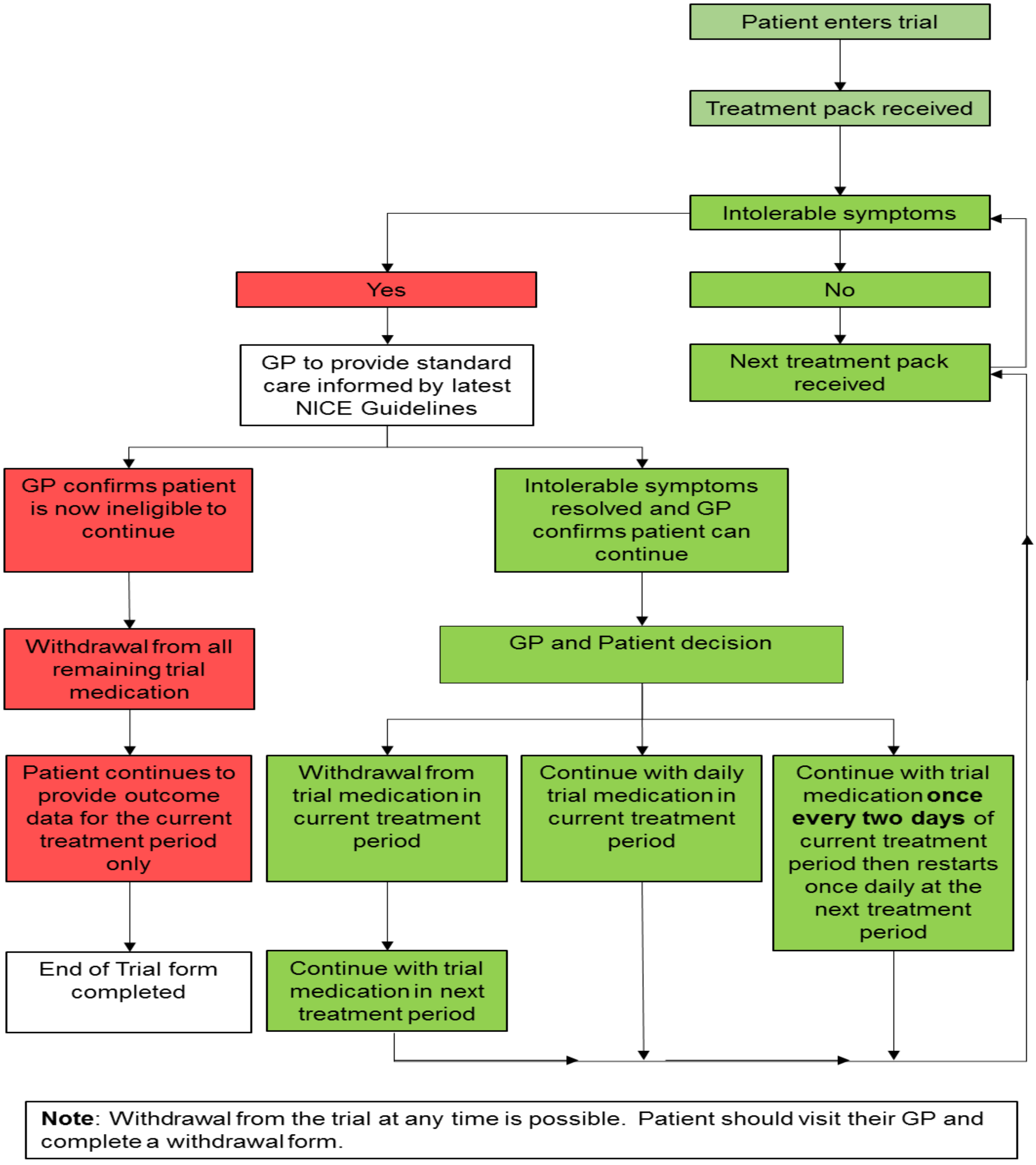

Figure 2: Intolerable symptoms pathway

Patients who withdraw from treatment temporarily or permanently will be asked to inform the study team and report symptoms at the time of stopping, and continue to submit outcome data for their current treatment period.

2.10.5 EARLY WITHDRAWAL OF PATIENTS FROM THE TRIAL

A patient is free to change their minds about participation at any time. We would advise that the patient see their GP to discuss future routine care. A GP can withdraw a patient at any time if clinical

concerns arise or if the patient presents with any reason to stop atorvastatin as described in the Summary of Product Characteristics for Atorvastatin 20 mg (Appendix 8). In each case, an end of trial form (Appendix 3) should be completed.

2.11 ADVERSE EVENTS

Reporting of Adverse Events for this trial: Atorvastatin is not a new drug and has a well-documented safety profile. Furthermore, this trial is being conducted in a population in which statin use is clinically indicated. It is anticipated that patients in this trial are at higher risk of cardiovascular diseases because of underlying clinical conditions requiring statin use. Additionally, as participation is for 15 months it is likely that they will have common medical problems (e.g. colds, coughs, fevers etc). On the basis that patients: (1) would have had prior exposure the trial treatment, (2) the trial treatment is clinically indicated for their medical condition, and (3) the known safety profile of the trial treatment, we will limit adverse events to be reported to those events that fulfill the Serious Adverse Event (SAE) criteria as described below.

Patient Self-report: In the two-monthly questionnaire, patients will be asked to self-report any event which resulted in unplanned hospital admissions. Where a patient indicates that there was an unplanned admission, the PI/GP/Research Nurse will be asked to submit a brief report to the CTU.

General Practitioner report: Participating research sites will report any event to the CTU that fulfill the Serious Adverse Event (SAE) criteria as described below within 24 hours of becoming aware of the event. To minimize the risk of unreported events, at two-monthly intervals, the Research Nurse/GP will conduct a search of their database for any StatinWISE patients who have been recorded as having died.

Definitions:

Adverse Event (AE): Any untoward medical occurrence not listed in the Investigational Medicinal Product Dossier (IMPD) and /or Summary of Product Characteristics (SmPC) affecting a trial participant during the course of a clinical trial.

Serious Adverse Event (SAE): A serious adverse event (experience) is an adverse event (as defined above) which at any dose:

- results in death
- is life-threatening
- requires inpatient hospitalisation or prolongation of existing hospitalisation; or
- results in persistent or significant disability/incapacity
- is a congenital anomaly/birth defect

Adverse Reaction (AR): An AE when there is at least a possibility that it is causally linked to a trial drug or intervention.

Serious Adverse Reaction (SAR): SAE that is thought to be causally linked to a trial drug or intervention.
Suspected Unexpected Serious Adverse Reaction (SUSAR): An *unexpected* occurrence of a SAR that is inconsistent with the list of expected events in the Summary of Product Characteristics for atorvastatin 20 mg dated 02/09/2015 (Appendix 8); there need only be an index of suspicion that the event is a previously unreported reaction to a trial drug or a previously reported but exaggerated or unexpectedly frequent adverse drug reaction.

All SAEs assigned by the PI or delegate as both suspected to be related to IMP-treatment and unexpected will be classified as SUSARs and will be subject to expedited reporting to the Medicines and Healthcare Products Regulatory Agency (MHRA). The Sponsor (or delegate) will inform the MHRA, and the ethics committee of UK-relevant SUSARs within the required expedited reporting timescales (no later than 7 days after the Sponsor was first made aware for fatal/life threatening reactions; no later than 15 days for other reactions).

As this is a blinded trial, all SUSARs must be reported to the Sponsor (or delegate) assuming the active compound is involved. All expedited reports submitted to the regulatory authorities will be unblinded.

2.12 UNBLINDING

In general, there should be no need to unblind the allocated treatment during the treatment phase. If some contraindication to statins develops after randomisation, the trial treatment can be stopped and all usual standard care given. Unblinding should be done only in those rare cases where the clinician believes that clinical management depends importantly upon knowledge of whether the patient is receiving statin or placebo. In those few cases when urgent unblinding is considered necessary, a 24-hour telephone service will be provided by the CTU and details provided in the Investigator's Study File and the Patient Alert Card. The caller will be told whether the patient is receiving statin or placebo. An unblinding report form will be completed by the person requesting the unblinding. Participation will not restart once unblinding has occurred.

In this trial, it is important that the CTU is contacted for unblinding as any request is likely to originate from emergency departments or hospital clinics needing to know the treatment allocation urgently. As most GPs and research surgery staff do not provide a 24-hour cover, for patient safety reasons, the CTU will provide a 24 hour on-call unblinding service.

2.13 OUTCOME MEASURES

Primary outcome: The primary outcome is self-reported 'muscle symptoms', defined as pain, weakness, tenderness, stiffness or cramp to the body of any intensity, recorded where the participant believes they are associated with the study medication; these are the symptoms most commonly reported by patients and are often the reasons for discontinuation. The primary outcome will be assessed by the mean difference in VAS scores (range 0 to 100) between treatment periods with the trial treatment and treatment periods with placebo, estimated via a linear mixed model.

Secondary outcomes: Secondary outcomes relating to participant belief about the cause of their muscle symptoms, the site of muscle symptoms, how the muscle symptoms affected the participant and information about any other symptoms will be collected on the last day of each two-month treatment period, using a questionnaire (Appendix 5). The other secondary outcomes are adherence to medication, the participant's decision about statin treatment following the trial, and whether they found their own trial result helpful.

Specifically, secondary outcomes will be:

1. The proportion of patients with muscle symptoms during each two-month period who report that they believe their symptoms were caused by the study medication, comparing periods of IMP treatment with placebo.
2. Site of muscle symptoms (single or multiple; head and neck/upper limbs/lower limbs/trunk).
3. Among patients reporting muscle symptoms, the difference in mean VAS scores (range 0 to 100) for the following, comparing periods of IMP treatment with placebo:

a. General activity
b. Mood
c. Walking ability
d. Normal work (includes both work outside the home and housework)
e. Relations with other people
f. Sleep
g. Enjoyment of life

4. Other symptoms that the patient believes can be attributed to the study medication (grouped: musculoskeletal; gastrointestinal; respiratory; neurological; psychological; other).
5. Adherence to study medication as assessed by: (a) self-report and (b) counting pills remaining in returned packages, and the relationship between adherence and muscle symptoms.
6. Participant decision regarding future statin use and the relationship to their primary outcome.
7. Whether patients found their own trial result helpful in making the decision about future statin use.

2.14 MONITORING

GCP section 5.18.3 states in regard to monitoring, *"The determination of the extent and nature of monitoring should be based on considerations such as the objective, purpose, design, complexity, blinding, size and endpoints of the trial. In general, there is a need for on-site monitoring, before, during, and after the trial; however, in exceptional circumstances the sponsor may determine that central monitoring in conjunction with procedures such as investigators training and meetings, and extensive written guidance can assure appropriate conduct of the trial in accordance with GCP. Statistically controlled sampling may be an acceptable method for selecting the data to be verified."*

StatinWISE is a pragmatic, randomised placebo controlled trial. The intervention (atorvastatin) has marketing authorisation in the United Kingdom and has been in clinical use for decades. The trial will collect data on SAEs which may be associated with this product and other clinical conditions

which will be common in this population. These events will be reviewed routinely by the trial Medical Advisor and will be reported routinely to the Trial Steering Committee. The trial involves getting consent, giving the trial drug in the usual way and collecting information regarding muscle symptoms directly from the patients. There are no extra tests or procedures unless patients agree to take part in the genetic study, for which 9 ml of blood is required. Apart from the trial drug, any other medication will be issued as per usual practice under the care of the GP. For these reasons, we believe that the risk of harm or injury (whether physical, psychological, social or economic) to trial patients is low. We will use central monitoring along with investigators' training and meetings, and extensive written guidance to make sure the trial is carried out properly. Statistically controlled sampling will be used to select data to be verified at research sites. We plan to carry out on-site monitoring where central statistical monitoring show abnormality. Consent forms will be monitored centrally at the CTU.

Investigators/practices are required to provide direct access to source data/documents for trial-related monitoring, audits, ethics committee review and regulatory inspection. Source data will be low as the majority of patients will enter data using the mobile application method directly. All trial-related and source documents must be kept for five years after the end of the trial.

2.15 AUDITS AND INSPECTIONS

The trial may be subject to audit by the London School of Hygiene & Tropical Medicine under their remit as Sponsor, the Study Coordination Centre and other regulatory bodies to ensure adherence to GCP.

2.16 END OF TRIAL FOR PARTICIPANTS

The follow-up period ends 15 months after the first treatment day with a final contact with the PI, GP or Research Nurse as described above. This will be considered as the end of trial for patients.

2.17 ANALYSIS

Individual N-of-1 trials: The purpose of these analyses is to inform the individual patient of the effect of the IMP on their VAS muscle symptom score. The analysis and presentation of individual level results will be developed in collaboration with the Patient and Public Involvement (PPI) group. We will examine a range of graphical summaries and statistical analyses in order to identify the most informative presentation of individual results. As a result of this process, at the end of each individual trial the participant will be shown numerical and graphical summaries of their individual data, in relation to their IMP and placebo periods.

Combined analysis of N-of-1 trials

Primary analysis: To estimate the population average estimate of the trial treatment *n* VAS muscle symptom score, data from each N-of-1 trial will be aggregated to form a powerful dataset. We will adopt an intention-to-treat approach. Patients who enter data on muscle symptoms at least once

during a treatment period with the IMP and at least once during a treatment period with placebo will be included in the primary analysis.

The primary analysis will be a linear mixed model for VAS muscle symptom score with random effects for participant and treatment and fixed period effects. Residual errors will be modelled using a first-order auto-regressive error structure within each treatment period to account for correlation between the 7 daily measurements, with robust standard errors to account for non-normality of the VAS scores. Although VAS muscle symptom scores are unlikely to be exactly normally distributed, analysing such data using normal-based methods is likely to be a sufficiently robust approach.[26] All tests will be two-sided. $p < 0.05$ will be considered statistically significant.

Secondary analyses: Secondary outcomes relating to the impact of the IMP on other aspects of life will be analysed in a similar manner to the primary outcome, omitting the auto-regressive correlation structure since these secondary outcomes are measured once per treatment period.We will investigate whether the excess muscle symptoms, if any, experienced during treatment periods with the IMP appears to be concentrated in multiple sites.

Descriptive statistics will be used to summarise the measures of adherence to randomised treatment, and their relationship to the IMP and placebo periods. We will use the measures of adherence to randomised treatment to perform an efficacy analysis based around an instrumental variables approach.[27] Because these analyses require much stronger assumptions than the intention-to-treat analysis above, the results of the efficacy analysis will be presented and interpreted as a secondary analysis. The secondary outcomes include a single binary measure of whether the participant reports having muscle symptoms during that treatment period or not. This will be combined with the follow-up question pertaining to attribution, to obtain a single binary measure of whether the participant reports having muscle symptoms that they attribute to the study medication or not. These two binary outcome measures will be assessed using a logistic mixed model with random participant and treatment effects and fixed period effects.

We will relate the patients' decision regarding future statin use, and whether or not the participant found their own result helpful in making their subsequent treatment decisions, to their individual estimated effect of the IMP.

Subgroup analyses: There are no priori subgroup analyses planned. If an overall population-level effect is detected, we may investigate whether the effect varies within subgroups defined by measured baseline characteristics. These analyses will be regarded and interpreted as being exploratory.

3. Trial Organisation and Responsibilities

3.1 SPONSORSHIP AND TRIAL MANAGEMENT

The StatinWISE trial is sponsored by the LSHTM and its responsibilities coordinated by the CTU. The CTU may delegate responsibilities to third parties which will be outlined in relevant agreements. The responsibilities of the CTU will be overseen by the Trial Management Group (TMG).

3.2 INDEMNITY

LSHTM accepts responsibility attached to its sponsorship of the trial and, as such, would be responsible for claims for any non-negligent harm suffered by anyone as a result of participating in this trial. The indemnity is renewed on an annual basis and LSHTM assures that it will continue renewal of the indemnity for the duration of this trial.

3.3 PROTOCOL DEVELOPMENT

The Protocol Committee consists of the following investigators who will be responsible for the development of and agreeing to the final protocol. Subsequent changes to the final Protocol will require the agreement of the Trial Steering Committee (TSC).

NAME	AFFILIATION
Jane Armitage	University of Oxford, UK
Danielle Beaumont	LSHTM, London, UK
Elizabeth Crellin	LSHTM, London, UK
Ben Goldacre	LSHTM, London, UK
Emily Herrett	LSHTM, London, UK
Ian Roberts	LSHTM, London, UK
Haleema Shakur	LSHTM, London, UK
Liam Smeeth	LSHTM, London, UK (Chair)
Tjeerd van Staa	University of Manchester, UK
Elizabeth Williamson	LSHTM, London, UK
Nabila Youssouf	LSHTM, London, UK
Kieran Brack	LSHTM, London, UK

Lori Miller	LSHTM, London, UK

3.4 INDEPENDENT DATA MONITORING COMMITTEE (DMC)

MEMBERSHIP

NAME	AFFILIATION	EXPERTISE
Professor John Norrie	Chair of Medical Statistics and Trial Methodology.<br>Director of Edinburgh Clinical Trials Unit (ECTU), University of Edinburgh	Chair – Senior Statistician & Trialist
Professor Nicholas L Mills	Chair of Cardiology and Consultant Cardiologist, BHF Senior Clinical Research Fellow	Clinical Expert
Dr Hannah Castro	Freelance	Medical Statistician

To provide protection for study participants, an independent DMC has been appointed for this trial to oversee the safety monitoring. The DMC will review on a regular basis accumulating data from the ongoing trial and advise the TSC regarding the continuing safety of current participants and those yet to be recruited, as well as reviewing the validity and scientific merit of the trial.

The DMC composition, name, title and address of the chairman and of each member, will be given in the DMC Charter which will be in line with that proposed by the DAMOCLES Study Group (DAMOCLES Study Group 2005). Membership includes expertise in the relevant field of study, statistics and research study design. An independent statistician will be appointed to provide the analysis service required by the DMC.

The DMC Charter includes, but is not limited to, defining:

- the schedule and format of the DMC meetings
- the format for presentation of data
- the method and timing of providing interim reports
- stopping rules

STANDARD OPERATING PROCEDURES

The DMC has the responsibility for deciding whether, while randomisation is in progress, the unblinded results (or the unblinded results for a particular subgroup), should be revealed to the TSC. The DMC Charter states that they will do this if, and only if, the following two conditions are satisfied: (1) the results provide proof beyond reasonable doubt that treatment is on balance either definitely harmful or definitely favorable for all, or for a particular category of participants in terms of the major outcome; and (2) the results, if revealed, would be expected to substantially change the prescribing patterns of clinicians who are already familiar with any other trial results that exist. Exact criteria for "proof beyond reasonable doubt" are not, and cannot be, specified by a purely mathematical stopping rule, but they are strongly influenced by such rules. The DMC Charter is in agreement with the Peto-Haybittle stopping rule whereby an interim analysis of a major endpoint would generally need to involve a difference between treatment and control of at least three

standard errors to justify premature disclosure (Haybittle 1971; Peto 1977). An interim subgroup analysis would, of course, have to be even more extreme to justify disclosure. This rule has the advantage that the exact number and timing of interim analyses need not be pre-specified. In summary, the stopping rules require extreme differences to justify premature disclosure and involve an appropriate combination of mathematical stopping rules and scientific judgment.

3.5 TRIAL STEERING COMMITTEE (TSC)

The composition of the TSC is provided in the table below:

NAME	AFFILIATION	EXPERTISE
Michael Moore	University of Southampton	Independent Chair<br>Professor of Primary Healthcare Research and General Practitioner
Brian Mac Kenna	Chief Pharmaceutical Office<br>Medical Directorate, NHS England	Independent member 2<br>Pharmacist
Irwin Nazareth	University College London and Keat's GP Practice, Hampstead	Independent member 3<br>Professor of Primary Care and General Practitioner
Maurice Hoffman	StatinWISE PPI Group	Independent member 4<br>Patient Representative
David Symes	StatinWISE PPI Group	Independent member 5<br>Patient Representative
Rebecca Harmston	StatinWISE PPI Group	Independent member 6<br>Patient Representative
Haleema Shakur	LSHTM	Trials Expert<br>Senior Lecturer
Liam Smeeth	LSHTM	Chief Investigator<br>Professor of Clinical Epidemiology and General Practitioner

The role of the TSC is to provide overall supervision of the trial. In particular, the TSC will concentrate on the progress of the trial, adherence to the protocol, patient safety and consideration of new information. The TSC must be in agreement with the final protocol and, throughout the trial, will take responsibility for:

- ensuring relevant regulatory approvals are in place
- major decisions such as a need to change the protocol for any reason
- monitoring and supervising the progress of the trial
- reviewing relevant information from other sources
- informing and advising the TMG on all aspects of the trial

Face-to-face meetings or teleconferences will be held at regular intervals determined by need, but no less than once a year. A TSC Charter, which will detail how it will conduct its business, will be agreed at the first meeting.

3.6 PRINCIPAL INVESTIGATOR'S' RESPONSIBILITIES

Coordination within each participating research site will be through a local PI whose responsibility will be detailed in an agreement in advance of starting the trial and will include:

- ensure all necessary regulatory approvals are in place prior to starting the trial
- delegate trial related responsibilities only to suitably trained and qualified personnel
- train relevant medical and nursing staff who see patients and ensure that they remain aware of the state of the current knowledge, the trial and its procedures (there are wall charts, pocket summaries and PowerPoint presentations to assist with this)
- agree to comply with the final trial Protocol and any relevant amendments
- ensure that all patients who meet the eligibility criteria are considered promptly for the trial
- ensure consent is obtained in line with local approved procedures
- ensure that the patient baseline data are completed and transmitted to the CTU in a timely manner
- ensure the Investigator's Study File is up-to-date and complete
- ensure all adverse events are reported promptly to the CTU
- ensure the trial is conducted in accordance with ICH GCP and fulfils all national and local regulatory requirements
- allow access to source data for monitoring, audit and inspection
- be responsible for archiving all original trial documents including data forms for five years after the end of the trial

3.7 TRIAL MANAGEMENT GROUP AND CLINICAL TRIALS UNIT RESPONSIBILITIES

The TMG will consist of the Protocol Committee members (Section 3.3) plus trial manager(s), data manager and other key trial staff as required.

The CTU will act on behalf of the Sponsor and will be responsible to the TMG to ensure that all of the Sponsor's responsibilities are carried out. The responsibilities include (but are not limited to):

- report to the TSC
- maintain the Trial Master File
- identify trial sites
- confirm all approvals are in place before release of the trial treatment and the start of the trial at a site
- provide training about the trial
- provide study materials
- data management centre
- Investigational Medicinal Product management
- 24-hour advice and unblinding service
- give collaborators and patients regular information about the progress of the study
- respond to any questions (e.g. from collaborators and patients) about the trial
- ensure data security and quality and observe data protection laws

- safety reporting
- ensure trial is conducted in accordance with the ICH GCP including trial monitoring
- statistical analysis
- publication of trial results

3.8 CONTACTING THE CTU IN AN EMERGENCY

For urgent enquiries, adverse event reporting and unblinding queries investigators and patients can contact the 24-hour telephone service provided by the CTU. This number is given in the Investigator's Study File and the Patient Alert Card.

3.9 PUBLICATION AND DISSEMINATION OF RESULTS

The trial protocol and results will be published in peer-reviewed journals. All publications will follow the CONSORT statement.[21] Links to the publication will be provided in all applicable trial registers. Dissemination of results to patients will take place via the media, trial website (statinwise.Lshtm.ac.uk) and relevant patient organisations. Collaborating investigators will play a vital role in disseminating the results to colleagues and patients.

The success of the trial depends entirely upon the collaboration of nurses and doctors in the participating practices and those who hold key responsibility for the trial. Hence, credit for the study will be assigned to the key collaborator(s) from a participating site as it is crucial that those taking credit for the work have actually carried it out. The results of the trial will be reported first to trial collaborators.

3.10 FINANCIAL SUPPORT

The StatinWISE trial is funded by the NIHR Health Technology Assessment programme. Funding for this trial covers trial materials, meetings and central organisational costs. The design and management of the study are entirely independent of the manufacturers of atorvastatin, which is not a new product.

4. ABBREVIATIONS USED

AE	Adverse Event
ALT	Alanine aminotransferase
AR	Adverse Reaction
CK	Creatinine Kinase
CONSORT	Consolidated Standards Of Reporting Trials
CRF	Case Report Form
CTU	Clinical Trials Unit
CVD	Cardiovascular Disease
DMC	Data Monitoring Committee
GP	General Practitioner
GCP	Good Clinical Practice
GMP	Good Manufacturing Practice
ICH GCP	International Conference on Harmonisation of Good Clinical Practice
IMP	Investigational Medicinal Product
IMPD	Investigational Medicinal Product Dossier
LSHTM	London School of Hygiene & Tropical Medicine
mg	milligram
MOP	Manual of Operating Procedures
NICE	National Institute for Health and Care Excellence
PI	Principal Investigator
PSF	Product Specification File
QP	Qualified Person
RCT	Randomised Controlled Trial
SAE	Serious Adverse Event
SAR	Serious Adverse Reaction
SmPC	Summary of Product Characteristics
SUSAR	Suspected Unexpected Serious Adverse Reaction
TMG	Trial Management Group
TSC	Trial Steering Committee
VAS	Visual Analogue Scale

5. REFERENCES

1. Cholesterol Treatment Trialists C, Baigent C, Blackwell L, et al. Efficacy and safety of more intensive lowering of LDL cholesterol: a meta-analysis of data from 170,000 participants in 26 randomised trials. *Lancet* 2010; **376**(9753): 1670-81.
2. National Institute for Health and Care Excellence (NICE). Lipid modification: cardiovascular risk assessment and the modification of blood lipids for the primary and secondary prevention of cardiovascular disease. NICE clinical guideline 181. London, 2014.
3. Scholes S, Faulding S, Mindell J. Chapter 5: Use of Prescribed Medicines, Health Survey for England 2013. 2014.
4. Finegold JA, Manisty CH, Goldacre B, Barron AJ, Francis DP. What proportion of symptomatic side effects in patients taking statins are genuinely caused by the drug? Systematic review of randomized placebo-controlled trials to aid individual patient choice. *European journal of preventive cardiology* 2014; **21**(4): 464-74.
5. Maningat P, Breslow JL. Needed: pragmatic clinical trials for statin-intolerant patients. *The New England journal of medicine* 2011; **365**(24): 2250-1.
6. Armitage J. The safety of statins in clinical practice. *Lancet* 2007; **370**(9601): 1781-90.
7. Garavalia L, Garavalia B, Spertus JA, Decker C. Exploring patients' reasons for discontinuance of heart medications. *The Journal of cardiovascular nursing* 2009; **24**(5): 371-9.
8. Zhang H, Plutzky J, Skentzos S, et al. Discontinuation of statins in routine care settings: a cohort study. *Annals of internal medicine* 2013; **158**(7): 526-34.
9. Kashani A, Phillips CO, Foody JM, et al. Risks associated with statin therapy: a systematic overview of randomized clinical trials. *Circulation* 2006; **114**(25): 2788-97.
10. Barsky AJ, Saintfort R, Rogers MP, Borus JF. Nonspecific medication side effects and the nocebo phenomenon. *Jama* 2002; **287**(5): 622-7.
11. Moriarty PM. ODYSSEY ALTERNATIVE demonstrates complexity of statin-intolerant patients. AHA 2014; 2014.
12. Wieseler B, Wolfram N, McGauran N, et al. Completeness of reporting of patient-relevant clinical trial outcomes: comparison of unpublished clinical study reports with publicly available data. *PLoS medicine* 2013; **10**(10): e1001526.
13. Kjekshus J, Apetrei E, Barrios V, et al. Rosuvastatin in older patients with systolic heart failure. *The New England journal of medicine* 2007; **357**(22): 2248-61.
14. Heart Protection Study Collaborative G. MRC/BHF Heart Protection Study of cholesterol lowering with simvastatin in 20,536 high-risk individuals: a randomised placebo-controlled trial. *Lancet* 2002; **360**(9326): 7-22.
15. Baigent C, Landray MJ, Reith C, et al. The effects of lowering LDL cholesterol with simvastatin plus ezetimibe in patients with chronic kidney disease (Study of Heart and Renal Protection): a randomised placebo-controlled trial. *Lancet* 2011; **377**(9784): 2181-92.
16. Guyatt GH, Keller JL, Jaeschke R, Rosenbloom D, Adachi JD, Newhouse MT. The n-of-1 randomized controlled trial: clinical usefulness. Our three-year experience. *Annals of internal medicine* 1990; **112**(4): 293-9.
17. Lillie EO, Patay B, Diamant J, Issell B, Topol EJ, Schork NJ. The n-of-1 clinical trial: the ultimate strategy for individualizing medicine? *Personalized medicine* 2011; **8**(2): 161-73.
18. Search Collaborative Group, Link E, Parish S, et al. SLCO1B1 variants and statin-induced myopathy--a genomewide study. *The New England journal of medicine* 2008; **359**(8): 789-99.
19. Sakaeda T, Kadoyama K, Okuno Y. Statin-associated muscular and renal adverse events: data mining of the public version of the FDA adverse event reporting system. *PLoS One* 2011; **6**(12): e28124.
20. Todd KH, Funk JP. The minimum clinically important difference in physician-assigned visual analog pain scores. *Academic emergency medicine : official journal of the Society for Academic Emergency Medicine* 1996; **3**(2): 142-6.

21. Gallagher EJ, Liebman M, Bijur PE. Prospective validation of clinically important changes in pain severity measured on a visual analog scale. *Annals of emergency medicine* 2001; **38**(6): 633-8.
22. Cohen JD, Brinton EA, Ito MK, Jacobson TA. Understanding Statin Use in America and Gaps in Patient Education (USAGE): an internet-based survey of 10,138 current and former statin users. *Journal of clinical lipidology* 2012; **6**(3): 208-15.
23. Keech A, Collins R, MacMahon S, et al. Three-year follow-up of the Oxford Cholesterol Study: assessment of the efficacy and safety of simvastatin in preparation for a large mortality study. *European heart journal* 1994; **15**(2): 255-69.
24. Breivik H, Borchgrevink PC, Allen SM, et al. Assessment of pain. *British journal of anaesthesia* 2008; **101**(1): 17-24.
25. Arnold BF, Hogan DR, Colford JM, Jr., Hubbard AE. Simulation methods to estimate design power: an overview for applied research. *BMC medical research methodology* 2011; **11**: 94.
26. Dexter F, Chestnut DH. Analysis of statistical tests to compare visual analog scale measurements among groups. Anesthesiology, 1995, 82:896-902
27. White IR, Kalaitzakib E, Thompson SG. Allowing for missing outcome data and incomplete uptake of randomised interventions, with application to an Internet-based alcohol trial. Statistics in Medicine, 2011, 30: 3192–3207

6. Appendices

Appendix 1: Trial assessment timelines
Appendix 2: Baseline data form
Appendix 3: End of Trial data form
Appendix 4: Patient questionnaire
Appendix 5: VAS Pain Scale data form
Appendix 6: Patient Information Sheet
Appendix 6a: Patient Information Sheet, Optional Genetic Study
Appendix 7: Informed Consent Form
Appendix 7a: Informed Consent Form, Optional Genetic Study
Appendix 8: Summary of Product Characteristics for Atorvastatin 20 mg tablets
Appendix 9: Recruiting pathway

Appendix 1 StatinWISE Trial assessment timelines

	Database search	PIS posted to eligible patients	Baseline Visit	TP1 (month 1-2)	TP2 (month 3-4)	TP3 (month 5-6)	TP4 (month 7-8)	TP5 (month 9-10)	TP6 (month 11-12)	Follow-up Visit (F2F/Phone)	Secondary outcome data capture
**SCREENING**											
Trial team performs GP practice patients' list screening to identify potentially eligible patients	x										
GP reviews screening list and confirms patients are clinically eligible		x									
Trial team posts PIS with reply slip		x									
Patients contact trial team to arrange Baseline visit		x									
**ENROLMENT**											
Patients attend enrolment visit with Research Nurse and sign consent form			x								
Research Nurse completes the Baseline Form on electronic trial database. Baseline data will include:<br>- Personal Details<br>- Demographic data<br>- Eligibility assessment<br>- General medical history which may include blood test to measure total non-fasting cholesterol if none available in notes<br>- Randomisation data			x								

	Database search	PIS posted to eligible patients	Baseline Visit	TP1 (month 1-2)	TP2 (month 3-4)	TP3 (month 5-6)	TP4 (month 7-8)	TP5 (month 9-10)	TP6 (month 11-12)	Follow-up Visit (F2F/Phone)	Secondary outcome data capture
**ENROLMENT**											
Patients are randomised and trained on data entry tool of their choice			x								
**TREATMENT PERIOD**											
IMP posted to patients' address				x	x	x	x	x	x		
Patients confirm receipt of IMP				x	x	x	x	x	x	x	
Patients take study medication orally once daily				x	x	x	x	x	x	x	
Reminder to submit outcome data				x	x	x	x	x	x	x	
Patients enter outcome data and report any adverse events				x	x	x	x	x	x	x	
Patients post unused IMP to Pharmacy				x	x	x	x	x	x	x	
IMP accountability by Pharmacy				x	x	x	x	x	x	x	
Trial newsletter sent to patients						x			x		x
**FOLLOW-UP**											
Individual results ready to disclose										x	
Appointment (face-to-face or telephone call) with Research Nurse to discuss individual results										x	
**END OF TRIAL**											
Research Nurse telephones patients to record outcome data											x

Appendix 2: StatinWISE Baseline data form

1. Patient Initials				2. Patient Screening ID				-				3. Site ID		
4. Visit date	dd/mm/yyyy				5. Gender									
6. Ethnicity	a. White: (i) British (ii) Irish (iii) Traveller (iv) Other<br>b. Black: (i) African (ii) Caribbean (iii) Black British (iv) Other<br>c. Asian: (i) Asian British (ii) Indian (iii) Pakistani (iv) Chinese (v) Other<br>d. Mixed<br>e. Other: *(describe)*__________________											*Circle main group and sub-group*		
7. Age	[ ][ ] Years													

Patient Personal Details: The following information is needed for trial management purposes (sending trial medication and reminders) only and will not be collected as part of the trial database.

A. Full name	*First name*	*Last name*	
B. Postal address	House number/Name		
	Street name		
	Town/City		
	Postcode		
C. Email (primary)		D. Email (secondary)	
E. Home Telephone		F. Mobile number	

**ELIGIBILITY ASSESSMENT** *(Circling any RED box means patient is NOT eligible)*	**Eligibility** *Please circle*	
8. Written informed consent obtained?	YES	NO*
9. Is the patient aged 16 and over?	YES	NO*
10. Patient has a clinical reason for treatment with atorvastatin 20mg daily?	YES	NO*
11. Used statin within the last 3 years?	YES	NO*
12. Is patient considering discontinuation of their statin due to muscle related adverse effects<br>OR<br>Has patient stopped taking a statin due to muscle related adverse effects?	YES	NO*
13. Can atorvastatin 20mg daily be started or continued in the GP's opinion?	YES	NO*
14. Has patient had previously documented ALT levels at or above three times the upper limit of normal (ULN)?<br>Test date: dd/mm/yyyy Result: [ ][ ][ ] iU/L<br>(must be within 3 months of visit)	YES*	NO
15. Does the patient have persistent, generalised, unexplained muscle pain (whether associated or not with statin use) and have creatinine kinase (CK) levels greater than 5 times the upper limit of normal?<br>Test date: dd/mm/yyyy<br>(must be within 3 months of visit) Result: [ ][ ][ ] U/L	YES*	NO

***if circled, patient is NOT eligible**

PATIENT GENERAL MEDICAL HISTORY

16. Symptoms experienced during statin use that the patient believed were caused by the statin:

(a) Muscle pain	YES/NO	(f) Sore throat	YES/NO
(b) Joint pain	YES/NO	(g) Digestive problems	YES/NO
(c) Muscle weakness	YES/NO	(h) Headache	YES/NO
(d) Tiredness/fatigue	YES/NO	(i) Feeling sick	YES/NO
(e) Nosebleeds	YES/NO		

17. Does the patient think something other than statin may have caused their muscle pain? YES/NO

17(a) If YES to Question 17, please describe:

18. Does the patient have any history of cardiovascular disease? YES/NO

18a. If YES to Question 18, please indicate all that apply :

(a) Angina or diagnosed coronary heart disease	YES/NO	(c) Previous ischaemic stroke	YES/NO
(b) Previous myocardial infarction	YES/NO	(d) Peripheral vascular disease	YES/NO

(e) Other YES/NO (If yes, describe)_________________________________

19. Current use of high blood pressure medication? YES/NO

20. Blood pressure measurement (mmHg):

a. Systolic b. Diastolic

21. Diabetes? YES /NO

22. Smoking status: (a) non-smoker (b) ex-smoker (c) current smoker

23. Total cholesterol measurement [mmol/L] (within last 3 years):

If no reading, in line with NICE guidelines, organise non-fasting blood test for total cholesterol

24.
a. Height: cm
b. Weight: Kg

25. QRISK2 score:

26. Record all current medication:

Name of drug (generic)	Total daily dose and unit
1.	

27. Consent to participate in genetic study? YES/NO

27a. Blood sample taken? YES/NO

27b. Blood shipment reference:

RANDOMISATION DATA

28. Is patient fully eligible? YES/NO

29a. Date of Randomisation?

DD MM YYYY

29b. Time of Randomisation? *(24 hour clock)*

HOUR MINS

30. Randomisation Number:

31. Method of data collection chosen?

(a) Online system (b) Mobile application
(c) Phone call (d) Paper CRFs

31a. App successfully installed?

YES/NO

32. Training provided on method chosen?
YES/NO

33. Form completed by:

34. Date:

This data should be collected directly onto the online trial database (detail in your study file). If you are unable to enter this data, call 0207 299 4684

End of Trial Form (Month 15) / Early Withdrawal

1. Did the patient complete -the Trial to Month 15?

YES

A.1. Has the Research Nurse/GP spoken to the patient to discuss their individual results?
(i) YES
(ia)Date seen: :_____________ dd/mm/yyyy
(ii) NO – *(please arrange an appointment)*.
(iia) Date of appointment:_______ dd/mm/yyyy

A.2. Has the patient seen his/her GP since end of Treatment Period 6?
(i) YES
(ii) NO (*please remind the patient to book an appointment*)
(iia) Date reminder done: _______ dd/mm/yyyy

A.3. Has the patient been given a statin prescription in the 12 weeks since Treatment Period 6 ended?
(i) YES
(ia) Name of Statin:____________
(ib) Dose of statin:_____________
(ic) Date of issue of prescription:_____________
(ii) NO
(iia) If no statin has been prescribed, does the patient intend on re-starting long term statin therapy?
YES/NO/Don't know

A.4. Has the patient found their own trial results helpful to reach the decision as to whether to continue statin use?
YES/NO

NO

B.1. Please tick reason why the patient did not complete the trial:

☐ Lost to follow-up
☐ Early withdrawal
☐ Death

B.2. Lost to follow-up:
Date of last contact with patient? _________________
Dd/mm/yyyy

B.3. If Early withdrawal, give reason:
a. withdrawal of informed consent
b. intolerable muscle symptoms*
c. clinical concern*
d. other*

*: please enter details here: _____________________
____

B.4. Has the patient contacted their GP to discuss reason for withdrawal?
(i) YES
(ia)Date seen: :_____________ dd/mm/yyyy
(ii) NO – *(advise patient to contact GP)*.

B.5. Has the Research Nurse/GP spoken to the patient to discuss their individual results?
(i) YES
(ia)Date seen: :_____________ dd/mm/yyyy
(ii) NO – *(please arrange an appointment)*.
(iia) Date of appointment:_______ dd/mm/yyyy

B.6. Death
____

B.6.a. Date of death: _____________ dd/mm/yyyy

B.6.b Cause of death *(either autopsy report or clinical judgement if autopsy not available)*:
____

B.6.c. Date SAE form completed: ________dd/mm/yyyy
(if applicable)

C.1.This form was completed by:___________________________ Date: dd/mm/yyyy

StatinWISE Patient questionnaire, Version 2.1, 28/10/2016

Appendix 4: StatinWISE Patient questionnaire

INSTRUCTIONS

This questionnaire is to be filled out once every two months (on day 56). We will remind you one day before it is due. Please answer all questions. If you need any help to complete the form. Please call our Freephone number 08000 147410 or email the study team: statinwise@lshtm.ac.uk.

PART 1: ADHERENCE TO MEDICATION

Step 1 (all participants):

Q1. How would you best describe your use of study medication in the past two months? (Please choose one of the following options)

- ☐ I didn't take my study medication at all
- ☐ I took my study medication on some days
- ☐ I took my study medication on most days, but missed an occasional dose
- ☐ I took my study medications every day

Step 2 (appears if the participant chooses "I didn't take my study medication at all", or "I took my study medication on some days"):

Q2. What was the reason that you didn't take your study medication every day in the past two months? (please tick all that apply)

- ☐ I forgot to take my study medication

  Ticking the above will prompt the following option, if ticked, the trial team will action:
  ☐ Would you like a daily reminder sent to you via email/push notification

- ☐ I was prescribed another medication and my doctor told me to stop taking my study medication

  Ticking the above will prompt an alert for the trial team to contact surgery for details and consider withdrawal from study

- ☐ My doctor recommended that I stop taking study medication because of muscle symptoms

  Ticking the above will prompt an alert for the trial team to contact surgery for details

- ☐ I decided to stop taking study medication because of muscle symptoms

  Ticking the above will prompt an alert for the patient to contact the GP for advice

- ☐ I didn't want to take my study medication

  Ticking the above will prompt the following option, if ticked NO, the trial team will arrange for end of trial:
  Do you still wish to participate in the trial: ☐ Yes ☐ No

- ☐ Other (please specify)

(free text)

PART 2: EXPLORING SYMPTOMS IN THE PAST TWO MONTHS

1. *(all participants):* **In the last two months, have you experienced muscle symptoms, e.g. muscle pain or weakness?**
   - ☐ Yes
   - ☐ No

---

2. *(appears if the participant ticks yes for Question 1):* **Do you think these muscle symptoms are related to your study medication?**
   - ☐ Yes
   - ☐ No
   - ☐ Don't know

---

3. *(appears if the participant ticks no for Question 2):* **What do you think may have caused these muscle symptoms?**

   (free text)

   - ☐ Don't know

---

4. *(appears if the participant ticks yes for Question 1):* **Indicate with a cross (×) on the scale the number that describes how, during the last two months, pain has interfered with your:**
   a. General activity
   b. Mood
   c. Walking ability
   d. Normal work (includes both work outside the home and housework)
   e. Relations with other people
   f. Sleep
   g. Enjoyment of life

   For each of (a) to (g), the following scale will appear:

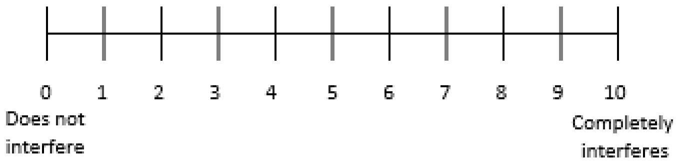

---

5. *(appears if the participant ticks yes for Question 1):* **On the diagram, tap to highlight the area that hurts the most.** *(Paper form will read: 'On the diagram, put an X on the area that hurts the most.')*

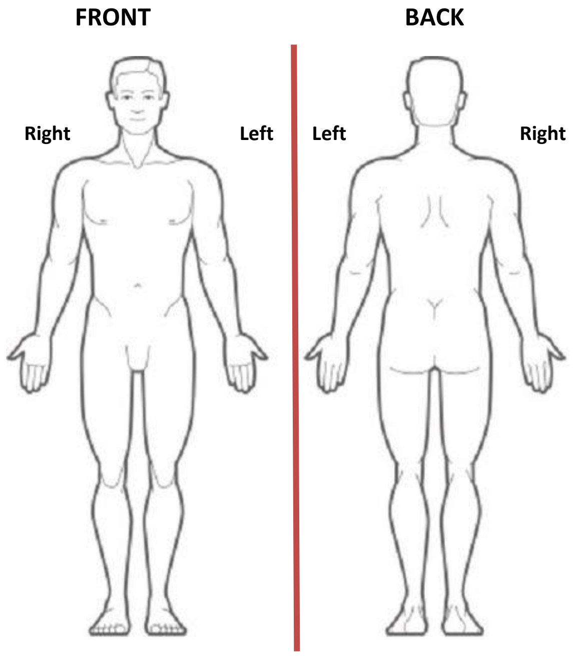

☐ More than one area

The body will be divided into the following locations and answers should be categorized depending where the participant taps. For paper forms, the location given by X marked on the body will be entered into the web or app version at data entry. Locations: head and neck/upper limbs/lower limbs/trunk.

---

6. *(All participants):* **In the last two months, have you experienced any other symptom that you think is associated with your study medication?**
   - ☐ Yes (please specify)
   (free text)
   - ☐ No
   - ☐ Don't know

PART 3: ADVERSE EVENTS

1. *(All participants):* **Have you been admitted to hospital in the last two months?**
   - ☐ Yes
   - ☐ No

2. *(Appears if the participant ticks yes for Question 1):* **What was the reason for your admission to hospital?**

(free text)

StatinWISE VAS Pain Scale data form, Version 1.0, 23/05/2016

Appendix 5: StatinWISE VAS Pain Scale data form

You will be asked to complete this form each day during the last seven days of each two-month treatment period.

Day	Daily symptoms report
1	1. Did you take study medication yesterday? No ☐ Yes ☐<br>2. Did you experience muscle symptoms today? No ☐ Yes ☐ *If yes:*<br>3: Please indicate severity of muscle symptoms you experienced today.<br>0 1 2 3 4 5 6 7 8 9 10<br>No symptoms — Moderate symptoms — Worst possible symptoms
2	1. Did you take study medication yesterday? No ☐ Yes ☐<br>2. Did you experience muscle symptoms today? No ☐ Yes ☐ *If yes:*<br>3: Please indicate severity of muscle symptoms you experienced today.<br>0 1 2 3 4 5 6 7 8 9 10<br>No symptoms — Moderate symptoms — Worst possible symptoms
3	1. Did you take study medication yesterday? No ☐ Yes ☐<br>2. Did you experience muscle symptoms today? No ☐ Yes ☐ *If yes:*<br>3: Please indicate severity of muscle symptoms you experienced today.<br>0 1 2 3 4 5 6 7 8 9 10<br>No symptoms — Moderate symptoms — Worst possible symptoms

4	1. Did you take study medication yesterday? No ☐ Yes ☐<br>2. Did you experience muscle symptoms today? No ☐ Yes ☐<br>*If yes:*	3: Please indicate severity of muscle symptoms you experienced today.<br>0 1 2 3 4 5 6 7 8 9 10<br>No symptoms — Moderate symptoms — Worst possible symptoms
5	1. Did you take study medication yesterday? No ☐ Yes ☐<br>2. Did you experience muscle symptoms today? No ☐ Yes ☐<br>*If yes:*	3: Please indicate severity of muscle symptoms you experienced today.<br>0 1 2 3 4 5 6 7 8 9 10<br>No symptoms — Moderate symptoms — Worst possible symptoms
6	1. Did you take study medication yesterday? No ☐ Yes ☐<br>2. Did you experience muscle symptoms today? No ☐ Yes ☐<br>*If yes:*	3: Please indicate severity of muscle symptoms you experienced today.<br>0 1 2 3 4 5 6 7 8 9 10<br>No symptoms — Moderate symptoms — Worst possible symptoms
7	1. Did you take study medication yesterday? No ☐ Yes ☐<br>*If yes:*<br>2. Did you experience muscle symptoms today? No ☐ Yes ☐<br>*If yes:*	3: Please indicate severity of muscle symptoms you experienced today.<br>0 1 2 3 4 5 6 7 8 9 10<br>No symptoms — Moderate symptoms — Worst possible symptoms

Appendix 6: StatinWISE Patient Information Sheet

IRAS project ID: 197990

Site contact info and logo

StatinWISE: Investigation of **STATIN S**ide **E**ffects

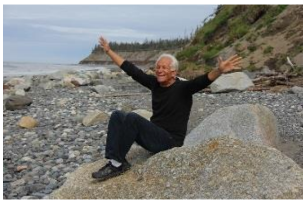
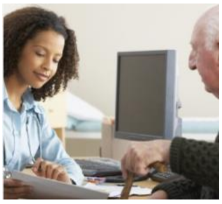
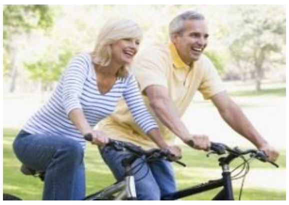
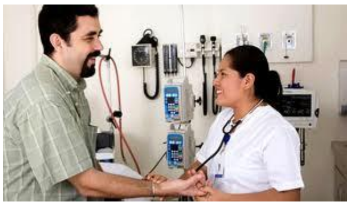

INFORMATION FOR PATIENTS

We invite you to take part in a research study called StatinWISE

- Before you decide whether to take part, it is important for you to understand why the research is being done, and what it will involve
- Please take time to read this information carefully and discuss it with friends or relatives if you wish
- You are encouraged to ask any questions you wish, before, during or after the study
- You are entirely free to decide whether or not to take part in this trial. If you choose not to take part, the care you are given by your GP will not be affected
- If there is anything that is not clear, or if you would like more information, please telephone the StatinWISE Research Nurse on [insert details]

Important things that you need to know

- We want to find out whether statins are the cause of muscle symptoms
- There will be only one visit to your GP practice as the study is done by phone or by the web
- The study treatment is posted directly to you and will fit through your letterbox
- At the end of the study, you will get personalised results so you can see if your own muscle symptoms are caused by statins
- You can stop taking part in the study at any time
- Support will be given to you throughout the study

Contents

1. Why are we doing this study?
2. Why have I been asked to take part?
3. What will happen if I take part?
4. What are the possible benefits of taking part in the study?
5. What are the possible risks of taking part in the study?
6. What if I don't want to be part of this study anymore?
7. What happens if I experience side effects?
8. Will my participation in the study be kept private?
9. Who can I contact to join the trial, ask questions or contact if I have a problem?
10. What else do you need to know?

1. Why are we doing this study?

Statins reduce the risk of heart attacks and strokes, however many patients stop taking statins because they get aches and pains in their muscles. The link between taking statins and aches and pains is not fully understood. Aches and pains are really common among people who don't take statins, and this means when someone taking a statin develops pain, it is really difficult to know whether the pain is caused by their statin. This means it is very difficult for patients and doctors to know whether to stop the statin or to continue. This is an important decision, because by not taking a statin, a patient's risk of a heart attack or stroke goes up by about one third. This study aims to determine whether statins cause muscle pain, thus allowing patients to make well-informed decisions whether to stop them or not.

2. Why have I been asked to take part?

You have been invited to take part because you have been prescribed a statin which you have either stopped taking within the last 3 years, or you are considering stopping, due to muscle related side-effects. Your GP thinks you are eligible and has invited you to take part. It is up to you to decide if you wish to take part or not.

3. What will happen if I take part?

Everyone taking part will have agreed to do so voluntarily, knowing the study involves:

- Most people will need a blood test, but this will only be once.
- One main visit to your GP Practice. The Research Nurse will explain everything about the study, including what to do if you have queries. You can ask any questions that you may have.
- If you decide to go ahead, you will have your weight, height and blood pressure recorded and sign a consent form.
- Taking atorvastatin or placebo (dummy treatment) daily for 1 year in 2-months sequences, switching between them in a random order. Neither you, nor your doctor, will know which you are taking in each 2-month period, except in an emergency.
- Completing 6 short questionnaires on your muscle symptoms every 2 months. There are a few ways in which you can choose to complete the questionnaires; via the web, verbally over the phone, mobile phone app or conventional paper form.
- At the end of 12 months, there will be one phone call or face-to-face appointment with your GP or Research Nurse to discuss your own results based on the information you provided in your questionnaires. You can also discuss the results with your GP and make a decision about whether you want to continue taking a statin or not.
- Three months after you stopped the study treatment, the study team will contact you to ask if you continued taking statins or not.

Study Timeline:

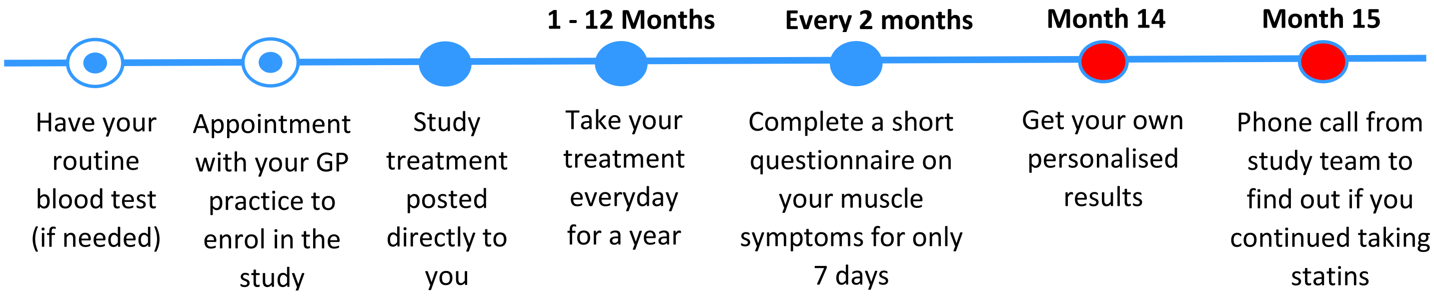

4. What are the possible benefits of taking part in the study?

This study will allow you and your GP to find out if any muscle symptoms you experience happen more when you are taking statins. This may help you to decide with your GP whether to take statins to reduce your risk of cardiovascular disease after the end of the study.

5. What are the possible risks of taking part in the study?

The study team do not know if statins cause milder muscle symptoms such as pain, which is why we are doing the study. Statins sometimes cause a very rare but serious muscle problem, but in less than 2 in 10,000 people.

6. What if I don't want to be part of this study anymore?

You can withdraw from the study at any time. We hope that you will let us use the information we have collected up to that point, but if you do not want us to use it please tell the Research Nurse or your GP.

7. What happens if I experience side effects?

You will be given an alert card when you join the study. If you experience any bad side effects, and wish to stop the medications, you should tell your GP. Your GP will continue to give you the best available care if there are any problems. If your GP decides you should not take part in the study for any reason, s/he can also withdraw you from the study.

8. Will my participation in the study be kept private?

The only people who are allowed to look at the information will be the team responsible for the study at the London School of Hygiene & Tropical Medicine and regulatory authorities who check that the study is being carried out properly. The GP Practice will need to share your personal information (name, address, phone number and email address) with the London School of Hygiene & Tropical Medicine so that they can send you the study treatment and help you with completing the questionnaires. All information collected about you will be kept private and used in strict confidence by the people working on the study.

We will publish the study results in a medical journal so that other doctors can benefit from the knowledge. Your personal information will not be included in the study report and there is no way that you can be identified. The study data, without any personal information, will be made available to researchers worldwide so that it can be used to improve medical knowledge and patient care.

9. Who can I contact to join the trial, ask questions or contact if I have a problem?

If you would like to join the study, you can complete the reply slip enclosed and return it in the freepost envelope provided. The Research Nurse at your GP Practice will then contact you to arrange an appointment. Otherwise you can call your GP Practice on [insert details] to arrange an appointment.

If you have any questions or concerns about the study, you should ask to speak with the study doctors or Research Nurses who will do their best to answer your questions. Your GP and Research Nurse can be contacted as follows:

Name	Personalise for each site
Address	
Phone Number	
Email	

10. What else do you need to know?

- The study is organised by the London School of Hygiene & Tropical Medicine and is funded by the Department of Health.
- The lead study doctor is a GP Professor Liam Smeeth at the London School of Hygiene and Tropical Medicine.
- In the unlikely event of you being harmed as a result of taking part in the study, the London School of Hygiene & Tropical Medicine would be responsible for claims for any non-negligent harm suffered as a result of participating in the study. You would retain the same rights of care as any other patient treated in the National Health Service.
- If you have any complaints about the StatinWISE study, please contact your GP Practice Manager.
- To protect your rights and well-being, all research conducted in the NHS is reviewed by an independent group of people called a Research Ethics Committee. Southampton Research Ethics Committee have given favorable ethical opinion for the study. The study has been reviewed, and approved, by the Medicines and Healthcare products Regulatory Agency who regulate clinical trials in the UK.
- If you would like to have a summary of the results of this study when it has ended, please let the Research Nurse know.

To contact the Clinical Trials Unit at London School of Hygiene & Tropical Medicine:

e-mail: statinwise@lshtm.ac.uk
Freephone: 0800 014 7410
Post: StatinWISE study, LSHTM, Room 180, Keppel Street, London WC1E 7HT.

Site contact info and logo

Appendix 6A: StatinWISE Patient Information Sheet – Optional Genetic Study

IRAS Project ID: 197990

Web-based **I**nvestigation of **STATIN S**ide **E**ffects

INVITATION TO TAKE PART IN AN OPTIONAL GENETIC STUDY FOR StatinWISE

A randomised clinical trial to assess if atorvastatin treatment causes more muscle symptoms than placebo

Thank you for taking part in StatinWISE

Before you decide whether to take part in this optional genetic study, it is important for you to understand why the research is being done and what is involved. Please take time to read the information carefully and discuss it with friends or relatives if you wish. You are entirely free to decide whether or not to take part in this optional blood sample study. There are no extra visits to take part in this study. If you choose not to take part, the care you are given by your GP will not be affected.

If anything is unclear, or if you would like more information, please telephone the StatinWISE Research Nurse on [insert details]

StatinWISE is coordinated by the London School of Hygiene & Tropical Medicine Clinical Trials Unit and funded by the National Institute for Health Research (NIHR) Health Technology Assessment (HTA) programme

Web-based Investigation of STATIN Side Effects

1. Why are we doing this optional genetic study

The link between taking statins and aches and pains is not fully understood, but may be linked to underlying genetic markers. This study aims to determine whether muscle pain considered to be due to statins is associated with genetic markers. Knowing this association may direct future medical management of using statins.

2. Why have I been asked to take part?

You have been asked to participate because you are taking part on StatinWISE. It is up to you to decide if you wish to participate or not. If you do not want to take part, your participation on StatinWISE will not change, neither will your future medical care be affected.

3. What will happen if I take part?

- Your GP practice research nurse will take one small (9mls, 2 teaspoons) blood sample
- Your sample will be labelled only with a trial identification number and sent to a laboratory at the University of Liverpool for analysis without any of your personal information.

4. What are the possible benefits of taking part in the study?

This optional study will allow scientists to understand if muscle symptoms experienced with statins are influenced by people's genes. The blood sample you give can be used also for other research studies. This may help direct future medical management of the use of statins.

5. What are the possible risks of taking part in the study?

It is very rare for a blood test to result in any serious complications. Risks associated with this test are no different to any regular blood test that you will have as part of routine clinical care. You may experience bruising as a result of taking the blood.

6. Will my participation in the study be kept private?

The only people who are allowed to look at your personal information will be the team responsible for the study at the London School of Hygiene & Tropical Medicine and regulatory authorities who check that the study is being carried out properly. The GP Practice will **not** share your personal information with the genetics laboratory. The results of the genetic analysis will not be shared with you, your GP or the StatinWISE team.

To contact the Clinical Trials Unit at London School of Hygiene & Tropical Medicine:

e-mail: statinwise@lshtm.ac.uk
Freephone: 0800 014 7410
Post: StatinWISE study, LSHTM, Room 180, Keppel Street, London WC1E 7HT.

Appendix 7: StatinWISE Informed Consent Form

Name of Principal Investigator:

1. Patient Initials				2. Patient Screening ID				-				3. Site ID			

Statement	Please initial each box
I confirm that I have read the information sheet dated…………………(version…….) for the above named study and given a copy to keep. I have had the opportunity to consider the information, ask questions and have these answered satisfactorily.	
I understand that my participation is voluntary and that I am free to withdraw at any time without giving any reason, and without my medical care or legal rights being affected.	
I understand that relevant sections of my medical notes and data collected during the study may be looked at by individuals from the sponsor of the trial (London School of Hygiene & Tropical Medicine) and responsible persons authorised by the sponsor, from ethics and regulatory authorities, or from the NHS Trust, where it is relevant to my taking part in this research. I give permission for these individuals to have access to my records.	
I understand that my personal details will be kept separately and I give permission for those details to be available to LSHTM Clinical Trial Unit staff to post the study treatment to my address.	
I understand that the information collected about me (with my personal information removed) will be used to support other research in the future, and I agree that data collected during this study can be used in future ethically approved research projects.	
I give permission for a copy of this consent form, which contains my personal information, to be made available to the LSHTM Clinical trials Unit.	
I agree to take part in the StatinWISE study.	

Printed name of participant	Signature of participant	Date

I confirm that I have explained the study information accurately to, and was understood to the best of my knowledge by, the participant and that he/she has freely given their consent to participate.

Printed name of person obtaining consent	Signature of person obtaining consent	Date

1 copy of participant, 1 for investigator file and 1 for medical notes.

Appendix 7A StatinWISE Informed Consent Form – Optional Genetic Study

Name of Principal Investigator:

1. Patient Initials				2. Patient Screening ID				-				3. Site ID			

Statement - Optional blood sample	Please initial each box
I understand that my blood sample will not contain any of my personal information and will be stored at the University of Liverpool.	
I give permission for my blood sample to be used in the future ethically approved research projects.	
I agree to have a blood sample taken for genetic analysis.	

Printed name of participant	Signature of participant	Date

I confirm that I have explained the study information accurately to, and was understood to the best of my knowledge by, the participant and that he/she has freely given their consent to participate.

Printed name of person obtaining consent	Signature of person obtaining consent	Date

1 copy of participant, 1 for investigator file and 1 for medical notes.

Appendix 8: Summary of Product Characteristics for Atorvastatin 20 mg tablets

1 NAME OF THE MEDICINAL PRODUCT

Atorvastatin 20 mg Film-coated Tablets

2 QUALITATIVE AND QUANTITATIVE COMPOSITION

Each film coated tablet contains 20 mg atorvastatin (as atorvastatin calcium)

For a full list of excipients, see section 6.1.

3 PHARMACEUTICAL FORM

Film-coated tablet.

White to off-white, elliptic, biconvex and smooth film-coated tablets. The dimensions of each tablet are approximately 12.5 mm x 6.6 mm.

4 CLINICAL PARTICULARS

4.1 THERAPEUTIC INDICATIONS

Hypercholesterolaemia

Atorvastatin is indicated as an adjunct to diet for reduction of elevated total cholesterol (total-C), LDL-cholesterol (LDL-C), apolipoprotein B and triglycerides in adults, adolescents and children aged 10 years or older with primary hypercholesterolaemia including familial hypercholesterolaemia (heterozygous variant) or combined (mixed) hyperlipidaemia (corresponding to Types IIa and IIb of the Fredrickson classification) when response to diet and other non pharmacological measures is inadequate.

Atorvastatin is also indicated to reduce total-C and LDL-C in adults with homozygous familial hypercholesterolaemia as an adjunct to other lipid-lowering treatments (e.g. LDL apheresis) or if such treatments are unavailable.

Prevention of cardiovascular disease

Prevention of cardiovascular events in patients estimated to have a high risk for a first cardiovascular event (see section 5.1), as an adjunct to correction of other risk factors.

4.2 POSOLOGY AND METHOD OF ADMINISTRATION

Posology

The patient should be placed on a standard cholesterol-lowering diet before receiving atorvastatin and should continue on this diet during treatment with atorvastatin.

The dose should be individualised according to baseline LDL-C levels, the goal of therapy, and patient response.

The usual starting dose is 10 mg once a day. Adjustment of dose should be made at intervals of 4 weeks or more. The maximum dose is 80 mg once a day.

Primary hypercholesterolaemia and combined (mixed) hyperlipidaemia The majority of patients are controlled with atorvastatin 10 mg once a day. A therapeutic response is evident within 2 weeks, and the maximum therapeutic response is usually achieved within 4 weeks. The response is maintained during chronic therapy.

Heterozygous familial hypercholesterolaemia

Patients should be started with atorvastatin 10 mg daily. Doses should be individualised and adjusted every 4 weeks to 40 mg daily. Thereafter, either the dose may be increased to a maximum of 80 mg daily or a bile acid sequestrant may be combined with 40 mg atorvastatin once daily.

Homozygous familial hypercholesterolaemia

Only limited data are available (see section 5.1).

The dose of atorvastatin in patients with homozygous familial hypercholesterolaemia is 10 to 80 mg daily (see section 5.1). Atorvastatin should be used as an adjunct to other lipid-lowering treatments (e.g. LDL apheresis) in these patients or if such treatments are unavailable.

Prevention of cardiovascular disease

In the primary prevention trials the dose was 10 mg/day. Higher dosages may be necessary in order to attain (LDL-) cholesterol levels according to current guidelines.

Renal impairment

No adjustment of dose is required (see section 4.4).

Hepatic impairment

Atorvastatin should be used with caution in patients with hepatic impairment (see sections 4.4 and 5.2). Atorvastatin is contraindicated in patients with active liver disease (see section 4.3).

Use in the elderly

Efficacy and safety in patients older than 70 using recommended doses are similar to those seen in the general population.

Paediatric use

Hypercholesterolaemia:

Paediatric use should only be carried out by physicians experienced in the treatment of paediatric hyperlipidaemia and patients should be re-evaluated on a regular basis to assess progress.

For patients aged 10 years and above, the recommended starting dose of atorvastatin is 10 mg per day with titration up to 20 mg per day. Titration should be conducted according to the individual response and tolerability in paediatric patients. Safety information for paediatric patients treated with doses above 20 mg, corresponding to about 0.5 mg/kg, is limited.

There is limited experience in children between 6-10 years of age (see section 5.1). Atorvastatin is not indicated in the treatment of patients below the age of 10 years.

Other pharmaceutical forms/strengths may be more appropriate for this population.

Method of administration

Atorvastatin is for oral administration. Each daily dose of atorvastatin is given all at once and may be given at any time of day with or without food.

4.3 CONTRAINDICATIONS

Atorvastatin is contraindicated in patients:

- with hypersensitivity to the active substance or to any of the excipients of this medicinal product
- with active liver disease or unexplained persistent elevations of serum transaminases exceeding 3 times the upper limit of normal
- during pregnancy, while breast-feeding and in women of child-bearing potential not using appropriate contraceptive measures (see section 4.6).

4.4 SPECIAL WARNINGS AND PRECAUTIONS FOR USE

Liver effects

Liver function tests should be performed before the initiation of treatment and periodically thereafter. Patients who develop any signs or symptoms suggestive of liver injury should have liver function tests performed. Patients who develop increased transaminase levels should be monitored until the abnormality(ies) resolve. Should an increase in transaminases of greater than 3 times the upper limit of normal (ULN) persist, reduction of dose or withdrawal of atorvastatin is recommended (see section 4.8).

Atorvastatin should be used with caution in patients who consume substantial quantities of alcohol and/or have a history of liver disease.

Stroke Prevention by Aggressive Reduction in Cholesterol Levels (SPARCL) In a *post-hoc* analysis of stroke subtypes in patients without coronary heart disease (CHD) who had a recent stroke or transient ischemic attack (TIA) there was a higher incidence of haemorrhagic stroke in patients initiated on atorvastatin 80 mg compared to placebo. The increased risk was particularly noted in patients with prior haemorrhagic stroke or lacunar infarct at study entry. For patients with prior haemorrhagic stroke or lacunar infarct, the balance of risks and benefits of atorvastatin 80 mg is uncertain, and the potential risk of haemorrhagic stroke should be carefully considered before initiating treatment (see section 5.1). Immune-mediated necrotizing myopathy (IMNM) There have been very rare reports of an immune-mediated necrotizing myopathy (IMNM) during or after treatment with some statins. IMNM is clinically characterized by persistent proximal muscle weakness and elevated serum creatine kinase, which persist despite discontinuation of statin treatment.

Skeletal muscle effects

Atorvastatin, like other HMG-CoA reductase inhibitors, may in rare occasions affect the skeletal muscle and cause myalgia, myositis, and myopathy that may progress to rhabdomyolysis, a potentially life-threatening condition characterised by markedly elevated creatine kinase (CK) levels (>10 times ULN), myoglobinaemia and myoglobinuria which may lead to renal failure.

Before the treatment

Atorvastatin should be prescribed with caution in patients with pre-disposing factors for rhabomyolysis. A CK level should be measured before starting statin treatment in the following situations:

− Renal impairment
− Hypothyroidism

- − Personal or familial history of hereditary muscular disorders
- − Previous history of muscular toxicity with a statin or fibrate
- − Previous history of liver disease and/or where substantial quantities of alcohol are consumed
- − In the elderly (age >70 years), the necessity of such measurement should be considered, according to the presence of other pre-disposing factors for rhabdomyolysis.
- − Situations where an increase in plasma levels may occur, such as interactions (see section 4.5) and special populations including genetic subpopulations (see section 5.2)

In such situations, the risk of treatment should be considered in relation to possible benefit, and clinical monitoring is recommended.

If CK levels are significantly elevated (>5 times ULN) at baseline, treatment should not be started.

Creatine kinase measurement

Creatine kinase (CK) should not be measured following strenuous exercise or in the presence of any plausible alternative cause of CK increase as this makes value interpretation difficult. If CK levels are significantly elevated at baseline (>5 times ULN), levels should be remeasured within 5 to 7 days later to confirm the results.

Whilst on treatment

- − Patients must be asked to promptly report muscle pain, cramps, or weakness especially if accompanied by malaise or fever.
- − If such symptoms occur whilst a patient is receiving treatment with atorvastatin, their CK levels should be measured. If these levels are found to be significantly elevated (>5 times ULN), treatment should be stopped.
- − If muscular symptoms are severe and cause daily discomfort, even if the CK levels are elevated to ≤5 x ULN, treatment discontinuation should be considered.
- − If symptoms resolve and CK levels return to normal, then re-introduction of atorvastatin or introduction of an alternative statin may be considered at the lowest dose and with close monitoring.
- − Atorvastatin must be discontinued if clinically significant elevation of CK levels (>10 x ULN) occur, or if rhabdomyolysis is diagnosed or suspected.

<u>Concomitant treatment with other medicinal products</u>

Risk of rhabdomyolysis is increased when atorvastatin is administered concomitantly with certain medicinal products that may increase the plasma concentration of atorvastatin such as potent inhibitors of CYP3A4 or transport proteins (e.g.
ciclosporine, telithromycin, clarithromycin, delavirdine, stiripentol, ketoconazole, voriconazole, itraconazole, posaconazole and HIV protease inhibitors including ritonavir, lopinavir, atazanavir, indinavir, darunavir, etc). The risk of myopathy may also be increased with the concomitant use of gemfibrozil and other fibric acid derivates, erythromycin, niacin and ezetimibe. If possible, alternative (noninteracting) therapies should be considered instead of these medicinal products.

In cases where co-administration of these medicinal products with atorvastatin is necessary, the benefit and the risk of concurrent treatment should be carefully considered. When patients are receiving medicinal products that increase the plasma concentration of atorvastatin, a lower maximum dose of atorvastatin is recommended. In addition, in the case of potent CYP3A4 inhibitors, a lower starting dose of atorvastatin should be considered and appropriate clinical monitoring of these patients is recommended (see section 4.5).

The concurrent use of atorvastatin and fusidic acid is not recommended, therefore, temporary suspension of atorvastatin may be considered during fusidic acid therapy (see section 4.5).

<u>Interstitial lung disease</u>

Exceptional cases of interstitial lung disease have been reported with some statins, especially with long term therapy (see section 4.8). Presenting features can include dyspnoea, non productive cough and deterioration in general health (fatigue, weight loss and fever). If it is suspected a patient has developed interstitial lung disease, statin therapy should be discontinued.

Diabetes Mellitus
Some evidence suggests that statins as a class raise blood glucose and in some patients, at high risk of future diabetes, may produce a level of hyperglycaemia where formal diabetes care is appropriate. This risk, however, is outweighed by the reduction in vascular risk with statins and therefore should not be a reason for stopping statin treatment. Patients at risk (fasting glucose 5.6 to 6.9 mmol/L, BMI>30kg/m^2, raised triglycerides, hypertension) should be monitored both clinically and biochemically according to national guidelines.

Paediatric use

Developmental safety in the paediatric population has not been established (see section 4.8).

4.5 INTERACTION WITH OTHER MEDICINAL PRODUCTS AND OTHER FORMS OF INTERACTION

Effect of co-administered medicinal products on atorvastatin

Atorvastatin is metabolized by cytochrome P450 3A4 (CYP3A4) and is a substrate to transport proteins e.g. the hepatic uptake transporter OATP1B1. Concomitant administration of medicinal products that are inhibitors of CYP3A4 or transport proteins may lead to increased plasma concentrations of atorvastatin and an increased risk of myopathy. The risk might also be increased at concomitant administration of atorvastatin with other medicinal products that have a potential to induce myopathy, such as fibric acid derivates and ezetimibe (see section 4.4).

CYP3A4 inhibitors

Potent CYP3A4 inhibitors have been shown to lead to markedly increased concentrations of atorvastatin (see Table 1 and specific information below). Coadministration of potent CYP3A4 inhibitors (e.g. ciclosporin, telithromycin, clarithromycin, delavirdine, stiripentol, ketoconazole, voriconazole, itraconazole, posaconazole and HIV protease inhibitors including ritonavir, lopinavir, atazanavir, indinavir, darunavir, etc.) should be avoided if possible. In cases where coadministration of these medicinal products with atorvastatin cannot be avoided lower starting and maximum doses of atorvastatin should be considered and appropriate clinical monitoring of the patient is recommended (see Table 1).

Moderate CYP3A4 inhibitors (e.g. erythromycin, diltiazem, verapamil and fluconazole) may increase plasma concentrations of atorvastatin (see Table 1).. An increased risk of myopathy has been observed with the use of erythromycin in combination with statins. Interaction studies evaluating the effects of amiodarone or verapamil on atorvastatin have not been conducted. Both amiodarone and verapamil are known to inhibit CYP3A4 activity and co-administration with atorvastatin may result in increased exposure to atorvastatin. Therefore, a lower maximum dose of atorvastatin should be considered and appropriate clinical monitoring of the patient is recommended when concomitantly used with moderate CYP3A4 inhibitors. Appropriate clinical monitoring is recommended after initiation or following dose adjustments of the inhibitor.

CYP3A4 inducers

Concomitant administration of atorvastatin with inducers of cytochrome P450 3A (e.g. efavirenz, rifampin, St. John's Wort) can lead to variable reductions in plasma concentrations of atorvastatin. Due to the dual interaction mechanism of rifampin, (cytochrome P450 3A induction and inhibition of hepatocyte uptake transporter OATP1B1), simultaneous co-administration of atorvastatin with rifampin is recommended, as delayed administration of atorvastatin after administration of rifampin has been associated with a significant reduction in atorvastatin plasma concentrations. The effect of rifampin on atorvastatin concentrations in hepatocytes is, however, unknown and if concomitant administration cannot be avoided, patients should be carefully monitored for efficacy.

Transport protein inhibitors

Inhibitors of transport proteins (e.g. ciclosporin) can increase the systemic exposure of atorvastatin (see Table 1). The effect of inhibition of hepatic uptake transporters on atorvastatin concentrations in hepatocytes is unknown. If concomitant administration cannot be avoided, a dose reduction and clinical monitoring for efficacy is recommended (see Table 1).

Gemfibrozil / fibric acid derivatives

The use of fibrates alone is occasionally associated with muscle related events, including rhabdomyolysis. The risk of these events may be increased with the concomitant use of fibric acid derivatives and atorvastatin. If concomitant administration cannot be avoided, the lowest dose of atorvastatin to achieve the therapeutic objective should be used and the patients should be appropriately monitored (see section 4.4).

Ezetimibe

The use of ezetimibe alone is associated with muscle related events, including rhabdomyolysis. The risk of these events may therefore be increased with concomitant use of ezetimibe and atorvastatin. Appropriate clinical monitoring of these patients is recommended.

Colestipol

Plasma concentrations of atorvastatin and its active metabolites were lower (by approx. 25%) when colestipol was co-administered with atorvastatin. However, lipid effects were greater when atorvastatin and colestipol were co-administered than when either medicinal product was given alone.

Fusidic acid

Interaction studies with atorvastatin and fusidic acid have not been conducted. As with other statins, muscle related events, including rhabdomyolysis, have been reported in post-marketing experience with atorvastatin and fusidic acid given concurrently. The mechanism of this interaction is not known. Patients should be closely monitored and temporary suspension of atorvastatin treatment may be appropriate.

Effect of atorvastatin on co-administered medicinal products

Digoxin

When multiple doses of digoxin and 10 mg atorvastatin were co-administered, steadystate digoxin concentrations increased slightly. Patients taking digoxin should be monitored appropriately.

Oral contraceptives

Co-administration of atorvastatin with an oral contraceptive produced increases in plasma concentrations of norethindrone and ethinyl oestradiol.

Warfarin

In a clinical study in patients receiving chronic warfarin therapy, coadministration of atorvastatin 80 mg daily with warfarin caused a small decrease of about 1.7 seconds in prothrombin time during the first 4 days of dosing which returned to normal within 15 days of atorvastatin treatment. Although only very rare cases of clinically significant anticoagulant interactions have been reported, prothrombin time should be determined before starting atorvastatin in patients taking coumarin anticoagulants and frequently enough during early therapy to ensure that no significant alteration of prothrombin time occurs. Once a stable prothrombin time has been documented, prothrombin times can be monitored at the intervals usually recommended for patients on coumarin anticoagulants. If the dose of atorvastatin is changed or discontinued, the same procedure should be repeated. Atorvastatin therapy has not been associated with bleeding or with changes in prothrombin time in patients not taking anticoagulants.

Paediatric population

Drug-drug interaction studies have only been performed in adults. The extent of interactions in the paediatric population is not known. The above mentioned interactions for adults and the warnings in section 4.4 should be taken into account for the paediatric population.

Table 1: Effect of co-administered medicinal products on the pharmacokinetics of atorvastatin

Co-administered medicinal product and dosing regimen	Atorvastatin		
	Dose (mg)	Change in AUC*	Clinical Recommendation**
Tipranavir 500 mg BID/ Ritonavir 200 mg BID, 8 days (days 14 to 21)	40 mg on day 1, 10 mg on day 20	↑ 9.4 fold	In cases where coadministration with atorvastatin is necessary, do not exceed 10 mg atorvastatin daily. Clinical monitoring of these patients is recommended
Ciclosporin 5.2 mg/kg/day, stable dose	10 mg OD for 28 days	↑ 8.7 fold	
Lopinavir 400 mg BID/ Ritonavir 100 mg BID, 14 days	20 mg OD for 4 days	↑ 5.9 fold	In cases where coadministration with atorvastatin is necessary, lower maintenance doses of atorvastatin are recommended. At atorvastatin doses exceeding 20 mg, clinical monitoring of these patients is recommended.
Clarithromycin 500 mg BID, 9 days	80 mg OD for 8 days	↑ 4.4 fold	
Saquinavir 400 mg BID/ Ritonavir (300 mg BID from days 5-7, increased to 400 mg BID on day 8), days 5-18, 30 min after atorvastatin dosing	40 mg OD for 4 days	↑ 3.9 fold	In cases where coadministration with atorvastatin is necessary, lower maintenance doses of atorvastatin are recommended. At atorvastatin doses exceeding 40 mg, clinical monitoring of these patients is recommended.
Darunavir 300 mg BID/ Ritonavir 100 mg BID, 9 days	10 mg OD for 4 days	↑ 3.3 fold	
Itraconazole 200 mg OD, 4 days	40 mg SD	↑ 3.3 fold	
Fosamprenavir 700 mg BID/ Ritonavir 100 mg BID, 14 days	10 mg OD for 4 days	↑ 2.5 fold	
Fosamprenavir 1400 mg BID, 14 days	10 mg OD for 4 days	↑ 2.3 fold	
Nelfinavir 1250 mg BID, 14 days	10 mg OD for 28 days	↑ 1.7 fold***	No specific recommendation

Grapefruit Juice, 240 mL OD ****	40 mg, SD	↑ 37%	Concomitant intake of large quantities of grapefruit juice and atorvastatin is not recommended.
Diltiazem 240 mg OD, 28 days	40 mg, SD	↑ 51%	After initiation or following dose adjustments of diltiazem, appropriate clinical monitoring of these patients is recommended.
Erythromycin 500 mg QID, 7 days	10 mg, SD	↑ 33%***	Lower maximum dose and clinical monitoring of these patients is recommended.
Amlodipine 10 mg, single dose	80 mg, SD	↑ 18%	No specific recommendation.
Cimetidine 300 mg QID, 2 weeks	10 mg OD for 4 weeks	↓ less than 1%***	No specific recommendation.
Antacid suspension of magnesium and aluminium hydroxides, 30 mL QID, 2 weeks	10 mg OD for 4 weeks	↓ 35%***	No specific recommendation.
Efavirenz 600 mg OD, 14 days	10 mg for 3 days	↓ 41%	No specific recommendation.
Rifampin 600 mg OD, 7 days (coadministered)	40 mg SD	↑ 30%	If co-administration cannot be avoided, simultaneous coadministration of atorvastatin with rifampin is recommended, with clinical monitoring.
Rifampin 600 mg OD, 5 days (doses separated)	40 mg SD	↓ 80%	
Gemfibrozil 600 mg BID, 7 days	40mg SD	↑ 35%	Lower starting dose and clinical monitoring of these patients is recommended.
Fenofibrate 160 mg OD, 7 days	40mg SD	↑ 3%	Lower starting dose and clinical monitoring of these patients is recommended.

* Data given as x-fold change represent a simple ratio between co-administration and atorvastatin alone (i.e., 1-fold = no change). Data given as % change represent % difference relative to atorvastatin alone (i.e., 0% = no change).
** See sections 4.4 and 4.5 for clinical significance.
*** Total atorvastatin equivalent activity
**** Contains one or more components that inhibit CYP3A4 and can increase plasma concentrations of medicinal products metabolized by CYP3A4. Intake of one 240 ml glass of grapefruit juice also resulted in a decreased AUC of 20.4% for the active orthohydroxy metabolite. Large quantities of grapefruit juice (over 1.2 l daily for 5 days) increased AUC of atorvastatin 2.5 fold and AUC of active (atorvastatin and metabolites).

Increase is indicated as “↑”, decrease as “↓”
OD = once daily; SD = single dose; BID = twice daily; QID = four times daily

Table 2: Effect of atorvastatin on the pharmacokinetics of co-administered medicinal products

Atorvastatin and dosing regimen	Co-administered medicinal product		
	Medicinal product/Dose (mg)	Change in AUC*	Clinical Recommendation
80 mg OD for 10 days	Digoxin 0.25 mg OD, 20 days	↑ 15%	Patients taking digoxin should be monitored appropriately.
40 mg OD for 22 days	Oral contraceptive OD, 2 months<br>- norethindrone 1 mg - ethinyl estradiol 35 µg	↑ 28%<br>↑ 19%	No specific recommendation.
80 mg OD for 15 days	** Phenazone, 600 mg SD	↑ 3%	No specific recommendation.

* Data given as % change represent % difference relative to atorvastatin alone (i.e., 0% = no change)
** Co-administration of multiple doses of atorvastatin and phenazone showed little or no detectable effect in the clearance of phenazone.

Increase is indicated as "↑", decrease as "↓"
OD = once daily; SD = single dose

4.6 PREGNANCY AND LACTATION

Women of childbearing potential

Women of child-bearing potential should use appropriate contraceptive measures during treatment (see section 4.3).

Pregnancy

Atorvastatin is contraindicated during pregnancy (see section 4.3). Safety in pregnancy woman has not been established (see section 4.3). No controlled clinical trials with atorvastatin have been conducted in pregnant women. Rare reports of congenital anomalies following intrauterine exposure to HMG-CoA reductase inhibitors have been received. Animal studies have shown toxicity to reproduction (see section 5.3).

Maternal treatment with atorvastatin may reduce the fetal levels of mevalonate which is a precursor of cholesterol biosynthesis. Atherosclerosis is a chronic process, and ordinarily discontinuation of lipid-lowering medicinal products during pregnancy should have little impact on the long-term risk associated with primary hypercholesterolaemia.

For these reasons, atorvastatin should not be used in women who are pregnant, trying to become pregnant or suspect they are pregnant. Treatment with atorvastatin should be suspended for the duration of pregnancy or until it has been determined that the woman is not pregnant (see section 4.3.)

Breastfeeding

It is not known whether atorvastatin or its metabolites are excreted in human milk. In rats, plasma concentrations of atorvastatin and its active metabolites are similar to those in milk (see section 5.3). Because of the potential for serious adverse reactions, women taking atorvastatin should not breast-feed their infants (see section 4.3). Atorvastatin is contraindicated during breastfeeding (see section 4.3).

Fertility

In animal studies atorvastatin had no effect on male or female fertility (see section 5.3).

4.7 EFFECTS ON ABILITY TO DRIVE AND USE MACHINES

Atorvastatin has negligible influence on the ability to drive and use machines.

4.8 UNDESIRABLE EFFECTS

In the atorvastatin placebo-controlled clinical trial database of 16,066 (8755 Lipitor vs. 7311 placebo) patients treated for a mean period of 53 weeks, 5.2% of patients on atorvastatin discontinued due to adverse reactions compared to 4.0% of the patients on placebo.

Based on data from clinical studies and extensive post-marketing experience, the following table presents the adverse reaction profile for atorvastatin.

Estimated frequencies of reactions are ranked according to the following convention: common (≥1/100, <1/10); uncommon (≥1/1,000, <1/100); rare (≥1/10,000, <1/1,000); very rare (≤1/10,000), not known (cannot be estimated from the available data).

<u>Infections and infestations</u>	
Common:	nasopharyngitis.
<u>Blood and lymphatic system disorders</u>	
Rare:	thrombocytopenia.
<u>Immune system disorders</u>	
Common:	allergic reactions
Very rare:	anaphylaxis
<u>Metabolism and nutrition disorders</u>	
Common:	hyperglycaemia
Uncommon:	hypoglycaemia, weight gain, anorexia
<u>Psychiatric disorders</u>	
Uncommon:	nightmare, insomnia
<u>Nervous system disorders</u>	
Common:	headache
Uncommon:	dizziness, paraesthesia, hypoaesthesia, dysgeusia, amnesia
Rare:	peripheral neuropathy
<u>Eye disorders</u>	
Uncommon:	vision blurred
Rare:	visual disturbance
<u>Ear and labyrinth disorders</u>	
Uncommon:	tinnitus
Very rare:	hearing loss
<u>Respiratory, thoracic and mediastinal disorders</u>	
Common:	pharyngolaryngeal pain, epistaxis
<u>Gastrointestinal disorders</u>	
Common:	constipation, flatulence, dyspepsia, nausea, diarrhoea
Uncommon:	vomiting, abdominal pain upper and lower, eructation, pancreatitis
<u>Hepatobiliary disorders</u>	
Uncommon:	Hepatitis
Rare:	cholestasis
Very rare:	hepatic failure
<u>Skin and subcutaneous tissue disorders</u>	
Uncommon:	urticaria, skin rash, pruritus, alopecia
Rare:	angioneurotic oedema, dermatitis, bullous including erythema multiforme, Stevens-Johnson syndrome and toxic epidermal necrolysis
<u>Musculoskeletal and connective tissue disorders</u>	
Common:	myalgia, arthralgia, pain in extremity, muscle spasms, joint swelling, back pain
Uncommon:	neck pain, muscle fatigue
Rare:	myopathy, myositis, rhabdomyolysis, tendonopathy, sometimes complicated by rupture
Not known:	immune-mediated necrotizing myopathy (see section 4.4)

Reproductive system and breast disorders	
Very Rare:	gynaecomastia
General disorders and administration site conditions	
Uncommon:	malaise, asthenia, chest pain, peripheral oedema, fatigue, pyrexia.
Investigations	
Common:	liver function test abnormal, blood creatine kinase increased
Uncommon:	white blood cells urine positive

As with other HMG-CoA reductase inhibitors elevated serum transaminases have been reported in patients receiving atorvastatin. These changes were usually mild, transient, and did not require interruption of treatment. Clinically important (>3 times upper normal limit) elevations in serum transaminases occurred in 0.8% patients on atorvastatin. These elevations were dose-related and were reversible in all patients.

Elevated serum creatine kinase (CK) levels greater than 3 times upper limit of normal occurred in 2.5% of patients on atorvastatin, similar to other HMG-CoA reductase inhibitors in clinical trials. Levels above 10 times the normal upper range occurred in 0.4% atorvastatin-treated patients (see section 4.4).

The following adverse events have been reported with some statins:

- Sexual dysfunction
- Depression
- Exceptional cases of interstitial lung disease, especially with long term therapy (see section 4.4)
- Diabetes Mellitus: Frequency will depend on the presence or absence of risk factors (fasting blood glucose ≥ 5.6 mmol/L, BMI>30kg/m^2, raised triglycerides, history of hypertension).

Paediatric Population

The clinical safety database includes safety data for 249 paediatric patients who received atorvastatin, among which 7 patients were < 6 years old, 14 patients were in the age range of 6 to 9, and 228 patients were in the age range of 10 to 17.

Nervous system disorders

Common: Headache

Gastrointestinal disorders

Common: Abdominal pain

Investigations

Common: Alanine aminotransferase increased, blood creatine phosphokinase increased

Based on the data available, frequency, type and severity of adverse reactions in children are expected to be the same as in adults. There is currently limited experience with respect to long-term safety in the paediatric population.

Reporting of side effects
If you get side effects, talk to your doctor or pharmacist. This includes any possible side effects not listed in this leaflet.

You can also report side effects directly via the Yellow Card Scheme at:
www.mhra.gov.uk/yellowcard

By reporting side effects you can help provide more information on the safety of this medicine.

4.9 OVERDOSE

Specific treatment is not available for atorvastatin overdose. Should an overdose occur, the patient should be treated symptomatically and supportive measures instituted, as required. Liver function tests should be performed and serum CK levels should be monitored. Due to extensive atorvastatin binding to plasma proteins, haemodialysis is not expected to significantly enhance atorvastatin clearance.

5 PHARMACOLOGICAL PROPERTIES

5.1 PHARMACODYNAMIC PROPERTIES

Pharmacotherapeutic group: Lipid modifying agents, HMG-CoA reductase inhibitors
ATC code: C10A A05

Atorvastatin is a selective, competitive inhibitor of HMG-CoA reductase, the ratelimiting enzyme responsible for the conversion of 3-hydroxy-3-methyl-glutarylcoenzyme A to mevalonate, a precursor of sterols, including cholesterol. Triglycerides and cholesterol in the liver are incorporated into very low-density lipoproteins (VLDL) and released into the plasma for delivery to peripheral tissues. Low-density lipoprotein (LDL) is formed from VLDL and is catabolised primarily through the receptor with high affinity to LDL (LDL receptor).

Atorvastatin lowers plasma cholesterol and lipoprotein serum concentrations by inhibiting HMG-CoA reductase and subsequently cholesterol biosynthesis in the liver and increases the number of hepatic LDL receptors on the cell surface for enhanced uptake and catabolism of LDL.

Atorvastatin reduces LDL production and the number of LDL particles. Atorvastatin produces a profound and sustained increase in LDL receptor activity coupled with a beneficial change in the quality of circulating LDL particles. Atorvastatin is effective in reducing LDL-C in patients with homozygous familial hypercholesterolaemia, a population that has not usually responded to lipid-lowering agents.

Atorvastatin has been shown to reduce concentrations of total-C (30%-46%), LDL-C (41%-61%), apolipoprotein B (34%-50%), and triglycerides (14%-33%) while producing variable increases in HDL-C and apolipoprotein A1 in a dose-response study. These results are consistent in patients with heterozygous familial hypercholesterolaemia, non-familial forms of hypercholesterolaemia, and mixed hyperlipidaemia, including patients with noninsulin-dependent diabetes mellitus.

Reductions in total-C, LDL-C, and apolipoprotein B have been proven to reduce the risk for cardiovascular events and cardiovascular mortality.

Homozygous familial hypercholesterolaemia

In a multicenter 8 week open-label compassionate-use study with an optional extension phase of variable length, 335 patients were enrolled, 89 of which were identified as homozygous familial hypercholesterolaemia patients. From these 89 patients, the mean percent reduction in LDL-C was approximately 20%. Atorvastatin was administered at doses up to 80 mg/day.

Atherosclerosis

In the Reversing Atherosclerosis with Aggressive Lipid- Lowering Study (REVERSAL), the effect of intensive lipid lowering with atorvastatin 80 mg and standard degree of lipid lowering with pravastatin 40 mg on coronary atherosclerosis was assessed by intravascular ultrasound (IVUS), during angiography, in patients with coronary heart disease. In this randomised, double- blind, multicenter, controlled clinical trial, IVUS was performed at baseline and at 18 months in 502 patients. In the atorvastatin group (n=253), there was no progression of atherosclerosis.

The median percent change, from baseline, in total atheroma volume (the primary study criteria) was -0.4% (p=0.98) in the atorvastatin group and +2.7% (p=0.001) in the pravastatin group (n=249). When compared to pravastatin the effects of atorvastatin were statistically significant (p=0.02). The effect of intensive lipid lowering on cardiovascular endpoints (e. g. need for revascularisation, non fatal myocardial infarction, coronary death) was not investigated in this study.

In the atorvastatin group, LDL-C was reduced to a mean of 2.04 mmol/L ± 0.8 (78.9 mg/dl ± 30) from baseline 3.89 mmol/l ± 0.7 (150 mg/dl ± 28) and in the pravastatin group, LDL-C was reduced to a mean of 2.85 mmol/l ± 0.7 (110 mg/dl ± 26) from baseline 3.89 mmol/l ± 0.7 (150 mg/dl ± 26) (p<0.0001). Atorvastatin also significantly reduced mean TC by 34.1% (pravastatin: -18.4%, p<0.0001), mean TG levels by 20% (pravastatin: -6.8%, p<0.0009), and mean apolipoprotein B by 39.1% (pravastatin: -22.0%, p<0.0001). Atorvastatin increased mean HDL-C by 2.9% (pravastatin: +5.6%, p=NS). There was a 36.4% mean reduction in CRP in the atorvastatin group compared to a 5.2% reduction in the pravastatin group (p<0.0001).

Study results were obtained with the 80 mg dose strength. Therefore, they cannot be extrapolated to the lower dose strengths.

The safety and tolerability profiles of the two treatment groups were comparable.

The effect of intensive lipid lowering on major cardiovascular endpoints was not investigated in this study. Therefore, the clinical significance of these imaging results with regard to the primary and secondary prevention of cardiovascular events is unknown.

<u>Acute coronary syndrome</u>

In the MIRACL study, atorvastatin 80 mg has been evaluated in 3,086 patients (atorvastatin n=1,538; placebo n=1,548) with an acute coronary syndrome (non Qwave MI or unstable angina). Treatment was initiated during the acute phase after hospital admission and lasted for a period of 16 weeks. Treatment with atorvastatin 80 mg/day increased the time to occurrence of the combined primary endpoint, defined as death from any cause, nonfatal MI, resuscitated cardiac arrest, or angina pectoris with evidence of myocardial ischaemia requiring hospitalization, indicating a risk reduction by 16% (p=0.048). This was mainly due to a 26% reduction in rehospitalisation for angina pectoris with evidence of myocardial ischaemia (p=0.018). The other secondary endpoints did not reach statistical significance on their own (overall: Placebo: 22.2%, Atorvastatin: 22.4%).

The safety profile of atorvastatin in the MIRACL study was consistent with what is described in section 4.8.

<u>Prevention of cardiovascular disease</u>

The effect of atorvastatin on fatal and non-fatal coronary heart disease was assessed in a randomised, double-blind, placebo-controlled study, the Anglo-Scandinavian Cardiac Outcomes Trial Lipid Lowering Arm (ASCOT-LLA). Patients were hypertensive, 40-79 years of age, with no previous myocardial infarction or treatment for angina, and with TC levels ≤6.5 mmol/L (251 mg/dL). All patients had at least 3 of the predefined cardiovascular risk factors: male gender, age ≥55 years, smoking, diabetes, history of CHD in a first-degree relative, TC:HDL-C >6, peripheral vascular disease, left ventricular hypertrophy, prior cerebrovascular event, specific ECG abnormality, proteinuria/albuminuria. Not all included patients were estimated to have a high risk for a first cardiovascular event.

Patients were treated with antihypertensive therapy (either amlodipine or atenololbased regimen) and either atorvastatin 10 mg daily (n=5,168) or placebo (n=5,137).

The absolute and relative risk reduction effect of atorvastatin was as follows:

Event	*Relative risk reduction (%)*	*No. of events (atorvastatin vs placebo)*	*Absolute risk reduction[1](%)*	*P value*
Fatal CHD plus non-fatal MI	36%	100 vs. 154	1.1%	0.0005
Total cardiovascular events and revascularisation procedures	20%	389 vs. 483	1.9%	0.0008
Total coronary events	29%	178 vs 247	1.4%	0.0006

[1] *Based on difference in crude events rates occurring over a median follow-up of 3.3 years.*
CHD = coronary heart disease; MI = myocardial infarction.

Total mortality and cardiovascular mortality were not significantly reduced (185 vs. 212 events, p=0.17 and 74 vs. 82 events, p=0.51). In the subgroup analyses by gender (81% males, 19% females), a beneficial effect of atorvastatin was seen in males but could not be established in females possibly due to the low event rate in the female subgroup. Overall and cardiovascular mortality were numerically higher in the female patients (38 vs. 30 and 17 vs. 12), but this was not statistically significant. There was significant treatment interaction by antihypertensive baseline therapy. The primary endpoint (fatal CHD plus non-fatal MI) was significantly reduced by atorvastatin in patients treated with amlodipine (HR 0.47 (0.32-0.69), p=0.00008), but not in those treated with atenolol (HR 0.83 (0.59-1.17), p=0.287).

The effect of atorvastatin on fatal and non-fatal cardiovascular disease was also assessed in a randomised, double-blind, multicentre, placebo-controlled trial, the Collaborative Atorvastatin Diabetes Study (CARDS) in patients with type 2 diabetes,
40-75 years of age, without prior history of cardiovascular disease, and with LDL-C ≤4.14 mmol/L (160 mg/dL) and TG ≤6.78 mmol/L (600 mg/dL). All patients had at least 1 of the following risk factors: hypertension, current smoking, retinopathy, microalbuminuria or macroalbuminuria.

Patients were treated with either atorvastatin 10 mg daily (n=1,428) or placebo (n=1,410) for a median follow-up of 3.9 years.

The absolute and relative risk reduction effect of atorvastatin was as follows:

Event	*Relative risk reduction (%)*	*No. of events (atorvastatin vs placebo)*	*Absolute risk reduction[1] (%)*	*P value*
Major cardiovascular events (fatal and non-fatal AMI, silent MI, acute CHD death, unstable angina, CABG, PTCA, revascularisation, stroke)	37%	83 vs. 127	3.2%	0.0010
MI (fatal and non-fatal AMI, silent MI)	42%	38 vs. 64	1.9%	0.0070
Strokes (fatal and non-fatal)	48%	21 vs 39	1.3%	0.0163

[1] Based on difference in crude events rates occurring over a median follow-up of 3.9 years.
AMI = acute myocardial infarction; CABG = coronary artery bypass graft; CHD = coronary heart disease; MI = myocardial infarction; PTCA = percutaneous transluminal coronary angioplasty.

There was no evidence of a difference in the treatment effect by patient's gender, age, or baseline LDL-C level. A favourable trend was observed regarding the mortality rate (82 deaths in the placebo group vs. 61 deaths in the atorvastatin group, p=0.0592).

<u>Recurrent stroke</u>

In the Stroke Prevention by Aggressive Reduction in Cholesterol Levels (SPARCL) study, the effect of atorvastatin 80 mg daily or placebo on stroke was evaluated in 4,731 patients who had a stroke or transient ischaemic attack (TIA) within the preceding 6 months and no history of coronary heart disease (CHD). Patients were 60% male, 21-92 years of age (average age 63 years) and had an average baseline LDL of 133 mg/dL (3.4 mmol/L). The mean LDL-C was 73 mg/dL (1.9 mmol/L) during treatment with atorvastatin and 129 mg/dL (3.3 mmol/L) during treatment with placebo. Median follow-up was 4.9 years.

Atorvastatin 80 mg reduced the risk of the primary endpoint of fatal or non-fatal stroke by 15% (HR 0.85; 95% CI, 0.72-1.00; p=0.05 or 0.84; 95% CI, 0.71-0.99; p=0.03 after adjustment for baseline factors) compared to placebo. All-cause mortality was 9.1% (216/2,365) for atorvastatin versus 8.9% (211/2,366) for placebo.

In a *post-hoc* analysis, atorvastatin 80 mg reduced the incidence of ischaemic stroke (218/2,365, 9.2% vs. 274/2,366, 11.6%, p=0.01) and increased the incidence of haemorrhagic stroke (55/2,365, 2.3% vs. 33/2,366, 1.4%, p=0.02) compared to placebo.

- The risk of haemorrhagic stroke was increased in patients who entered the study with prior haemorrhagic stroke (7/45 for atorvastatin versus 2/48 for placebo; HR 4.06; 95% CI, 0.84-19.57) and the risk of ischaemic stroke was similar between groups (3/45 for atorvastatin versus 2/48 for placebo; HR 1.64; 95% CI, 0.27-9.82).

- The risk of haemorrhagic stroke was increased in patients who entered the study with prior lacunar infarct (20/708 for atorvastatin versus 4/701 for placebo; HR 4.99; 95% CI, 1.71-14.61), but the risk of ischaemic stroke was also decreased in these patients (79/708 for atorvastatin versus 102/701 for placebo; HR 0.76; 95% CI, 0.57-1.02). It is possible that the net risk of stroke is increased in patients with prior lacunar infarct who receive atorvastatin 80 mg/day.

All-cause mortality was 15.6% (7/45) for atorvastatin versus 10.4% (5/48) in the subgroup of patients with prior haemorrhagic stroke. All-cause mortality was 10.9% (77/708) for atorvastatin versus 9.1% (64/701) for placebo in the subgroup of patients with prior lacunar infarct.

Paediatric Population

Heterozygous Familial Hypercholesterolaemia in Paediatric Patients aged 6-17 years old

An 8-week, open-label study to evaluate pharmacokinetics, pharmacodynamics, and safety and tolerability of atorvastatin was conducted in children and adolescents with genetically confirmed heterozygous familial hypercholesterolemia and baseline LDL-C $\geq$ 4 mmol/L. A total of 39 children and adolescents, 6 to 17 years of age, were enrolled. Cohort A included 15 children, 6 to 12 years of age and at Tanner Stage 1. Cohort B included 24 children, 10 to 17 years of age and at Tanner Stage ≥ 2.

The initial dose of atorvastatin was 5 mg daily of a chewable tablet in Cohort A and 10 mg daily of a tablet formulation in Cohort B. The atorvastatin dose was permitted to be doubled if a subject had not attained target LDL-C of < 3.35 mmol/L at Week 4 and if atorvastatin was well tolerated.

Mean values for LDL-C, TC, VLDL-C, and Apo B decreased by Week 2 among all subjects. For subjects whose dose was doubled, additional decreases were observed as early as 2 weeks, at the first assessment, after dose escalation. The mean percent decreases in lipid parameters were similar for both cohorts, regardless of whether subjects remained at their initial dose or doubled their initial dose. At Week 8, on average, the percent change from baseline in LDL-C and TC was approximately 40% and 30%, respectively, over the range of exposures.

Heterozygous Familial Hypercholesterolaemia in Paediatric Patients aged 10-17 years old

In a double-blind, placebo controlled study followed by an open-label phase, 187 boys and postmenarchal girls 10-17 years of age (mean age 14.1 years) with heterozygous familial hypercholesterolaemia (FH) or severe hypercholesterolaemia were randomised to atorvastatin (n=140) or placebo (n=47) for 26 weeks and then all received atorvastatin for 26 weeks.. The dosage of atorvastatin (once daily) was 10 mg for the first 4 weeks and up-titrated to 20 mg if the LDL-C level was > 3.36 mmol/l. Atorvastatin significantly decreased plasma levels of total-C, LDL-C, triglycerides, and apolipoprotein B during the 26 week double-blind phase. The mean achieved LDL-C value was 3.38 mmol/l (range: 1.81-6.26 mmol/l) in the atorvastatin group compared to 5.91 mmol/l (range: 3.93-9.96 mmol/l) in the placebo group during the 26-week double-blind phase.

An additional paediatric study of atorvastatin versus colestipol in patients with hypercholesterolaemia aged 10-18 years demonstrated that atorvastatin (N=25) caused a significant reduction in LDL-C at week 26 (p<0.05) compared with colestipol (N=31).

A compassionate use study in patients with severe hypercholesterolaemia (including homozygous hypercholesterolaemia) included 46 paediatric patients treated with atorvastatin titrated according to response (some subjects received 80 mg atorvastatin per day). The study lasted 3 years: LDL-cholesterol was lowered by 36%.

The long-term efficacy of atorvastatin therapy in childhood to reduce morbidity and mortality in adulthood has not been established.

The European Medicines Agency has waived the obligation to submit the results of studies with atorvastatin in children aged 0 to less than 6 years in the treatment of heterozygous hypercholesterolaemia and in children aged 0 to less than 18 years in the treatment of homozygous familial hypercholesterolaemia, combined (mixed) hypercholesterolaemia, primary hypercholesterolaemia and in the prevention of cardiovascular events (see section 4.2 for information on paediatric use).

5.2 PHARMACOKINETIC PROPERTIES

Absorption

Atorvastatin is rapidly absorbed after oral administration; maximum plasma concentrations (C_{max}) occur within 1 to 2 hours. Extent of absorption increases in proportion to atorvastatin dose. After oral administration, atorvastatin film-coated tablets are 95% to 99% bioavailable compared to the oral solution. The absolute bioavailability of atorvastatin is approximately 12% and the systemic availability of HMG-CoA reductase inhibitory activity is approximately 30%. The low systemic availability is attributed to presystemic clearance in gastrointestinal mucosa and/or hepatic first-pass metabolism

Distribution

Mean volume of distribution of atorvastatin is approximately 381 L. Atorvastatin is

≥98% bound to plasma proteins.

Metabolism

Atorvastatin is metabolised by cytochrome P450 3A4 to ortho- and parahydroxylated derivatives and various beta-oxidation products. Apart from other pathways these products are further metabolised via glucuronidation. *In vitro*, inhibition of HMGCoA reductase by ortho- and parahydroxylated metabolites is equivalent to that of atorvastatin. Approximately 70% of circulating inhibitory activity for HMG-CoA reductase is attributed to active metabolites.

Excretion

Atorvastatin is eliminated primarily in bile following hepatic and/or extrahepatic metabolism. However, atorvastatin does not appear to undergo significant enterohepatic recirculation. Mean plasma elimination half-life of atorvastatin in humans is approximately 14 hours. The half-life of inhibitory activity for HMG-CoA reductase is approximately 20 to 30 hours due to the contribution of active metabolites.

Special populations

- Elderly: Plasma concentrations of atorvastatin and its active metabolites are higher in healthy elderly subjects than in young adults while the lipid effects were comparable to those seen in younger patient populations.
- Paediatric: In an open-label, 8-week study, Tanner Stage 1 (N=15) and Tanner Stage ≥ 2 (N=24) paediatric patients (ages 6-17 years) with heterozygous familial hypercholesterolemia and baseline LDL-C ≥ 4 mmol/L were treated with 5 or 10 mg of chewable or 10 or 20 mg of film-coated atorvastatin tablets once daily, respectively. Body weight was the only significant covariate in atorvastatin population PK model. Apparent oral clearance of atorvastatin in paediatric subjects appeared similar to adults when scaled allometrically by body weight. Consistent decreases in LDL-C and TC were observed over the range of atorvastatin and o-hydroxyatorvastatin exposures.
- Gender: Concentrations of atorvastatin and its active metabolites in women differ from those in men (women: approximately 20% higher for C_{max} and approximately 10% lower for AUC). These differences were of no clinical significance, resulting in no clinically significant differences in lipid effects among men and women.
- Renal insufficiency: Renal disease has no influence on the plasma concentrations or lipid effects of atorvastatin and its active metabolites.
- Hepatic insufficiency: Plasma concentrations of atorvastatin and its active metabolites are markedly increased (approximately 16-fold in C_{max} and approximately 11-fold in AUC) in patients with chronic alcoholic liver disease (Childs-Pugh B).
- SLOC1B1 polymorphism: Hepatic uptake of all HMG-CoA reductase inhibitors including atorvastatin, involves the OATP1B1 transporter. In patients with SLCO1B1 polymorphism there is a risk of increased exposure of atorvastatin, which may lead to an increased risk of rhabdomyolysis (see section 4.4). Polymorphism in the gene encoding OATP1B1 (SLCO1B1 c.521CC) is associated with a 2.4-fold higher atorvastatin exposure (AUC) than in individuals without this genotype variant (c.521TT). A genetically impaired hepatic uptake of atorvastatin is also possible in these patients. Possible consequences for the efficacy are unknown.

5.3 PRECLINICAL SAFETY DATA

Atorvastatin was negative for mutagenic and clastogenic potential in a battery of 4 in vitro tests and 1 in vivo assay. Atorvastatin was not found to be carcinogenic in rats, but high doses in mice (resulting in 6-11 fold the AUC0-24h reached in humans at the highest recommended dose) showed hepatocellular adenomas in males and hepatocellular carcinomas in females.

There is evidence from animal experimental studies that HMG-CoA reductase inhibitors may affect the development of embryos or fetuses. In rats, rabbits and dogs atorvastatin had no effect on fertility and was not teratogenic, however, at maternally toxic doses fetal toxicity was observed in rats and rabbits. The development of the rat offspring was delayed and post-natal survival reduced during exposure of the dams to high doses of atorvastatin. In rats, there is evidence of placental transfer. In rats, plasma concentrations of atorvastatin are similar to those in milk. It is not known whether atorvastatin or its metabolites are excreted in human milk.

6 PHARMACEUTICAL PARTICULARS

6.1 LIST OF EXCIPIENTS

Tablet core

Microcrystalline cellulose

Sodium carbonate anhydrous Maltose

Croscarmellose sodium

Magnesium stearate

Film-coating

Hypromellose (E464)

Hydroxypropylcellulose

Triethyl citrate (E1505)

Polysorbate 80

Titanium dioxide (E171).

6.2 INCOMPATIBILITIES

Not applicable

6.3 SHELF LIFE

2 years

6.4 SPECIAL PRECAUTIONS FOR STORAGE

Store below 30°C

6.5 NATURE AND CONTENTS OF CONTAINER

Aluminium-aluminium blisters.

Atorvastatin 20 mg Film-coated Tablets are available in pack sizes of 7, 10, 14, 15, 20, 28, 30, 50, 50x1, 56, 60, 84, 90, 98, 100 or 200 tablets.

High density polyethylene bottle with a polypropylene cap provided with a compartment for desiccant.

Atorvastatin 20 mg Film-coated Tablets is available in pack sizes of 50, 100 tablets and as multipack containing 100 tablets (2 bottles of 50 tablets)

Not all pack sizes may be marketed.

6.6 SPECIAL PRECAUTIONS FOR DISPOSAL

No special requirements.

Any unused product or waste should be disposed of in accordance with local requirements.

7 MARKETING AUTHORISATION HOLDER

TEVA UK Limited
Brampton Road,
Hampden Park,
Eastbourne,
East Sussex BN22 9AG
UNITED KINGDOM

8 MARKETING AUTHORISATION NUMBER(S)

PL 00289/1290

9 DATE OF FIRST AUTHORISATION/RENEWAL OF THE AUTHORISATION

03/08/2010

10 DATE OF REVISION OF THE TEXT

02/09/2015

StatinWISE Patient Recruitment Pathway, FINAL Version 1.0, 23 May 2017

<u>Appendix 9:</u> StatinWISE Patient Recruitment Pathway

IRAS: 197990

Diagram illustrating communication pathway following patient initiated contact

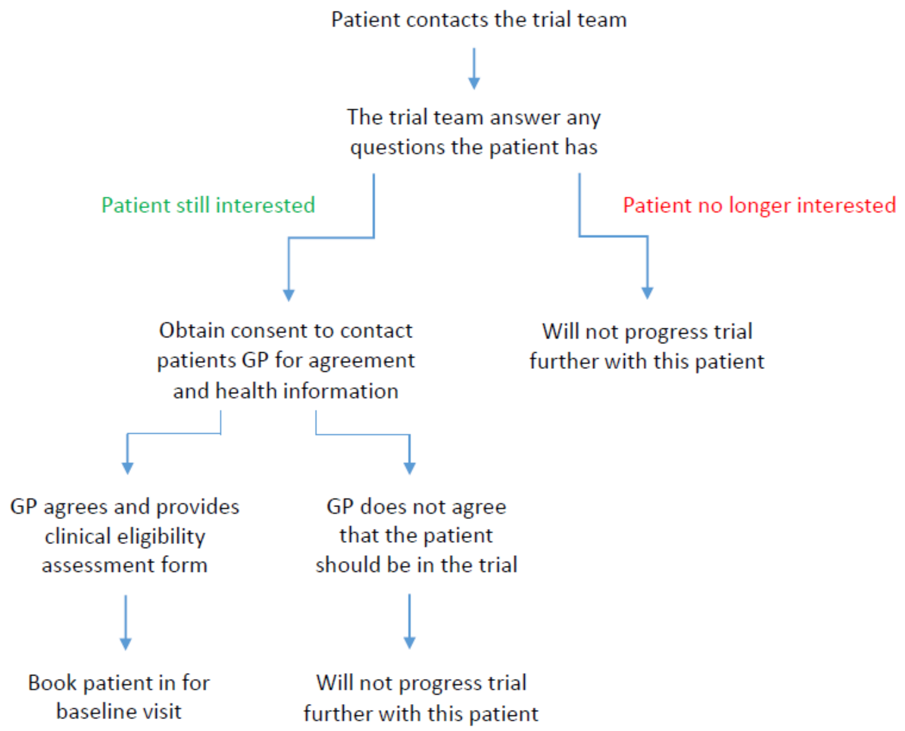

Recruitment Pathway_FINAL Version 1.0, 23 May 2017

NHS
National Institute for
Health Research
