# Supplementary material for: Statin treatment and muscle symptoms: series of randomised, placebo controlled n-of-1 trials
Source: BMJ. 2021 Feb 24;372:n135. doi: 10.1136/bmj.n135 (PMC7903384; doi:10.1136/bmj.n135)
Supplement: Supplementary file 4 — Web appendix 4: Personalised results document [file here059829.ww4.pdf]

# STATINWISE

# PATIENT XXXX PERSONALISED RESULTS

statinwise.lshtm.ac.uk


Statin WISE

LONDON SCHOOL of HYGIENE & TROPICAL MEDICINE

CLINICAL TRIALS UNIT

statinwise.lshtm.ac.uk

# WELCOME TO YOUR PERSONALISED RESULTS

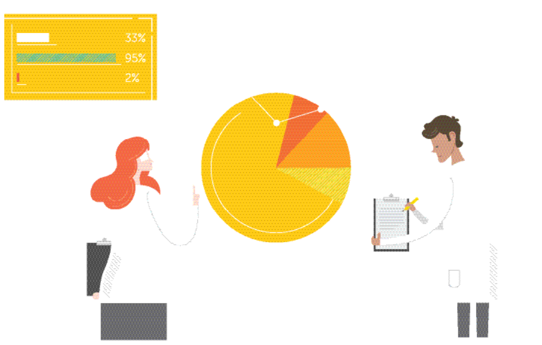

**Dear patient XXXX,**

Thank you for taking part in the StatinWISE trial. We hope that you found taking part informative and worthwhile. Your contribution is greatly appreciated.

This document contains your personalised results. The primary goal of this document is to increase both your and our understanding of statin related muscle side effects and to help you decide whether you want to carry on taking a statin long-term.

To achieve this goal, we asked you to report muscle symptoms at the end of each treatment period. Each treatment period, lasting 8 weeks, was either a statin or a placebo. We also asked how your muscle symptoms affected your daily life. You did this over the course of approximately 12 months which included 6 out of a possible 6 treatment periods.

We will show your data in a series of graphs in the sequence that you took statin and placebo. We will indicate how often you took your study medication because this may affect how to interpret this data.

**This document contains your data in two parts**

1. How statin and placebo affected your muscle symptoms
2. How your muscle symptoms affected your daily life

The final part of the document will describe what this means to you.

**What to do next**

Please read this document thoroughly. A copy of your personalised results was sent to Dr XXXX XXXXX and the research nurse XXXX XXXXX at XXXX XXXX who you may have met when you joined the study.

**For patients completing 6 treatment periods**

Your research nurse will call you to arrange an appointment to go through this document. This appointment may be face-to-face or over the telephone and will be with Dr XXXXX or XXXX XXXXX. Within 4 weeks of this appointment, XXXX will call you to discuss if you have decided to continue to take statins or not and ask if this document helped you to make that decision. This will conclude your participation in StatinWISE.

We will finish collecting data from all 200 patients in the summer of 2019. At that point, we will publish the anonymised data in a medical journal. At that point, we will send you a summary of the overall results.

**Yours Sincerely**

**StatinWISE Team**

# CONTENTS

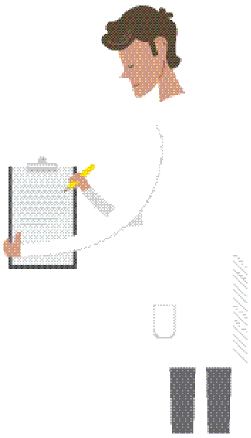


| Page | SECTION |
|---|---|
| **1** | **INFORMATION ABOUT YOUR PARTICIPATION** |
| 1 | KEY DATES |
| 1 | YOUR TREATMENT SEQUENCE |
| **2** | **YOUR RESULTS** |
| 2 | MUSCLE SYMPTOMS |
| **3** | YOUR DAILY MUSCLE SYMPTOM SCORES WHEN TAKING STATIN AND PLACEBO |
| **4** | HOW TAKING STATIN AFFECTED YOUR QUALITY OF LIFE |
| 6 | GENERAL ACTIVITY |
| 7 | MOOD |
| 8 | WALKING ABILITY |
| 9 | NORMAL WORK |
| 10 | RELATIONS WITH OTHER PEOPLE |
| 11 | SLEEP |
| 12 | ENJOYMENT OF LIFE |
| **13** | **WHAT DOES THIS MEAN TO YOU?** |

# INFORMATION ABOUT YOUR PARTICIPATION

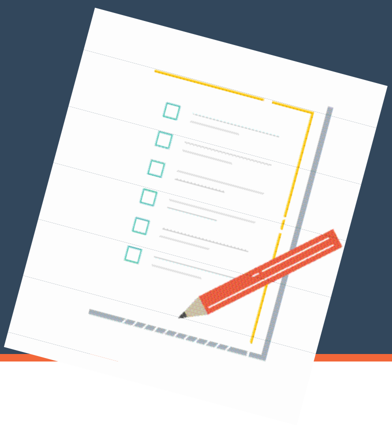

## KEY DATES

| | |
|---|---|
| You joined the study on: | DD MMM YYYY |
| You started taking study medication on: | DD MMM YYYY |
| You finished taking study medication on: | DD MMM YYYY |
| This was treatment period: | 6 |

You were the XXth patient to be recruited, out of a target of 200 patients.

## YOUR TREATMENT SEQUENCE WAS:

| Placebo | Statin | Statin | Placebo | Placebo | Statin |
|---|---|---|---|---|---|
| DD MMM YYYY – DD MMM YYYY | DD MMM YYYY – DD MMM YYYY | DD MMM YYYY – DD MMM YYYY | DD MMM YYYY – DD MMM YYYY | DD MMM YYYY – DD MMM YYYY | DD MMM YYYY – DD MMM YYYY |
| 1 | 2 | 3 | 4 | 5 | 6 |

Treatment period

**Reason for finishing:**

You successfully completed the trial. Congratulations and well done!

# YOUR RESULTS

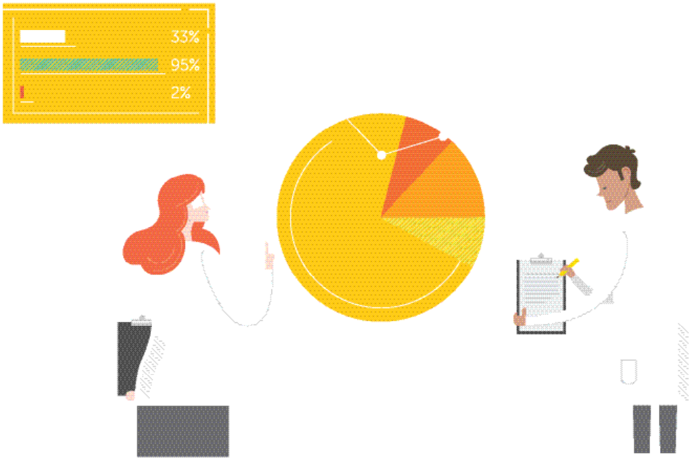


## MUSCLE SYMPTOMS

During your participation you provided muscle symptom scores every day for seven days at the end of each treatment period. The data below shows your average muscle symptom scores for when you took statin and when you took placebo.

On a scale of 0 (no symptoms) to 10 (worst possible symptoms) your average symptom score was:

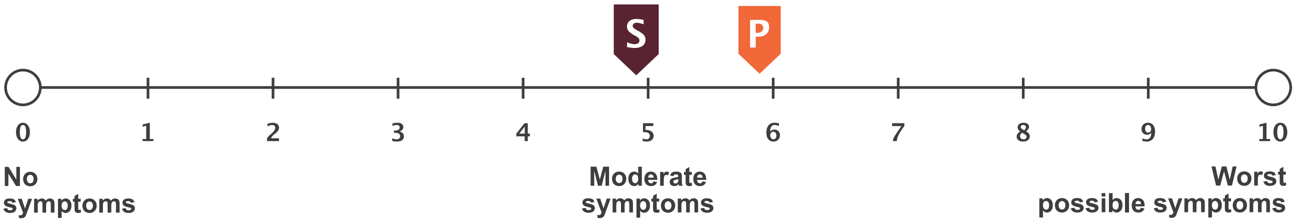


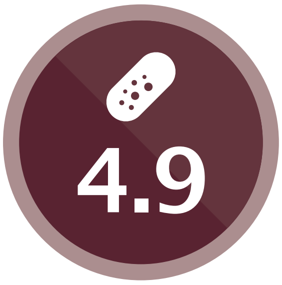


Average muscle symptom score during statin (S) treatment

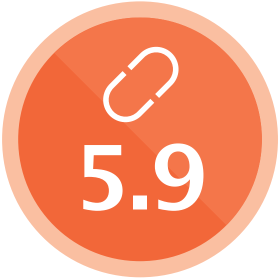


Average muscle symptom score during placebo (P) treatment

**How to interpret your average muscle symptom scores**

Your average muscle symptom scores during statin and placebo periods will help you understand if the symptoms you experienced were due to the statin or not. A difference in average scores of 1 point or more, suggests that the treatment influences this symptom.

**For example:** An average symptom score whilst taking a statin for patient X was 7. An average symptom score while taking a placebo for patient X was 5. The symptom score whilst taking a statin was higher than whilst taking placebo with a difference in symptom score of 2 points. This suggests that the statin may be making patient X's muscle symptoms worse.

Your average score was lower during statin periods with a difference in your average scores during statin and placebo periods of 1.0.

This suggests that 20 mg Atorvastatin is not contributing to your muscle symptoms.

# DAILY MUSCLE SYMPTOM SCORES WHEN TAKING STATIN AND PLACEBO

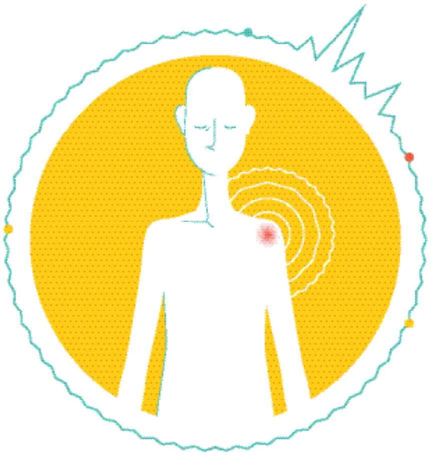

This more detailed graph displays each of your daily syr [illegible] participated in StatinWISE. Each symbol corresponds to each day that you provided pain data. The grey capsules above the graph represent how frequently you took the study medication. Please refer to the "Key" at the bottom of the page for a description.

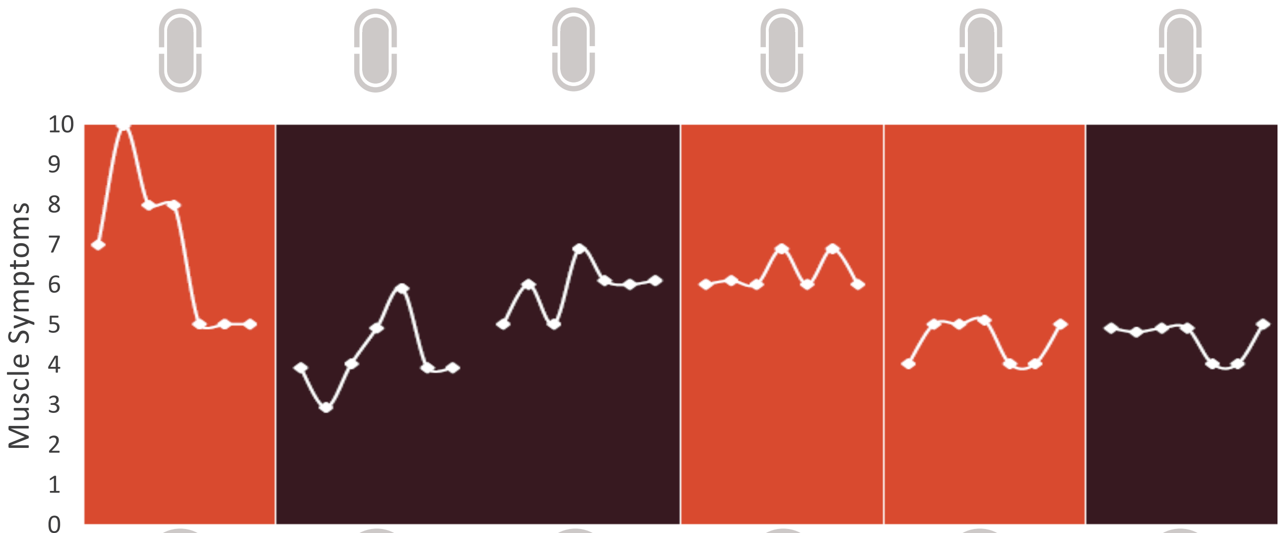


Key:

**100% capsule** Took medication every day

**75% capsule** Took medication most days, missing the occasional dose

**50% capsule** Took medication some days

**25% capsule** Took medication few days

**0% capsule** Didn't take medication

Placebo

Statin

# HOW TAKING STATIN AFFECTED YOUR DAILY LIFE

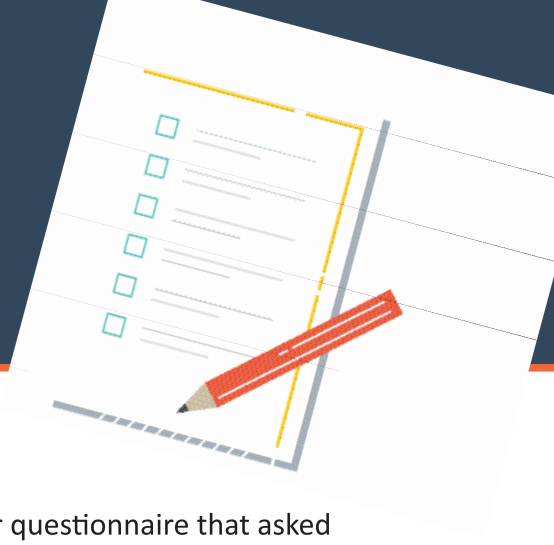

On day 56 of each treatment period, you completed a slightly longer questionnaire that asked about symptoms over the last two months. All of the information from this point reflects the data collected from those questionnaires.

| Did you experience muscle symptoms (e.g. muscle pain or weakness)? | | Your response |
|---|---|---|
| 1 | Treatment period 1 – Placebo | Yes |
| 2 | Treatment period 2 – Statin | Yes |
| 3 | Treatment period 3 – Statin | Yes |
| 4 | Treatment period 4 – Placebo | Yes |
| 5 | Treatment period 5 – Placebo | Yes |
| 6 | Treatment period 6 – Statin | Yes |

| Did you think these symptoms were related to the study medicaton? | | Your response |
|---|---|---|
| 1 | Treatment period 1 – Placebo | Don't know |
| 2 | Treatment period 2 – Statin | Don't know |
| 3 | Treatment period 3 – Statin | Don't know |
| 4 | Treatment period 4 – Placebo | Don't know |
| 5 | Treatment period 5 – Placebo | Don't know |
| 6 | Treatment period 6 – Statin | Don't know |

# HOW TAKING STATIN AFFECTED YOUR DAILY LIFE

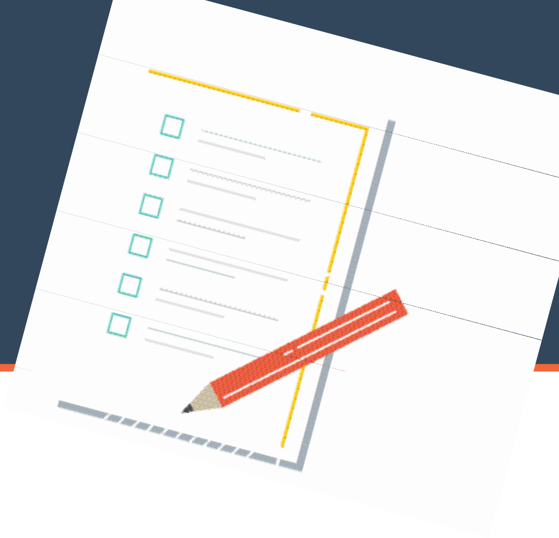

### Where you experienced your muscle symptoms

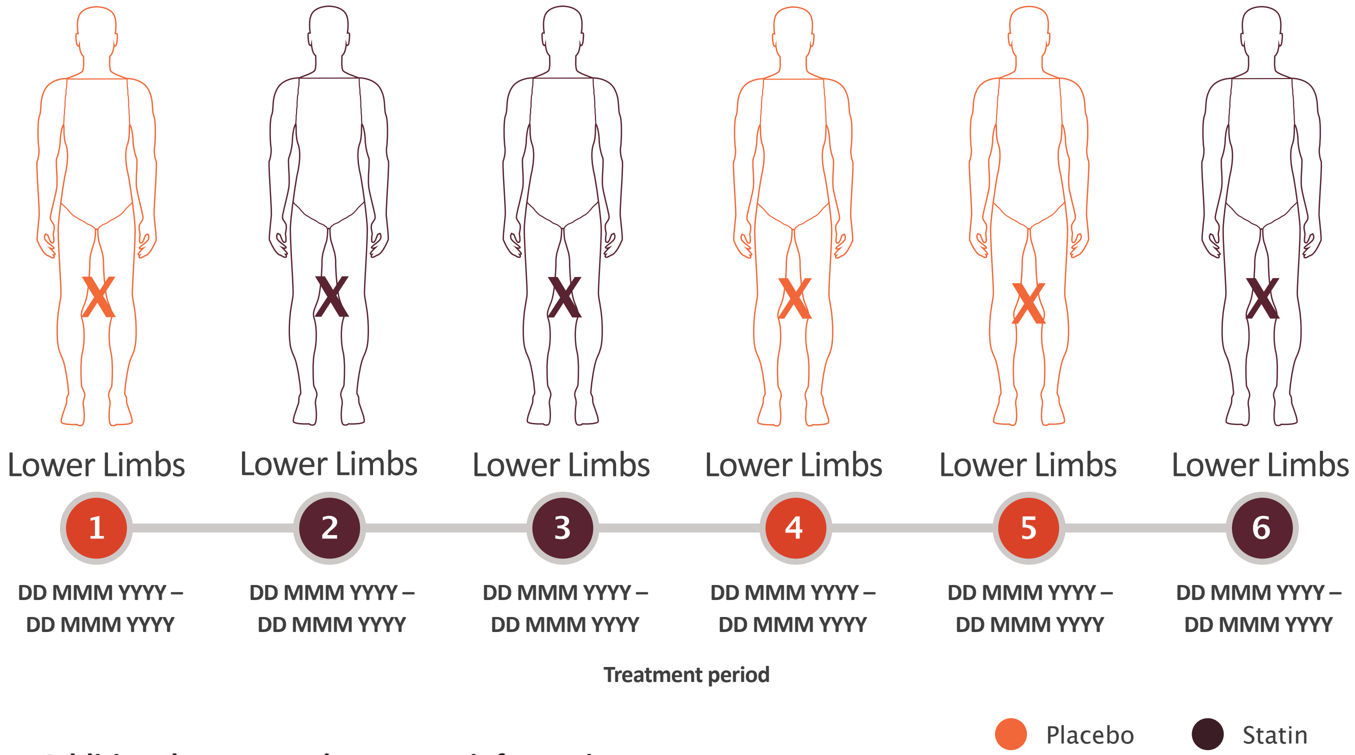


**Additional events and symptom information to note:**

Treatment period 1 - Placebo:

i) You had muscle symptoms in more than one area.

ii) You were still having muscle symptoms despite stopping statins 2 years ago.

Treatment period 2 - Statin:

i) You had muscle symptoms in more than one area.

ii) You think the muscle symptoms were possibly caused by the flight when you went on holiday

Treatment period 3 - Statin:

i) You had muscle symptoms in more than one area.

ii) You also had muscle symptoms in upper arms, shoulder and sometimes neck.

Treatment period 4 - Placebo:

i) You had muscle symptoms in more than one area.

Treatment period 5 - Placebo:

i) You had muscle symptoms in more than one area.

ii)Your symptoms are not causing you as much bother now you have moved to a bungalow, so it is easier to get into the garden.

Treatment period 6 - Statin:

i) You had muscle symptoms in more than one area.

# HOW TAKING STATIN AFFECTED YOUR QUALITY OF LIFE

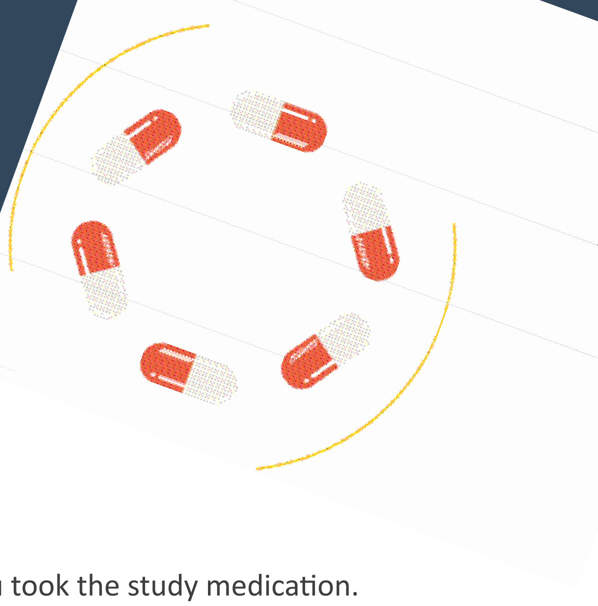

## GENERAL ACTIVITY

The grey capsules above the graph represent how frequently you took the study medication. Please refer to the “Key” at the bottom of the page for a description.

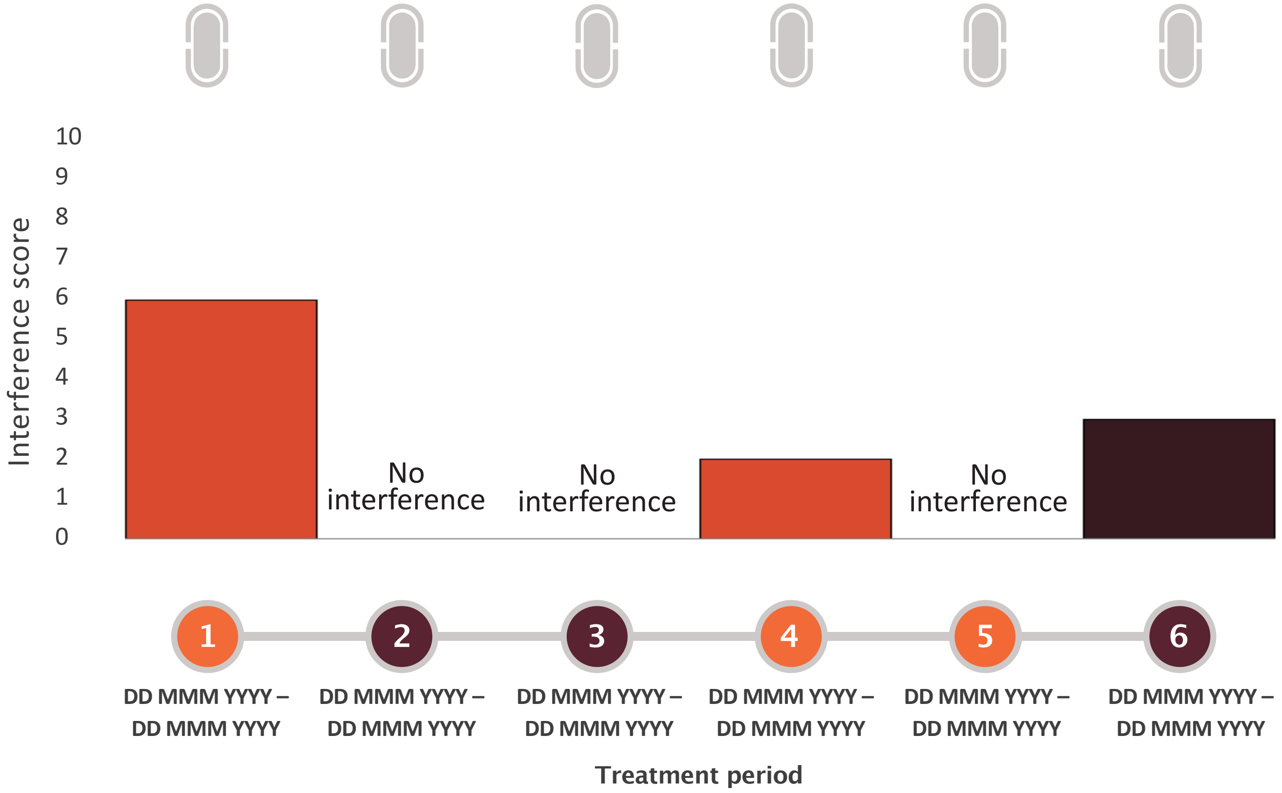


**1.0** — **Average interference score during statin treatment**

**2.7** — **Average interference score during placebo treatment**

### Comments

An average score that is at least 1 point greater during statin treatment compared to placebo treatment would suggest that the statin interfered with your general activity.

The difference in your average scores during statin and placebo periods was 1.7, with scores being higher during periods of placebo treatment.

This suggests that your muscle symptoms during statin periods are not interfering with your general activity more than placebo.

### Key

- **100% capsule** Took medication every day
- **75% capsule** Took medication most days, missing the occasional dose
- **50% capsule** Took medication some days
- **25% capsule** Took medication few days
- **0% capsule** Didn’t take medication
- Placebo
- Statin

# HOW TAKING STATIN AFFECTED YOUR QUALITY OF LIFE

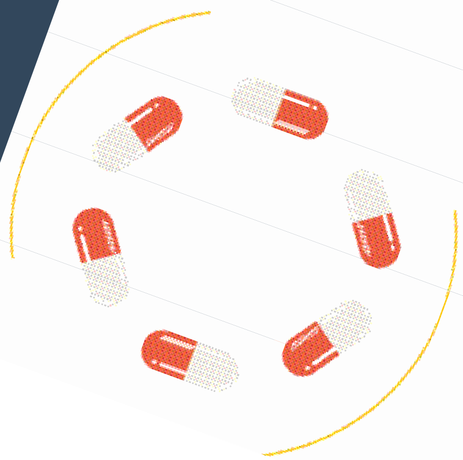

## MOOD

The grey capsules above the graph represent how frequently you took the study medication. Please refer to the "Key" at the bottom of the page for a description.

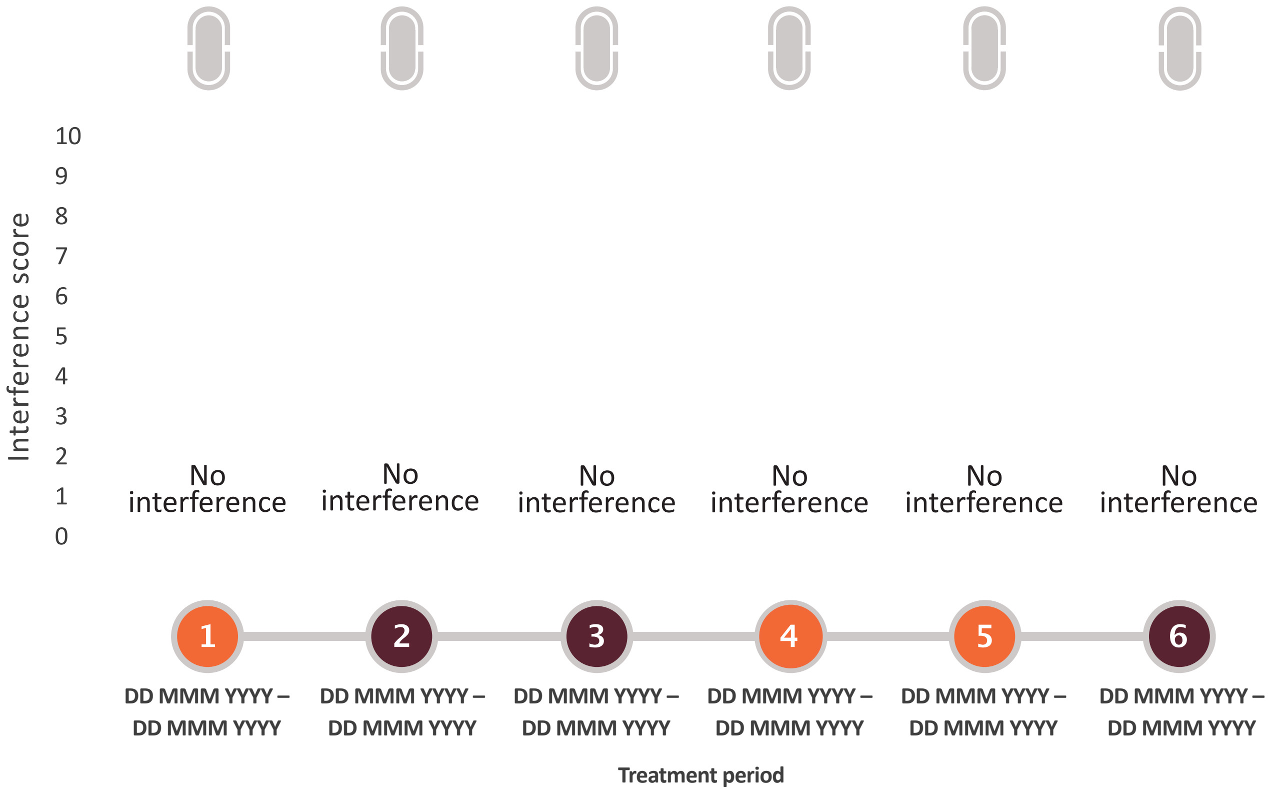


**0.0** **Average interference score during statin treatment**

**0.0** **Average interference score during placebo treatment**

### Comments

An average score that is at least 1 point greater during statin treatment compared to placebo treatment would suggest that the statin interfered with your mood.

The difference in your average scores during statin and placebo periods was zero.

This suggests that your muscle symptoms during statin periods are not interfering with your mood more than placebo.

### Key

**100% capsule**
Took medication every day

**75% capsule**
Took medication most days, missing the occasional dose

**50% capsule**
Took medication some days

**25% capsule**
Took medication few days

**0% capsule**
Didn't take medication

Placebo

Statin

# HOW TAKING STATIN AFFECTED YOUR QUALITY OF LIFE

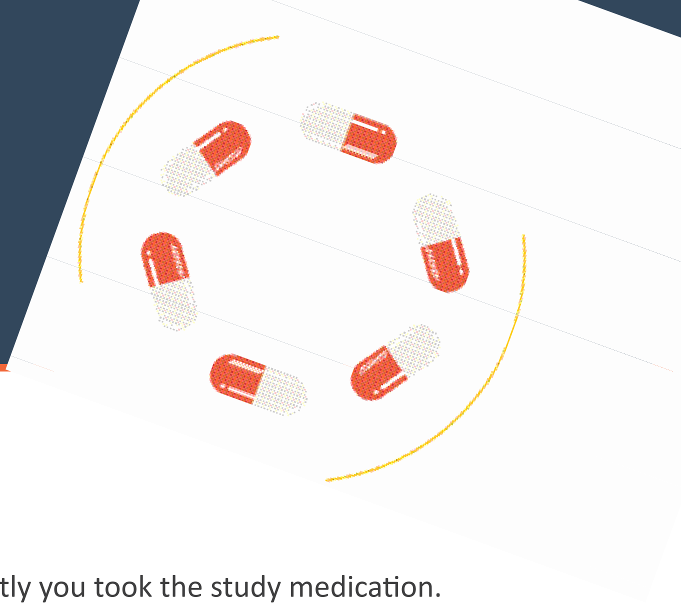

## WALKING ABILITY

The grey capsules above the graph represent how frequently you took the study medication. Please refer to the “Key” at the bottom of the page for a description.

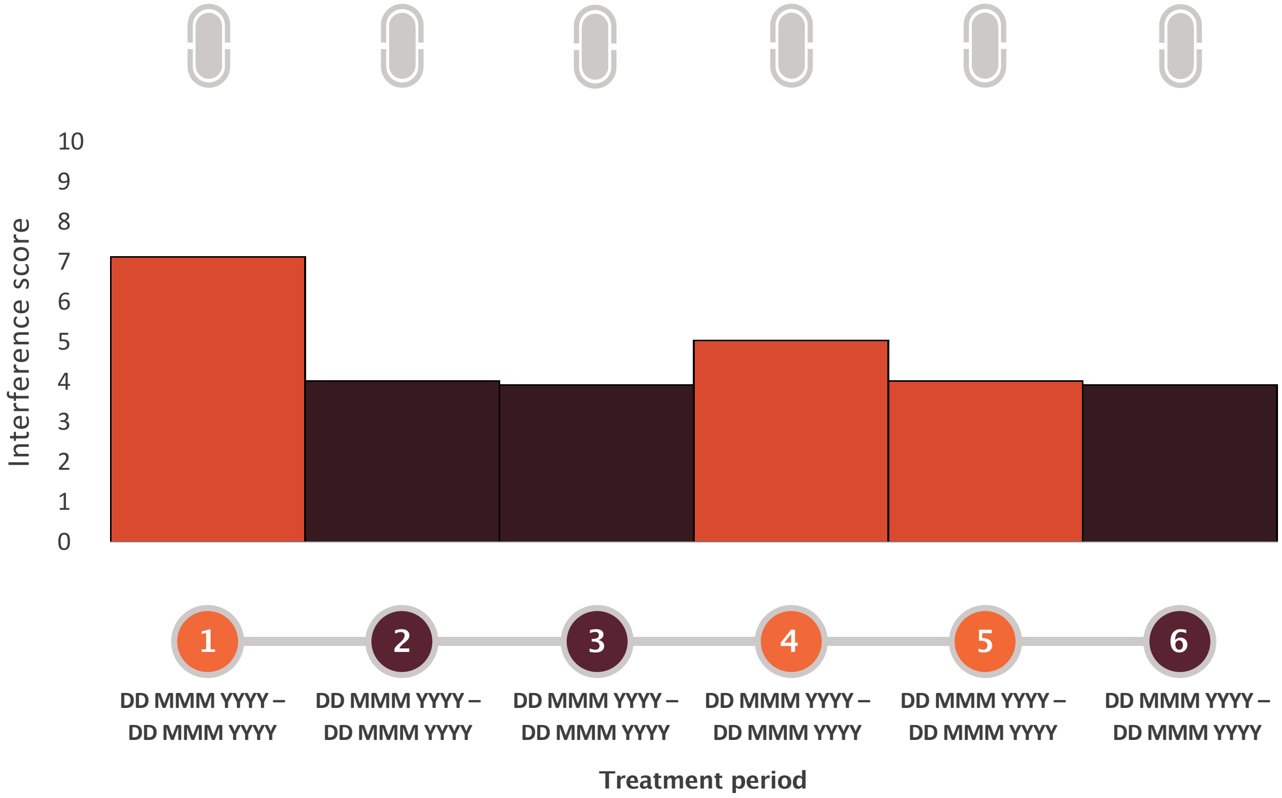


**3.9** Average interference score during statin treatment

**5.4** Average interference score during placebo treatment

**Comments**

An average score that is at least 1 point greater during statin treatment compared to placebo treatment would suggest that the statin interfered with your walking ability.

The difference in your average scores during statin and placebo periods was 1.5, with scores being higher during periods of placebo treatment.

This suggests that your muscle symptoms during statin periods are not interfering with your walking ability more than placebo.

**Key**

**100% capsule** Took medication every day

**75% capsule** Took medication most days, missing the occasional dose

**50% capsule** Took medication some days

**25% capsule** Took medication few days

**0% capsule** Didn’t take medication

Placebo

Statin

# HOW TAKING STATIN AFFECTED YOUR QUALITY OF LIFE

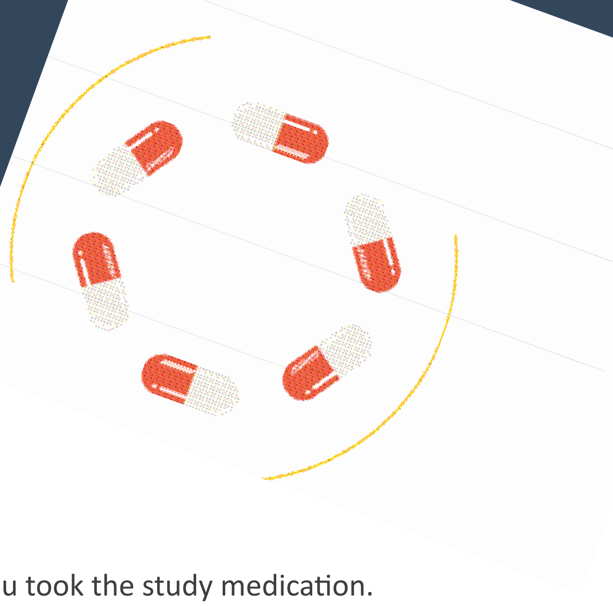

## NORMAL WORK

The grey capsules above the graph represent how frequently you took the study medication. Please refer to the “Key” at the bottom of the page for a description.

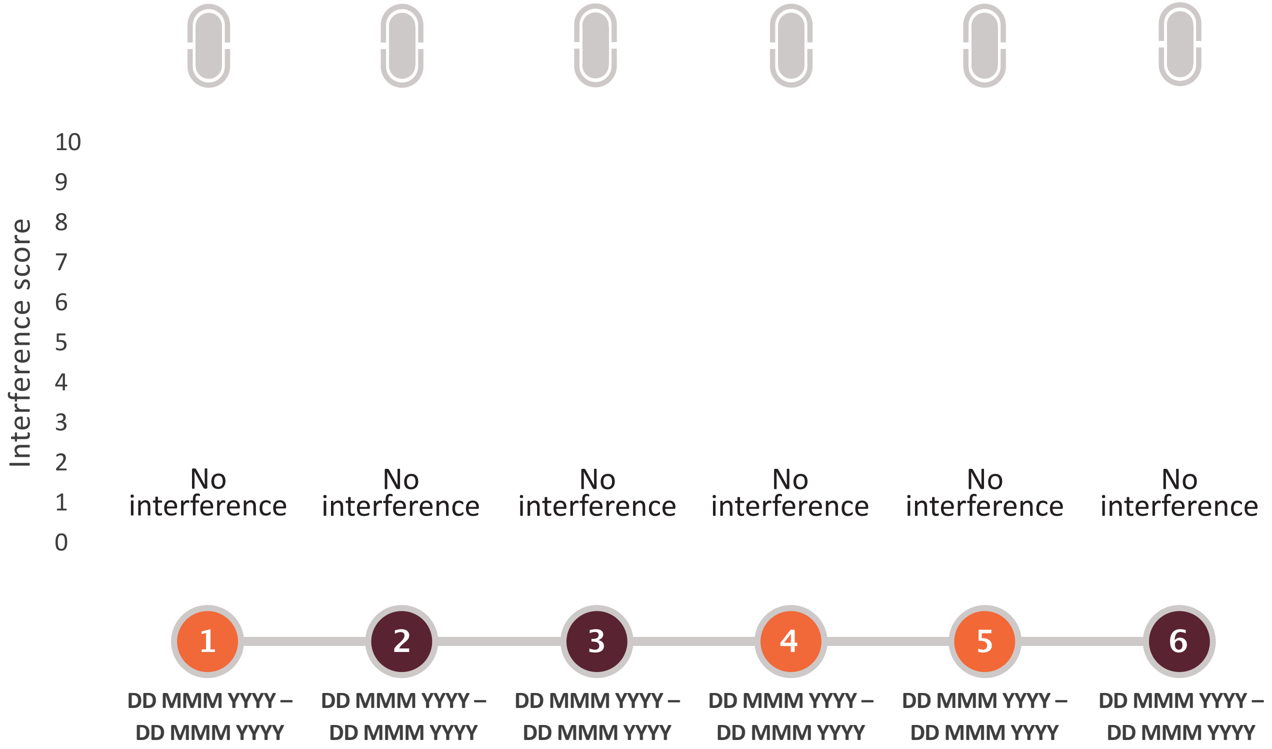


**0.0** **Average interference score during statin treatment**

**0.0** **Average interference score during placebo treatment**

**Comments**

An average score that is at least 1 point greater during statin treatment compared to placebo treatment would suggest that the statin interfered with your normal work.

The difference in your average scores during statin and placebo periods was zero.

This suggests that your muscle symptoms during statin periods are not interfering with your normal work more than placebo.

**Key**

**100% capsule** Took medication every day

**75% capsule** Took medication most days, missing the occasional dose

**50% capsule** Took medication some days

**25% capsule** Took medication few days

**0% capsule** Didn’t take medication

Placebo  Statin

# HOW TAKING STATIN AFFECTED YOUR QUALITY OF LIFE

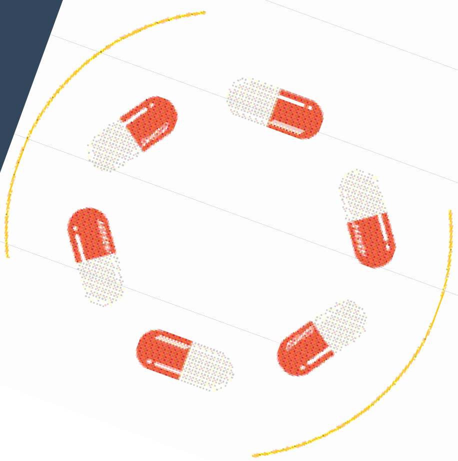

## RELATIONS WITH OTHERS

The grey capsules above the graph represent how frequently you took the study medication. Please refer to the “Key” at the bottom of the page for a description.

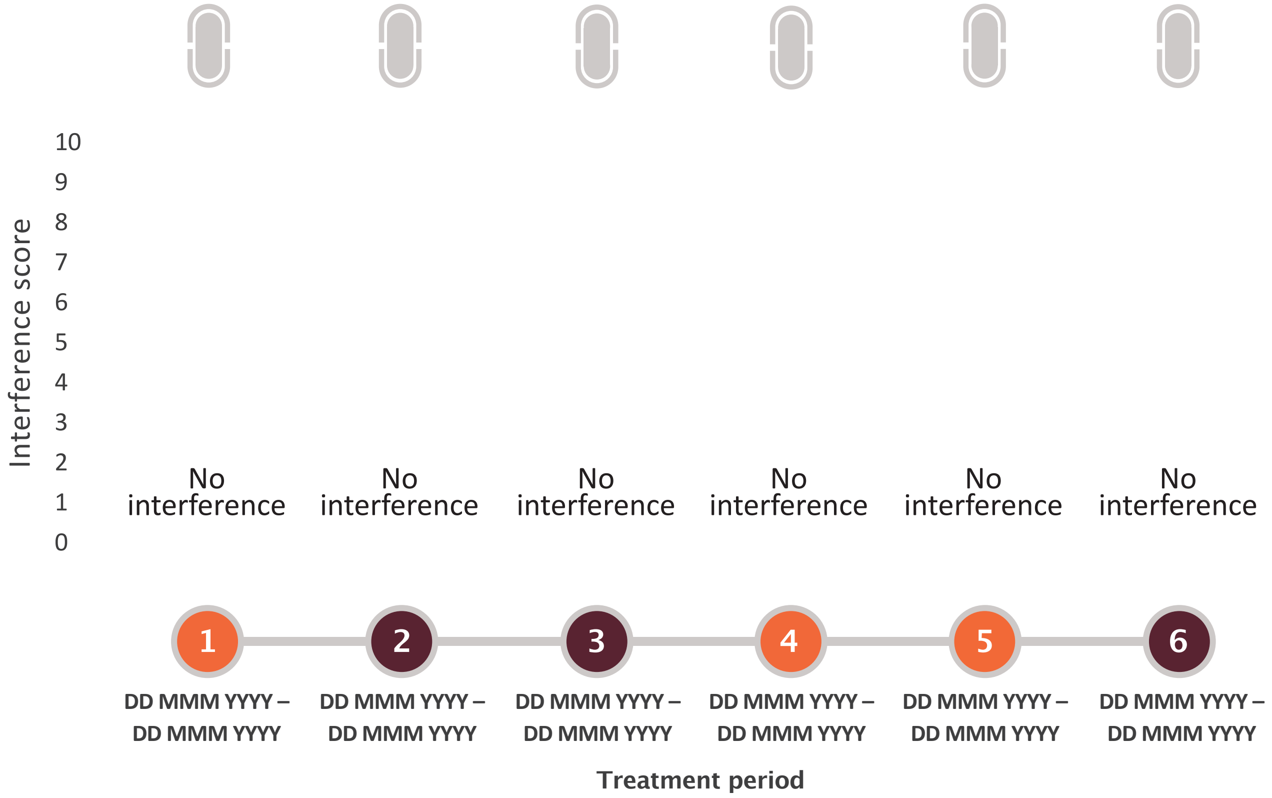


0.0 **Average interference score during statin treatment**

0.0 **Average interference score during placebo treatment**

**Comments**

An average score that is at least 1 point greater during statin treatment compared to placebo treatment would suggest that the statin interfered with your relations with other people.

The difference in your average scores during statin and placebo periods was zero.

This suggests that your muscle symptoms during statin periods are not interfering with your relations with other people more than placebo.

**Key**

- **100% capsule** Took medication every day
- **75% capsule** Took medication most days, missing the occasional dose
- **50% capsule** Took medication some days
- **25% capsule** Took medication few days
- **0% capsule** Didn’t take medication
- Placebo
- Statin

# HOW TAKING STATIN AFFECTED YOUR QUALITY OF LIFE

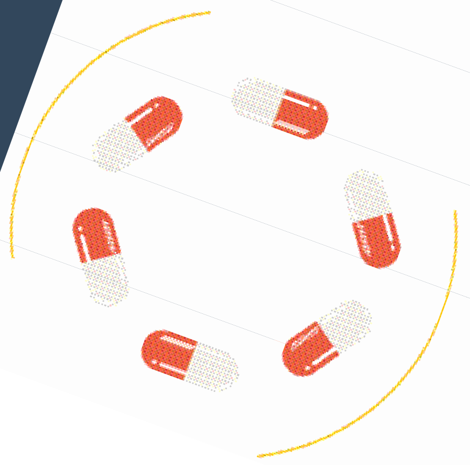

## SLEEP

The grey capsules above the graph represent how frequently you took the study medication. Please refer to the “Key” at the bottom of the page for a description.

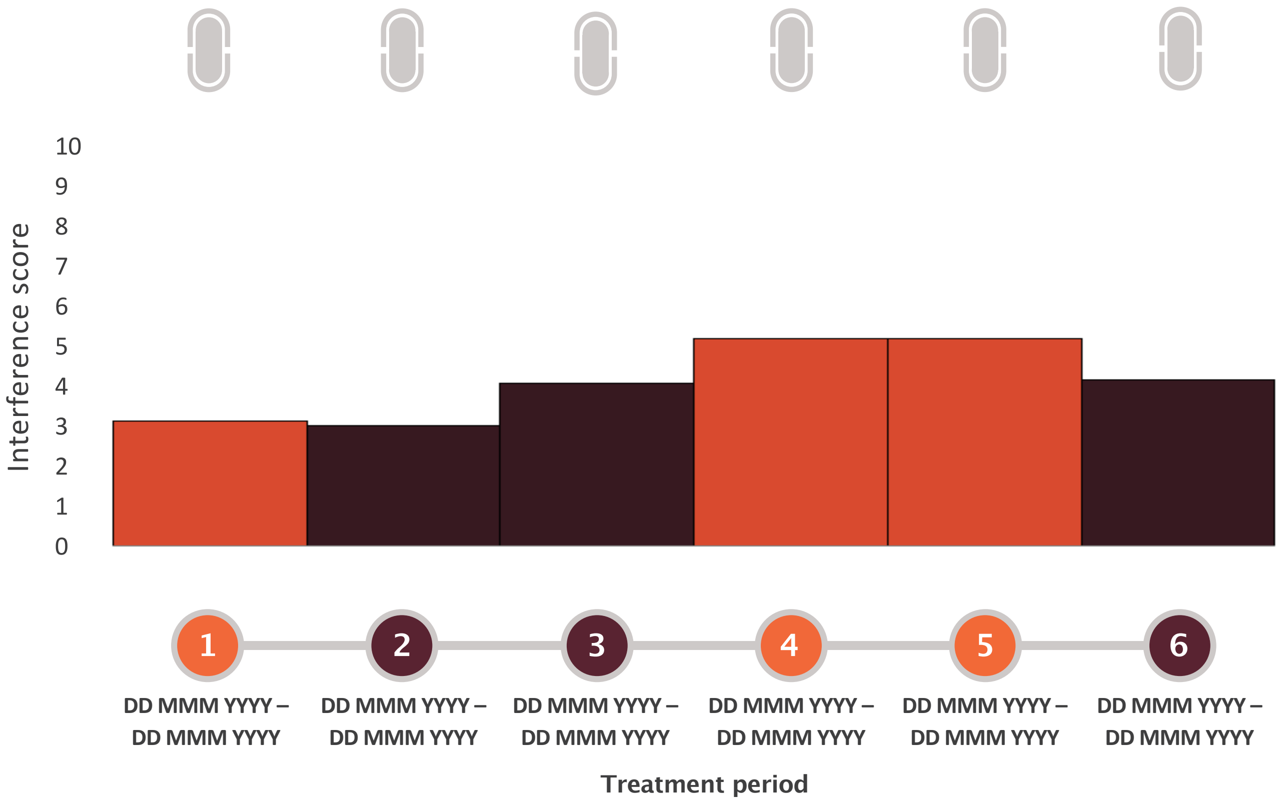


**3.6** Average interference score during statin treatment

**4.3** Average interference score during placebo treatment

### Comments

An average score that is at least 1 point greater during statin treatment compared to placebo treatment would suggest that the statin interfered with your sleep.

The difference in your average scores during statin and placebo periods was 0.7, with scores being slightly higher during periods of placebo treatment.

This suggests that your muscle symptoms during statin periods are not interfering with your sleep more than placebo.

### Key

**100% capsule** Took medication every day

**75% capsule** Took medication most days, missing the occasional dose

**50% capsule** Took medication some days

**25% capsule** Took medication few days

**0% capsule** Didn’t take medication

Placebo

Statin

# HOW TAKING STATIN AFFECTED YOUR QUALITY OF LIFE

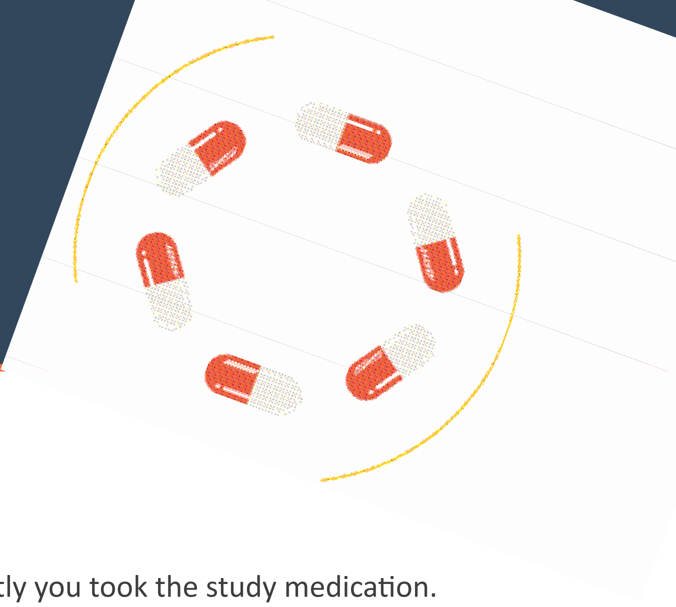

## ENJOYMENT OF LIFE

The grey capsules above the graph represent how frequently you took the study medication. Please refer to the "Key" at the bottom of the page for a description.

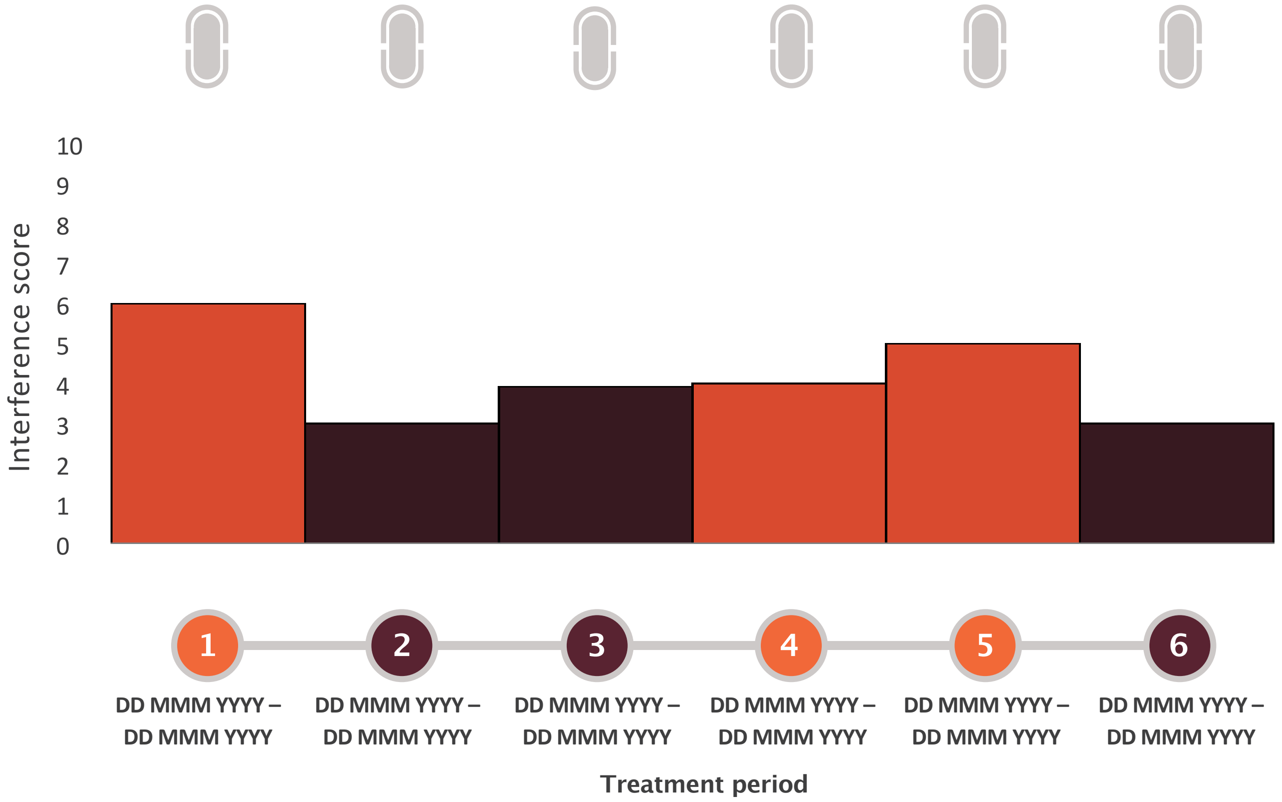


**3.3** **Average interference score during statin treatment**

**5.0** **Average interference score during placebo treatment**

### Comments

An average score that is at least 1 point greater during statin treatment compared to placebo treatment would suggest that the statin interfered with your enjoyment of life.

The difference in your average scores during statin and placebo periods was 1.7, with scores being higher during periods of placebo treatment.

This suggests that your muscle symptoms during statin periods are not interfering with your enjoyment of life more than placebo.

### Key

- **100% capsule** Took medication every day
- **75% capsule** Took medication most days, missing the occasional dose
- **50% capsule** Took medication some days
- **25% capsule** Took medication few days
- **0% capsule** Didn't take medication
- Placebo
- Statin

# WHAT THIS MEANS FOR YOU

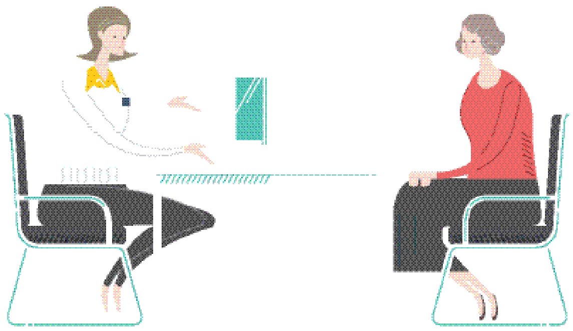

## WHAT THIS MEANS FOR YOU

This document was prepared to help you understand how your symptoms changed whilst you were taking statin and placebo. The purpose of this is to help you decide if you would like to continue long-term statin treatment now your participation in StatinWISE is over.

**Concerning your muscle symptoms**

Your results suggests that 20 mg Atorvastatin is not contributing to your muscle symptoms.

**Concerning your daily life**

Your results suggest that your muscle symptoms experienced on 20 mg Atorvastatin may not be interfering with your quality of life more than placebo.

| CRITERIA | INFLUENCED BY STATIN |
|---|---|
| Muscle symptoms | No |
| General activity | No |
| Mood | No |
| Walking ability | No |
| Normal work | No |
| Relations with other people | No |
| Sleep | No |
| Enjoyment of life | No |

Table - Effect of statin on your muscle symptoms and daily life

This concludes your personalised results. We hope that you find this useful.

We would like to take this opportunity to thank you once again for taking part. If you would like to provide positive or negative feedback on this document that may help improve it for other participants, please email statinwise@lshtm.ac.uk or call 0800 0147 410.

Yours Sincerely

The StatinWISE Team

statinwise.lshtm.ac.uk
